# Using the Theozyme Model to Study the Dynamical Mechanism of the Post-Transition State Bifurcation Reaction by NgnD Enzyme

**DOI:** 10.3390/molecules29235518

**Published:** 2024-11-22

**Authors:** Yaning Hou, Jingyun Chen, Weizhe Liu, Gaohua Zhu, Qianying Yang, Xin Wang

**Affiliations:** Henan-Macquarie University Joint Centre for Biomedical Innovation, Henan Key Laboratory of Brain Targeted Bio-Nanomedicine, School of Life Sciences, Henan University, Kaifeng 475004, China; ynhou@henu.edu.cn (Y.H.);

**Keywords:** post-transition state bifurcation, theozyme model, bifurcation selectivity

## Abstract

Post-transition state bifurcation (PTSB) is a fundamental process in which a single transition state leads to multiple products. This phenomenon is important in both biological and chemical contexts and offers valuable insights into reaction mechanisms and their applications. The theozyme model, which focuses on key residues within enzymes, offers a computationally efficient method for studying these processes while preserving the enzyme’s catalytic properties. This approach enhances our understanding of how enzymes stabilize and direct the transition state, thereby influencing product distribution and selectivity. In this study, we investigate the dynamics and regulatory mechanisms of the PTSB reaction catalyzed by the enzyme NgnD. The enzyme NgnD facilitates a cycloaddition reaction that produces both [6 + 4] and [4 + 2] adducts, with a preference for the [6 + 4] adduct. By analyzing the potential energy surface, bond length distribution, and interactions between the theozyme and the ambimodal transition state, we elucidate the role of the enzyme’s active site residues in determining product selectivity. We illustrate how these key residues contribute to the formation of different adducts, providing insights from various perspectives. Using theozyme models, we propose how the four most influential active residues collectively might control the direction of adduct formation through their cumulative effects.

## 1. Introduction

Enzymes act as highly efficient and specific catalysts, playing a crucial role in the chemical reactions vital for metabolic and regulatory processes in living organisms [1,2]. Understanding enzyme-catalyzed mechanisms is crucial for advancing biochemistry and bioengineering. This includes exploring the factors influencing catalytic efficiency and selectivity, as well as designing or improving enzymes. A key area of investigation focuses on how conformational changes at various timescales, from nanoseconds to sub-picoseconds, affect enzymatic reactivity and selectivity. While nanosecond-scale motions are well-documented in many enzymes, the role of faster, sub-picosecond movements in modulating enzyme function is less understood. These ultrafast movements of amino acids in the active site can directly affect the stabilization of the transition state and, consequently, the reaction outcomes. Computational simulations are indispensable for probing these intricate processes. Methods such as classical molecular dynamics (MD), quantum mechanics/molecular mechanics (QM/MM), quasi-classical trajectory simulations, transition state theory, and free energy calculations enable detailed studies of enzyme-catalyzed reactions at the molecular level, providing detailed insights into the reaction pathways of enzyme-catalyzed reactions. However, these computational methods are often hampered by high costs owing to the complex nature of enzyme structures and the need to simulate long timescales or large systems.

To address these challenges, Professor K. N. Houk introduced the theozyme model [3,4,5,6], a powerful approach that simplifies enzyme systems for quantum mechanical analysis. This method involves constructing a theozyme model by selecting catalytic moieties based on the enzyme’s structure and function and using quantum chemical methods such as Density Functional Theory (DFT) [7] or Hartree–Fock (HF) [8] theory to analyze the transition state structures and energies. This approach provides a detailed picture of enzyme-catalyzed reactions, including substrate orientation, bond dynamics, and molecular rearrangements [5,9,10,11,12,13,14,15,16,17,18,19,20]. The theozyme model is particularly effective in isolating the interactions between specific amino acids and substrates, providing high-precision insights into the underlying mechanisms of enzyme catalysis [21,22,23].

**Figure 1:**
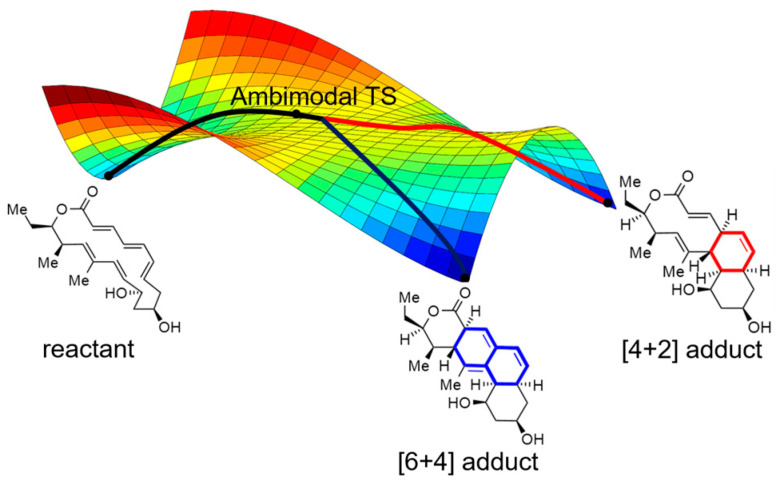
Post-transition state bifurcation potential energy surface with an ambimodal TS, the red line represents the route that generates the [4 + 2] adduct, and the blue line represents the route that generates the [6 + 4] adduct.

The theozyme model has proven to be invaluable for studying complex enzymatic reactions. In our study, we applied this model to explore the post-transition state bifurcation (PTSB) reaction catalyzed by the NgnD enzyme and gain new insights into its mechanism. Specifically, PTSB occurs when a single transition state (TS) branches into multiple product pathways [24,25,26,27] (Figure 1). This phenomenon has been extensively studied [28,29,30,31,32,33,34,35,36,37,38,39,40,41], particularly in enzyme-catalyzed cycloaddition reactions, such as Spinosyn A biosynthesis [33,42], stereoselective dehydration [43,44], and streptomycin biosynthesis [45]. The enzyme NgnD, which was first identified in studies focused on biosynthetic pathways responsible for producing natural products with medicinal properties, catalyzes cycloaddition reactions, providing an ideal model for studying PTSB. It plays a crucial role in biosynthesis by catalyzing both [6 + 4] and [4 + 2] adduct formations from an ambimodal transition state in a cycloaddition reaction. Notably, NgnD exhibits a surprising preference for the [6 + 4] adduct, a pathway less common in organic chemistry, sparking interest owing to its unique selectivity. Understanding how NgnD directs this unusual product distribution requires a detailed examination of the enzyme’s active site and the dynamic interactions that guide product formation.

Previous studies on the NgnD-catalyzed PTSB reaction have employed kinetic analyses, biochemical experiments, and computational simulations to explore the influence of active site residues on product selectivity. In 2019, Ge, Liang, and Tan used computational and biochemical methods to analyze the catalysis of [6 + 4]/[4 + 2] cycloaddition [45], identifying key residues, such as W67, Y55, M69, and Y13, which affect the [6 + 4] product. However, traditional methods for calculating reaction trajectories, such as single-point energy calculations, transition state theory, molecular dynamics simulations, and calculations using Singleton’s ProgDyn program (https://github.com/DanielSingleton/Progdyn, accessed on 1 September 2023) [46,47,48,49], often require significant computational resources and do not fully account for environmental effects. To address these limitations, Ge, Liang, and Yang enhanced the theozyme model in 2021 by integrating it with the Environmental Perturbation Theory of Sampling of States (EPTSS) [31]. By leveraging the potential energy surface to account for environmental perturbations, they were able to sample the conformations of transition states more effectively. This study investigated the influence of water and enzyme surroundings on the NgnD-catalyzed PTSB reactions, uncovering the crucial role of environmental factors in controlling the chemoselectivity of biosynthesis reactions.

**Figure 2:**
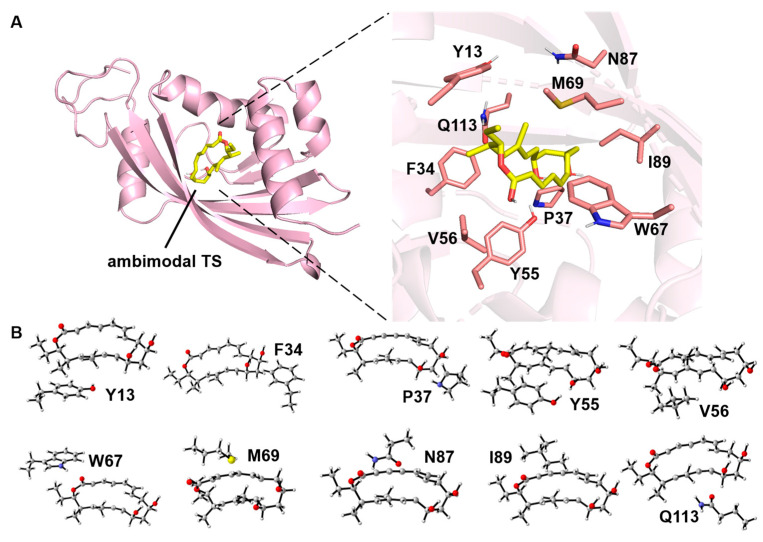
(**A**) Binding diagram of the ambimodal transition state to NgnD and the structures of the active site residues (carbon atoms are labeled in salmon) and the DFT-optimized ambimodal TS (carbon atoms are labeled in yellow). The PDB code of NgnD is 6A5F. (**B**) Theozyme models constructed sequentially from the ten active amino acid residues. The C atoms are gray, the H atoms are white, the O atoms are red, the S atom is yellow, and the N atoms are bule.

In this study, we systematically investigated the NgnD enzyme-catalyzed PTSB reaction of [6 + 4] and [4 + 2] cycloaddition using the theozyme model. Our study employed a combination of ab initio molecular dynamics simulations and quantum chemical calculations to analyze the substrate transition states catalyzed by NgnDase in detail. We focused on the kinetic and dynamic roles of ten key amino acid residues in the active site: Y13, F34, P37, Y55, V56, W67, M69, N87, I89, and Q113 (Figure 2). This comprehensive approach not only considers the influence of the surrounding medium but also quantifies the specific contributions of each active residue to product selectivity. By overcoming the limitations of previous methodologies, we provide more precise and efficient insights into NgnD enzyme-catalyzed [6 + 4]/[4 + 2] cycloaddition and subsequent PTSB reactions. This study advances our understanding of these processes and offers a new perspective for the design and optimization of enzyme-catalyzed cycloaddition reactions.

## 2. Results and Discussion

### 2.1. Analysis of Energy Barriers

**Figure 3:**
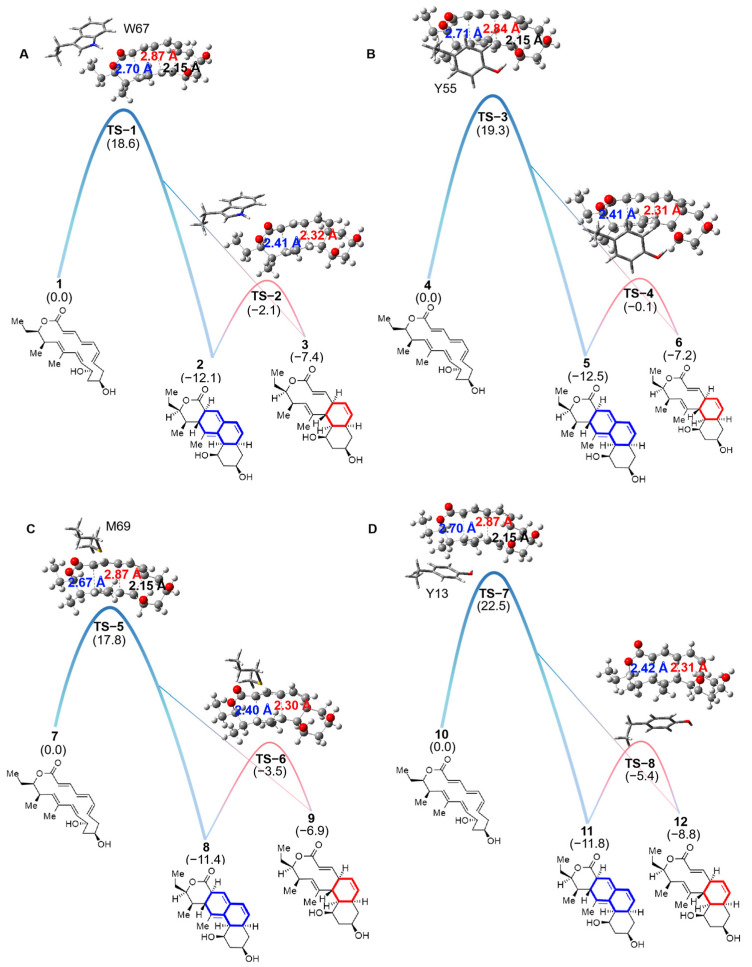
The Gibbs free energy was calculated by DFT for the [6 + 4] and [4 + 2] cycloaddition and [3,3]-Cope rearrangement reactions. The theozyme model consisting of W67 (**A**), the theozyme model consisting of Y55 (**B**), the theozyme model consisting of M69 (**C**), and the theozyme model consisting of Y13 (**D**) participated in the formation of [6 + 4] and [4 + 2] adducts from reactants through TS, and the two adducts can interconvert via the cope rearrangement. The level of theory was calculated as CPCM(diethyl ether)-B3LYP-D3/6-311+G(d,p)//B3LYP-D3/6-31G(d), and the number in parentheses is the calculated Gibbs free energy value in kcal/mol.

In the NgnD enzyme-catalysed [4 + 2]/[6 + 4] cycloaddition reaction, the key active site residues critically influence product formation. As the reaction progresses through the transition state, it encounters a bifurcation point that determines whether the [6 + 4] or [4 + 2] adducts are formed. The results of quasi-classical molecular dynamics simulations indicated a tendency towards the formation of the [6 + 4] adduct. The energy barrier for ten different theozyme models was calculated, with specific values detailed in Figure S1 and Table S1. Among these models, we focus on the most significant four models for analysis. Figure 3 illustrates the bifurcation process. The formation of the [6 + 4] adduct is favoured by shorter C-C bond distances in the ambimodal TS (highlighted in blue), whereas longer C-C bond distances (highlighted in red) indicate the [4 + 2] adduct pathway. Theoretical calculations provide quantitative insights into the energy barriers and Gibbs free energies associated with these adducts, thereby revealing their thermodynamic stability and reaction pathways. In particular, the Gibbs free energies of the [6 + 4] and [4 + 2] adducts were examined in theozyme models featuring the key residues W67, Y55, M69, and Y13. As illustrated in Figure 3, the data predict that each active site residue significantly influences the stability of the adducts. The Gibbs free energies of the [6 + 4] adducts were consistently lower than those of the [4 + 2] adducts, which possess greater thermodynamic stability. In theozyme models featuring W67, Y55, M69, and Y13, the Gibbs free energy differences for the [6 + 4] and [4 + 2] adducts were 4.7, 5.3, 4.5, and 3.0 kcal/mol, respectively. This also indicates a strong thermodynamic preference for the formation of [6 + 4] adducts.

Previous studies [31] have identified an energy barrier of 23.1 kcal/mol for the gas-phase-catalyzed [6 + 4] and [4 + 2] cycloaddition reactions involving the enzyme NgnD. However, when theozyme models with key residues (W67, Y55, M69, Y13) were used in an ether environment, the energy barriers decreased significantly to 17.8–22.5 kcal/mol (Figure 3). This reduction emphasizes the active site’s role in lowering the energy barriers and enhancing the reaction efficiency. These key residues consistently affect the formation of both adducts, highlighting their crucial involvement in guiding the reaction pathway and favoring the [6 + 4] adduct formation.

### 2.2. Bond Length Distributions

**Figure 4:**
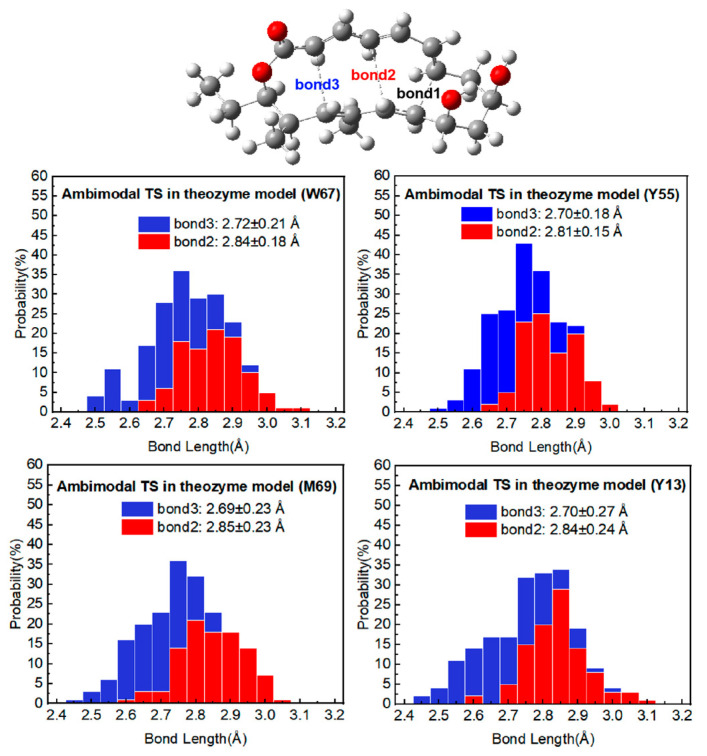
Distribution of bond 2 and bond 3 in 100 TS geometries for ambimodal TS from four theozyme models in diethyl ether. Bond 2 in red leads to the [4 + 2] adduct, while bond 3 in blue leads to the [6 + 4] adduct.

To investigate the influence of specific active site residues on the geometry of ambimodal transition states in the NgnD-catalyzed [6 + 4] and [4 + 2] cycloaddition reactions, we performed a quasi-classical trajectory simulation for each enzyme model, sampling the TSs geometry in 100 complete trajectories. Bond 2 represents the [4 + 2] adduct, whereas bond 3 corresponds to the [6 + 4] adduct. Figure 4 shows the distribution of bond lengths for bond 2 and bond 3 in the ambimodal TS across the theozyme models. The blue bars represent the [6 + 4] adduct, and the red bars represent the [4 + 2] adduct. In our previous research on gas-phase bimodal transition states (TSs), we established the average bond lengths for bond 2 and bond 3 to be 2.85 ± 0.27 Å and 2.70 ± 0.21 Å, respectively. In the theozyme models, the average bond lengths for bond 2 were 2.84 ± 0.18 Å (W67), 2.81 ± 0.15 Å (Y55), 2.85 ± 0.23 Å (M69), and 2.84 ± 0.24 Å (Y13). For bond 3, the average lengths were 2.72 ± 0.21 Å (W67), 2.70 ± 0.81 Å (Y55), 2.69 ± 0.23 Å (M69), and 2.70 ± 0.27 Å (Y13). The comparison of the bond lengths in the enzyme environment with those in the gas phase revealed that bond 2 lengths were typically shorter, while bond 3 lengths are generally longer (with the exception of the M69 system). Furthermore, bond length 3 was consistently shorter than bond length 2 in all four theozyme models. These findings correlate with the established relationship between the bond lengths and the distribution ratios of the [6 + 4] and [4 + 2] adducts in the PTSB reaction. This relationship, previously identified by Yang Zhongyue et al. [32], suggests a preference for the formation of [6 + 4] adducts over [4 + 2] adducts. The current study supports this correlation and validates the use of theozyme models to accurately simulate this reaction in an enzymatic environment. Of particular interest is the role of residues W67 and Y55, which our simulations demonstrate to produce [4 + 2] adducts with bond lengths of less than 2.85 ± 0.27 Å, a notable difference when compared to the other residues. These observations highlight the significant variation in bond lengths observed. Given that shorter bond lengths indicate a greater propensity for the formation of adducts, it can be inferred that the two residues, W67 and Y55, exert a more significant influence. It should be noted that there are subtle differences in the effects of the various key residues on the distribution of [6 + 4] and [4 + 2] adducts. The specific effects on adduct ratios will be discussed in the next section.

### 2.3. Analysis of Kinetic Trajectory and Dynamics

**Figure 5:**
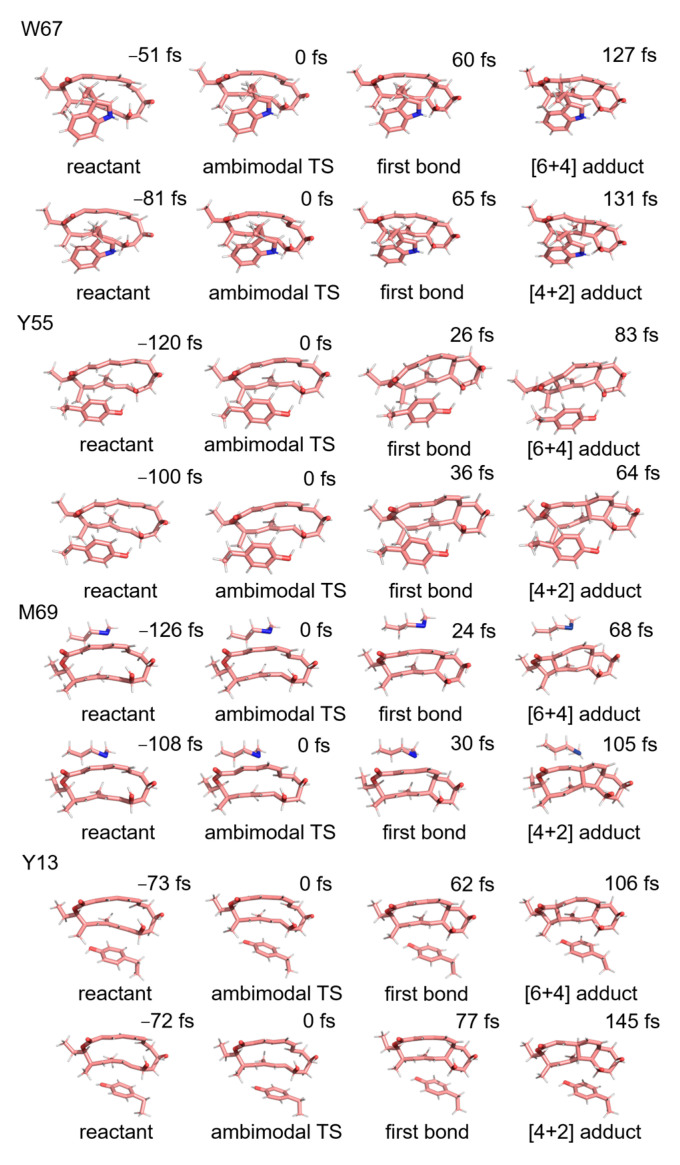
Typical trajectories for the formation of [6 + 4] adducts and [4 + 2] adducts in theozyme models consisting of W67, M69, Y55, and Y13, respectively (1.7 Å is the criterion for C-C bond formation).

**Figure 6:**
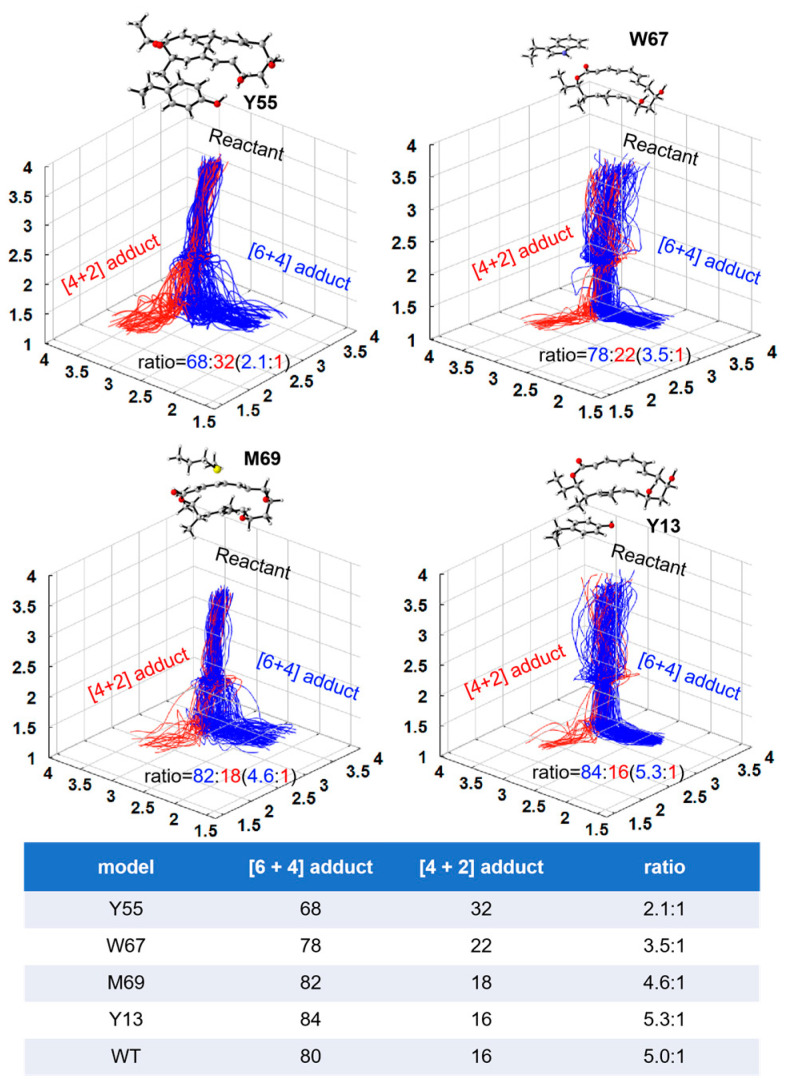
Distribution of reactive trajectories initiated from ambimodal TS by four theozyme models in implicit diethyl ether. One hundred randomly selected trajectories were plotted in each case. Blue lines represent the [6 + 4] adducts, and red lines represent the [4 + 2] adducts. The table shows the ratio of [6 + 4] and [4 + 2] adducts. The first four rows of data are the theozyme model in this paper, and WT is the ambimodal TS model without the enzyme previously studied [31].

This section presents an analysis of the influence of the key residues on the kinetic trajectory and dynamics of the [6 + 4]/[4 + 2] cycloaddition reaction. Kinetic mechanisms of the NgnD enzyme-catalysed [4 + 2] and [6 + 4] cycloaddition reactions using quasi-classical trajectory simulations. The primary objective was to understand the influence of specific active site residues on the formation of the [6 + 4] and [4 + 2] adducts. Theozyme models incorporating the key residues W67, Y55, M69, and Y13 were employed to analyze the formation of [6 + 4] and [4 + 2] adducts. We use the capptraj [50] module from AmberTools to process and analyze the valid trajectories. Figure 5 illustrates the randomly selected trajectories for both types of adducts. The criteria for adduct formation were based on the length of the bond: for the formation of an adduct to occur, bond 1 and either bond 2 or bond 3 must simultaneously be less than 1.7 Å. To quantify the process, the time interval was defined as the difference between the formation time of bond 1 and that of bond 2 or bond 3. This analysis was applied to 100 instances of adduct formation across the various theozyme models. Table 1 presents the distribution of the product formation time intervals for each of the 100 adducts derived from quasi-classical trajectory simulations (See Figure S2 for details). The average time intervals for [6 + 4] adduct formation were 66, 79, 77, and 57 fs for W67, Y55, M69, and Y13, respectively. For [4 + 2] adduct formation, the average intervals were 87, 78, 69, and 79 fs, respectively. A significant observation emerged from these data: in an ether environment, the formation times for the [6 + 4] adducts were relatively consistent across different residues, suggesting a stable and robust formation mechanism. In contrast, the formation times for [4 + 2] adducts showed greater variability, indicating that their formation is more sensitive to specific residues and environmental conditions. This suggests a more adaptable and less predictable pathway for the [4 + 2] adducts compared to the [6 + 4] adducts in these theozyme models. Figure 6 shows the kinetic trajectories of the quasi-classical trajectory simulations for the four residues (W67, Y55, M69, and Y13) within the theozyme models at the TS. The trajectories for [6 + 4] adduct formation are depicted in red, while those for [4 + 2] adduct formation are shown in blue. By observing and counting the reaction trajectories in each theozyme model, we determined the [6 + 4] adduct to [4 + 2] adduct ratio. The ratio is 2.1:1 for Y55, 3.5:1 for W67, 4.9:1 for M69, and 5.3:1 for Y13. These ratios are lower than those obtained in the ether environment without using the theozyme model, indicating that these four residues play a key role in controlling the adduct distribution of the [6 + 4]/[4 +2] reaction catalyzed by NgnD and can be well reconciled with previous experimental results [45]. This enhances the effectiveness of the theozyme model in an accurate reproduction of the enzyme’s catalytic environment and can save computational resources significantly, improve computational efficiency, and is described in more detail in the next section.

### 2.4. Interaction of Key Residues with Tss

Previous structural analyses showed that bond lengths in bonds 2 and 3 within different theozyme models exhibited less variation than their gas-phase counterparts. This observation indicates that the primary factors influencing the reaction energy barriers and adduct selectivity are not the geometries of the ambimodal TS but rather the specific interactions between the reacting molecules and active site residues. The analysis shows that residue Y55 engages in hydrogen bonding and CH–π interactions with TS, stabilizing the transition state. The negatively charged trienyl ester segment of the ambimodal TS is stabilized through a T-shaped π–π stacking interaction with the aryl ring of the theozyme model. Additionally, the electron-rich sulfur atoms of M69 are oriented towards the positively charged dienyl portion of the TS, providing further stabilization through electrostatic interactions. Quasi-classical trajectory dynamics simulations indicate that environmental polarity significantly affects the ratio of [6 + 4] to [4 + 2] adducts. In an ether environment, theozyme models containing residues W67, Y55, and M69 exhibited a significantly lower [6 + 4]/[4 + 2] ratio than those in aqueous environments. This suggests that the [6 + 4] adduct, which has a large dipole moment, is more sensitive to environmental polarity changes, which can markedly alter its rate of formation. In contrast, Y13’s participation in the reaction shows only a slight increase in the [6 + 4]/[4 + 2] adduct ratio. This minimal impact is likely due to the weaker and less orientation-specific CH–π interactions associated with Y13. As a result, Y13 has a limited effect on the reorientation of reactants during the TS, making it less effective in controlling the adduct ratio than the other residues. The combined interactions of residues Y13, W67, M69, and Y55 with TS significantly affected reaction kinetics. Each residue contributes uniquely to stabilizing the TS and collectively ensures a shift in the final product distribution towards the [4 + 2] adduct. This cumulative effect underscores the importance of specific residue interactions in determining the kinetic outcomes of NgnD-catalyzed reactions. An analysis of the electrostatic potentials of the DFT-optimized TS structures, detailed in the Supplementary Material (Figure S3), further enhances our understanding of these interactions. These insights are critical for the future modification of NgnD and similar cycloaddition enzymes. By fine-tuning active site residues, it may be possible to tailor enzyme activity for specific adduct formations, providing valuable guidelines for enzymatic design and transformation applications.

## 3. Computational Methods

### 3.1. Theozyme Model Construction

To investigate the PTSB mechanisms catalyzed by the NgnD enzyme, we constructed simplified theozyme models incorporating the following key active residues: F34, M69, Y13, P37, Q113, W67, V56, I89, Y55, and N87. These residues were strategically positioned to interact with TS atoms, forming the core of our theozyme models. To maintain the structural integrity and prevent collapse or deformation, we fixed the number of truncated Cα atoms at the model boundaries. Truncated amino acids were linked at C-C single bonds and hydrogen-saturated at the truncated ends, with non-Cα backbone atoms removed. This approach ensured that our theozyme models accurately represented the enzyme’s active site while simplifying the overall structure for computational feasibility [51,52,53,54].

### 3.2. Molecular Dynamics Simulation

Molecular dynamics (MD) simulations were performed using AmberTools 20 software [55] to explore the dynamic behavior of the enzyme and its interaction with the substrate and transition states. For these simulations, we applied the RESP model [56] to the transition state at the HF/6-31G(d) level. The charges were determined using the Merz–Singh–Kollman scheme [56,57], with computations carried out using the Gaussian 16 Rev. A. 03 software package [58]. We applied the General Amber Force Field (GAFF) [59] for the substrate and TS structures and the FF99SBildn force field [60] for the protein residues. Water molecules were modeled using the TIP3P force field [61]. The enzyme–substrate system was initially minimized over 20,000 steps to remove steric clashes and then gradually heated to 300 K. Equilibration was achieved over 100 ps under constant pressure (NPT ensemble), followed by a 500 ns production run under constant volume (NVT ensemble) conditions.

### 3.3. Quantum Mechanical Calculations

Quantum mechanical (QM) calculations were performed using the Gaussian 16 Rev. A. 03 program package [58] to optimize the geometries of the small molecules and assess the reaction energy barriers. We employed initial molecular geometry optimization at the B3LYP-D3/6-31G(d) level. For accurate energy barrier calculations of the theozyme-substrate systems, we used the B3LYP-D3 level [7] of density functional theory (DFT) with the 6-311+G(d,p) basis set. To elucidate the influence of solvent effects on the [6 + 4]/[4 + 2] cycloaddition reaction, we first employed the relatively time-consuming solvation model dynamics (SMD) method [62] and constructed implicit solvent fields for ten theozyme models using water as the solvent to conduct preliminary theozyme model studies. Based on previous findings, we selected four key residues—W67, Y55, M69, and Y13—that had the strongest stabilizing interactions and the most significant effect on the reaction. To more accurately simulate the enzyme’s microenvironment without the computational complexity of an all-atom enzyme model, we employed the continuous polarization medium model (CPCM) [63,64,65] with diethyl ether as the implicit solvent. Diethyl ether, with its dielectric constant of 4.2, closely approximates the dielectric environment in the enzyme. Using diethyl ether allows us to effectively capture the essential solvent effects that mimic the enzyme’s microenvironment without resorting to a full atomic-level solvent model.

### 3.4. Quasi-Classical Trajectory Simulations

To investigate the reaction dynamics in detail, we employed quasi-classical trajectory simulations, initializing the energy surface near the transition state at the B3LYP-D3 level with the 6-31G(d) basis set. Using the zero-point vibrational energy (ZPE) of the TS normal mode and a thermodynamic probability distribution at 298 K, we performed stochastic Boltzmann sampling of the initial conditions. The molecular dynamics trajectories were simulated using Singleton’s Progdyn package (https://github.com/DanielSingleton/Progdyn, accessed on 1 September 2023) [49], with an integration time step of 1 fs. This allowed us to simulate both forward and backward reactions, with forward simulations leading to the formation of either the [6 + 4] or [4 + 2] adduct, while backward simulations regenerated the reactants.

By combining forward and backward trajectories, we reconstructed a complete reaction pathway. Additionally, we applied filtering to remove any trajectory recrossing events, ensuring the accuracy of the simulation results. In each of the theozyme models (Y55, W67, M69, Y13), the sum of [6 + 4] and [4 + 2] adducts reached 100, confirming the consistency of the results. This comprehensive approach provided a detailed understanding of the PTSB dynamics catalyzed by the enzyme NgnD. The quasi-classical trajectory simulations thus offered a high-resolution view of how both the reaction products and the reactants evolve over time, providing critical insights into the reaction mechanism.

## 4. Conclusions

In this study, we utilized the theozyme model in combination with quasi-classical trajectory simulations to investigate the impact of specific amino acid residues on the post-transition state bifurcation in NgnD enzyme-catalyzed [6 + 4] and [4 + 2] cycloaddition reactions. Our detailed analysis highlights several key findings regarding the influence of these residues on the reaction outcomes. First, we observed that the shape of the potential energy surface of the reaction, the bond lengths within the transition state, and the timing and spatial interactions significantly affected the ratio of [6 + 4] to [4 + 2] adducts. These factors collectively determine the bifurcation pathway and, ultimately, the distribution of the resulting adducts. This approach offers a streamlined and efficient alternative to the traditional methods. By simulating complex enzyme environments directly through theozyme models, the specific roles of key amino acid residues can be rapidly evaluated, ensuring consistency with experimental data. This approach, which substitutes full-atom modeling of large enzyme systems, not only circumvents the significant computational costs associated with QM/MM methods but also enables a swift and precise assessment of how different residues influence the reaction pathways [4,5,6,9,14]. Additionally, our investigation into the factors that influence the adduct ratio in the PTSB reaction aligns well with the previous experimental observations [31,45]. We identified particular residues that exerted a more pronounced effect on the distribution of adducts, providing insights that may be pivotal for future enzyme engineering.

## Figures and Tables

**Table 1:** Averaged time gap (fs) for adduct generation in four theozyme models.

Theozyme Model	[6 + 4] Adduct	[4 + 2] Adduct
W67	66	87
Y55	79	78
M69	77	69
Y13	57	79

## Data Availability

The original contributions presented in the study are included in the article/Supplementary Materials, further inquiries can be directed to the corresponding author.

## Supplementary Materials

The following supporting information can be downloaded at https://www.mdpi.com/article/10.3390/molecules29235518/s1, Figure S1: Gibbs free energy calculations for 10 theozyme models. Figure S2: Distribution of time gap of different adducts in four theozyme models. Figure S3: The electrostatic potential analysis. Table S1: Gibbs free energy of 10 theozyme models. Data S1: DFT-computed energies and Cartesian coordinates (see [66]).
