# Supplementary material for: Using the Theozyme Model to Study the Dynamical Mechanism of the Post-Transition State Bifurcation Reaction by NgnD Enzyme
Source: Molecules. 2024 Nov 22;29(23):5518. doi: 10.3390/molecules29235518 (PMC11643694; doi:10.3390/molecules29235518)
Supplement: Supplementary file 1 [file molecules-29-05518-s001.zip › molecules-3246066-supplementary.pdf]

Supplementary Materials

Using the Theozyme Model to Study the Dynamical Mechanism of the Post-Transition State Bifurcation Reaction by NgnD Enzyme

Yaning Hou †, Jingyun Chen †, Weizhe Liu, Gaohua Zhu, Qianying Yang and Xin Wang *

Henan-Macquarie University Joint Centre for Biomedical Innovation, Henan Key Laboratory of Brain Targeted Bio-Nanomedicine, School of Life Sciences, Henan University, Kaifeng 475004, China; ynhou@henu.edu.cn (Y.H.)

* Correspondence: wx@henu.edu.cn

† These authors contributed equally to this work.

Table of Contents

I. Computational Methodologies S2
II. Supplementary Figures and Table S4
Figure S1. Gibbs free energy calculations for 10 theozyme models S6
Figure S2. Distribution of time gap of different adducts in four theozyme models S7
Figure S3. The electrostatic potential analysis S9
Table S1. Gibbs free energy of 10 theozyme models S9
III. Supplementary Data S1. DFT-computed energies and Cartesian coordinates S10
References S101

I. Computational Methodologies

Theozyme model construction. We selected key active residues F34, M69, Y13, P37, Q113, W67, V56, I89, Y55, and N87 based on the enzyme NgnD structure (PDB: 6A5F). These catalytic groups were then placed near the vicinity of the transition state (TS), respectively, to form the optimal interactions, constructing ten simplified theozyme models. To maintain structural consistency with the original crystal structure and prevent deformation, we fixed the number of truncated alpha carbon (CA) atoms at the model boundary. In truncating amino acids, we chose to truncate between C-C single bonds. Subsequently, we manually hydrogen-saturated the truncated bonds and removed other protein backbone atoms other than the CA atoms. This approach ensured the model's realism and accuracy, closely mimicking the actual enzyme structure.

Molecular dynamics (MD) simulations. MD simulations were performed in explicit water using the Amber20 package[1]. Ten theozyme models were used for these simulations, assigning RESP model[2] to the transition state at the HF/6-31G(d) level. The charges were calculated using the Merz–Singh–Kollman scheme[3, 4] with the Gaussian 16 Rev. A. 03 software package[5]. The General Amber force field (GAFF)[6] was applied to substrate and transition state structures, while the FF99SBildn[7] force field was used to describe protein residues. The theozyme models were solvated in a pre-equilibrated cubic box with a 10 Å buffer of TIP3P[8] water moleculars using AMBER20 leap module, adding approximately 16,000 solvent molecules. We first performed 20,000 steps of minimization to remove steric clashes, gradually heated the system to 300 K without restraints, equilibrated for 100 ps under the NPT system, and performed the molecular dynamics simulation for 500 ns under the NVT system. The final frame's conformation was selected as the initial conformation for subsequent simulations.

Quantum mechanical calculations. The QM calculations were performed using Gaussian 16 Rev. A. 03 package to optimize the geometries of small molecules and evaluate the reaction energy barriers within the theozyme-substrate systems. Initially, we performed geometry optimizations at the B3LYP-D3/6-31G(d) level of theory. This step provided an accurate starting point for subsequent calculations. To accurately determine the energy barriers of different theozyme models for the [6+4] and [4+2] cycloaddition reactions, we used the B3LYP-D3 level of theory[9] with the the basis set of 6-311+G(d,p). We first employed the solvation model dynamics (SMD) method[10], a more time-consuming but robust approach to simulate solvent interactions. This was applied to construct implicit solvent fields for all ten theozyme models using water as the solvent. Water, being the natural medium for most biological processes, provided an initial benchmark for exploring solvent. To focus our investigation on the most influential residues in the catalytic process, we selected four key active site residues—W67, Y55, M69, and Y13—based on their strong stabilizing interactions with the transition state and their significant impact on the reaction. We then used the continuous polarization medium model (CPCM)[11-13] to simulate implicit solvation in diethyl ether, which has a dielectric constant of 4.2 (which approximates that of the enzyme), thus exploring the effect of a more hydrophobic solvent environment. This solvent better mimics the enzyme's active site microenvironment, where hydrophobic

interactions can play a critical role, allowing us to better capture solvent effects.

Quasi-classical trajectory simulations. The initial energy surface of the system near the transition state was set at the B3LYP-D3 level of theory with the 6-31G(d) basis set. In this state, we used Singleton's Progdyn package[14] to perform molecular dynamics trajectory simulations, combining the zero-point vibrational energy (ZPE) of the transition state normal mode with the thermodynamic probability distribution of the random phase at 298 K to obtain random Boltzmann sampling. The integration time step was set to 1 fs, and forward simulations produced either the [6+4] or [4+2] adduct, while backward simulations regenerated the reactants. By combining the forward and backward simulation trajectories, a complete reaction trajectory was obtained. By filtering to remove recross, we ensured that the sum of [6+4] and [4+2] adducts in each theozyme model (Y55, W67, M69, Y13) reached 100 each.

Data analysis. Trajectory analysis was conducted using capptraj[15] module from Ambertools 20 to process and analyze the valid trajectories in detail, calculating key parameters such as bond lengths and angles, and generating corresponding output files. The distribution graphs of valid trajectories were displayed using Gnuplot. The graphs of 3D structures were rendered using PyMOL (http://www.pymol.org/). Initial molecular structures used for the simulations and the electrostatic potential analysis of the optimized ambimodal transition state structure were performed using GaussView 6.0.16. The time gap and bond length distributions of [6+4] and [4+2] adducts were displayed using the OriginPro (version 2022b; OriginLab, Northampton, MA, USA) software package, giving insights into the conformational flexibility of key residues throughout the reaction.

II. Supplementary Figures and Tables

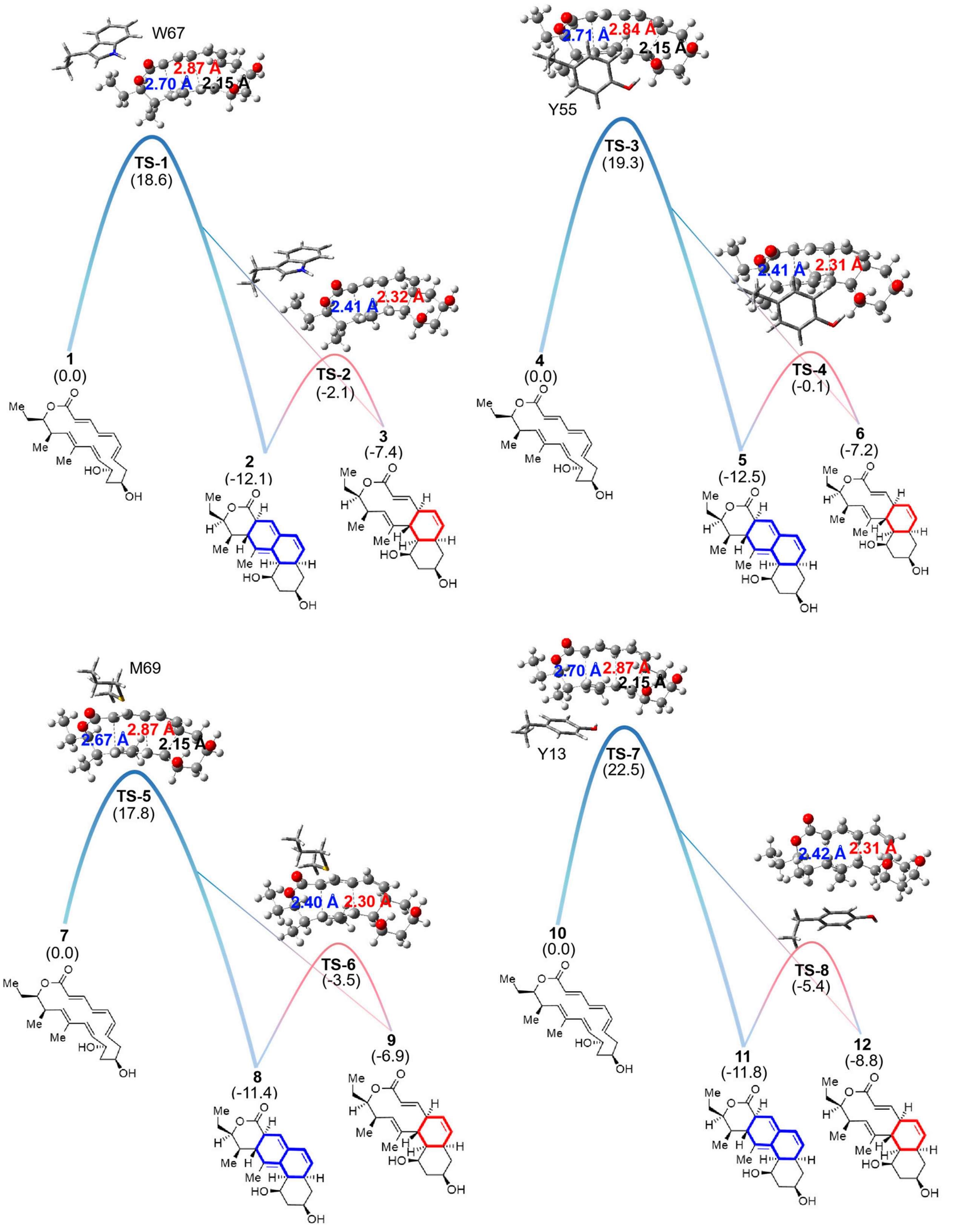

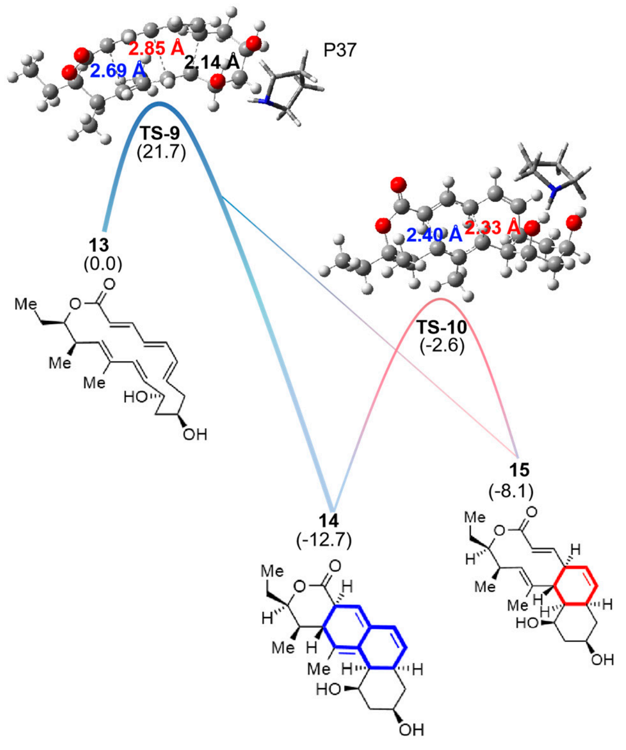

P37
2.85 Å
2.69 Å
2.14 Å
TS-9
(21.7)
13
(0.0)
2.40 Å
2.33 Å
TS-10
(-2.6)
15
(-8.1)
14
(-12.7)

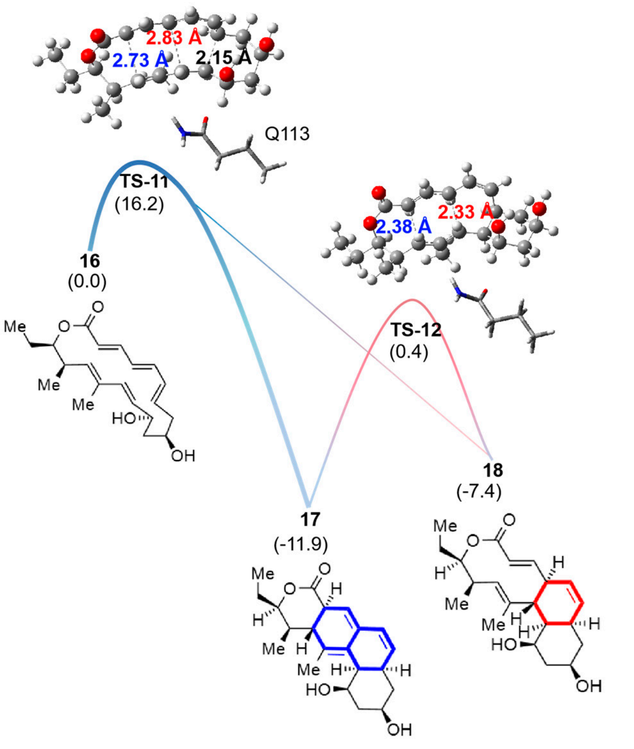

2.83 Å
2.73 Å
2.15 Å
Q113
TS-11
(16.2)
16
(0.0)
2.38 Å
2.33 Å
TS-12
(0.4)
18
(-7.4)
17
(-11.9)

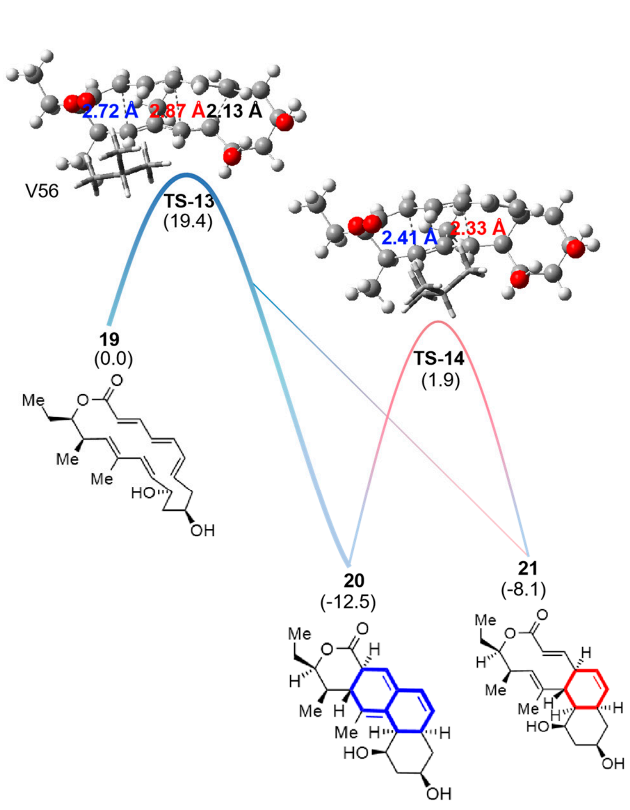

2.72 Å
2.87 Å
2.13 Å
V56
TS-13
(19.4)
19
(0.0)
2.41 Å
2.33 Å
TS-14
(1.9)
21
(-8.1)
20
(-12.5)

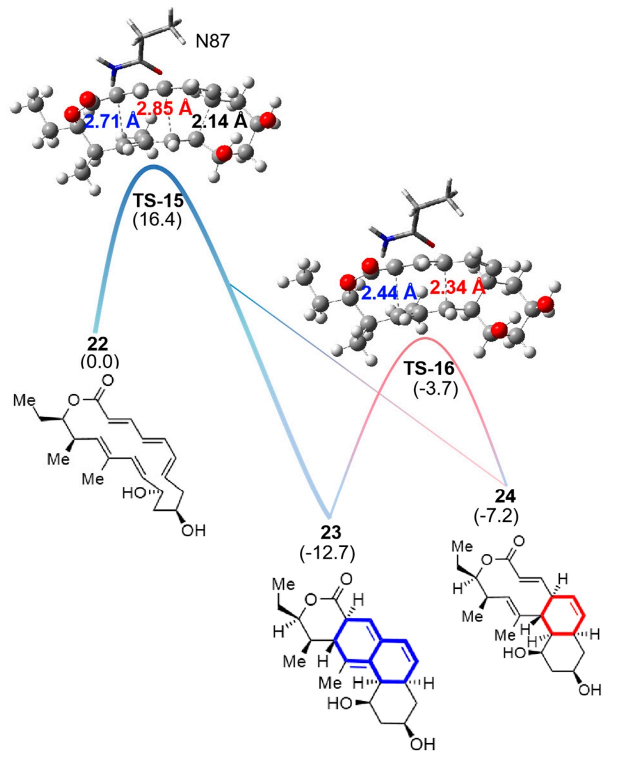

N87
2.71 Å
2.85 Å
2.14 Å
TS-15
(16.4)
22
(0.0)
2.44 Å
2.34 Å
TS-16
(-3.7)
24
(-7.2)
23
(-12.7)

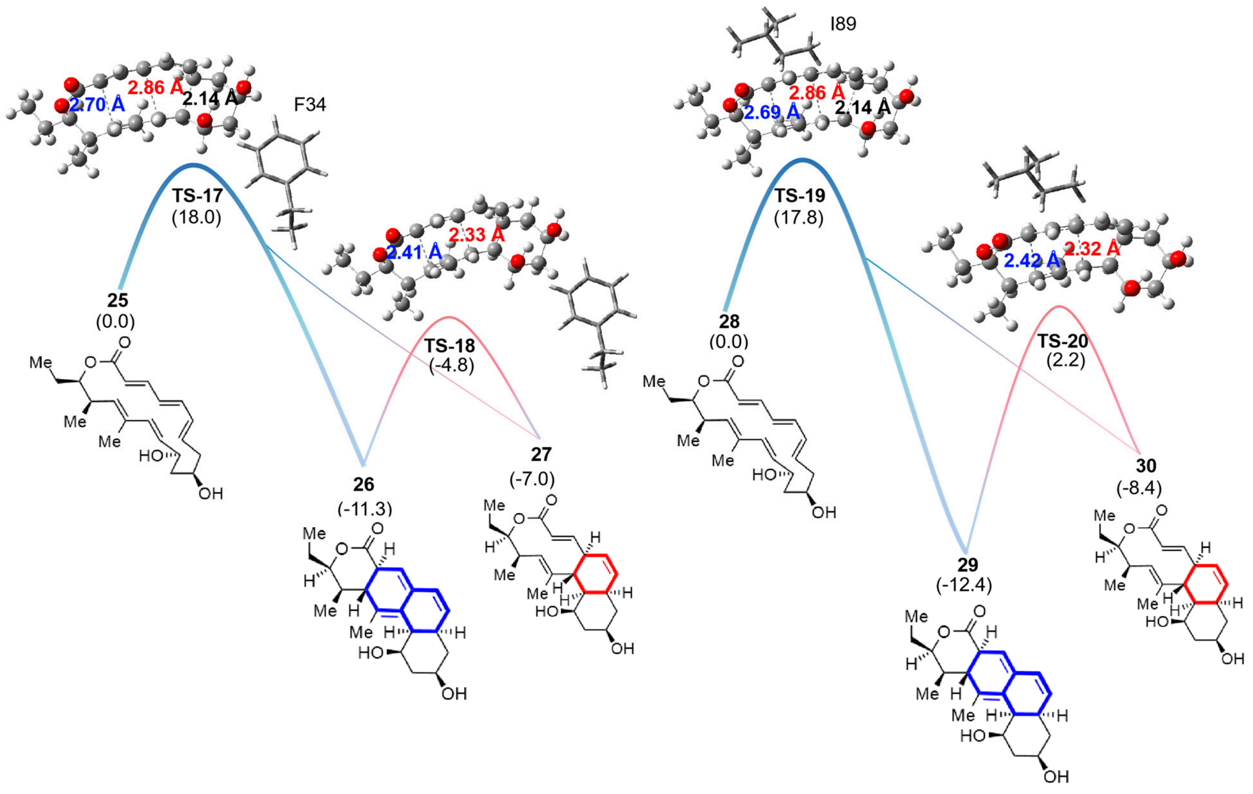

Figure S1. Gibbs free energy calculations for 10 theozyme models. The Gibbs free energies of the [6 + 4] and [4 + 2] cycloaddition reactions and the [3,3]-Cope rearrangement reactions have been calculated by DFT. The level of theory was calculated as CPCM (diethyl ether)-B3LYP-D3/6-311+G(d,p)//B3LYP-D3/6-31G(d), and the number in parentheses is the calculated Gibbs free energy value in kcal/mol. The value of the highest point in each group is the highest energy barrier to be crossed in that reaction.

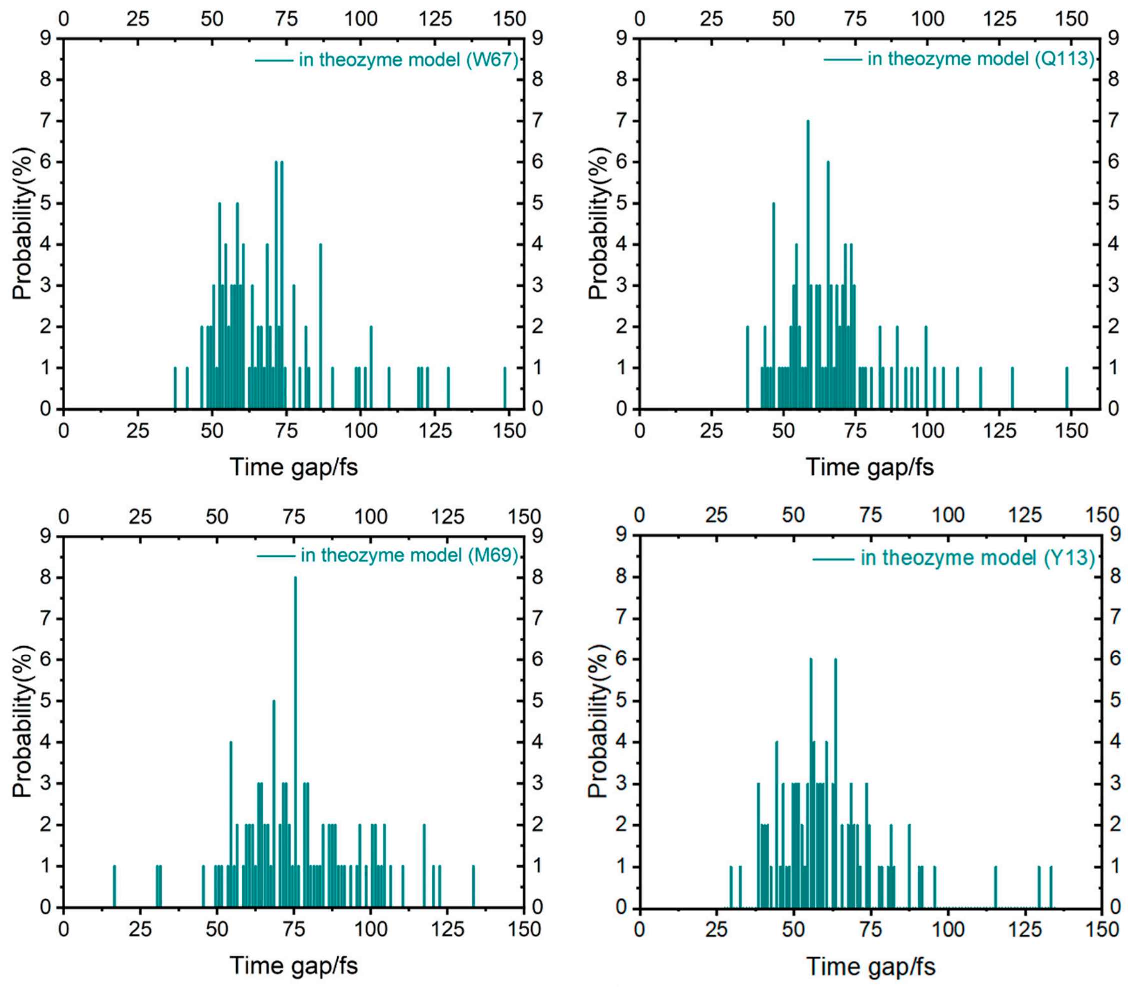

Figure S2. Distribution of time gap for the generation of 100 [6 + 4] adducts and [4 + 2] adducts in four theozyme models. The data were obtained by randomly sampling the time intervals for [6 + 4] and [4 + 2] adduct formation from 100 molecular dynamics trajectories. In the W67 theozyme model, the formation time for [6 + 4] adducts is predominantly around 66 fs, while the [4 + 2] adduct formation time is centered around 87 fs. The Y55 model shows a more even distribution, with [6 + 4] adduct formation times concentrated around 79 fs and [4 + 2] adduct formation times around 78 fs. For the M69 theozyme model, the formation time for [6 + 4] adducts is primarily at 77 fs, whereas the [4 + 2] adduct formation time is around 69 fs. In the Y13 model, the [6 + 4] adduct formation time is mainly around 57 fs, while the [4 + 2] adduct formation time is concentrated around 79 fs.

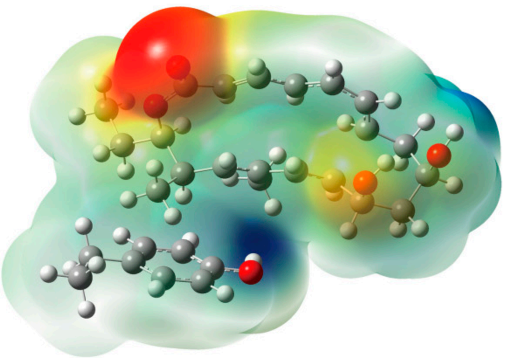

theozyme model (Y13)

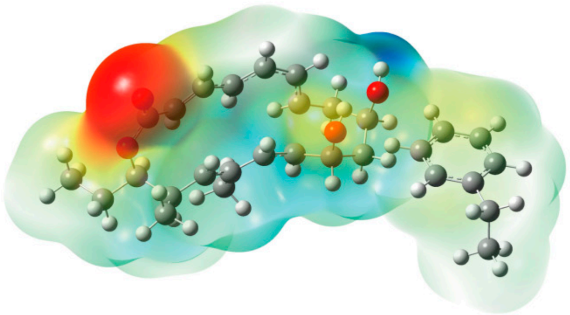

theozyme model (F34)

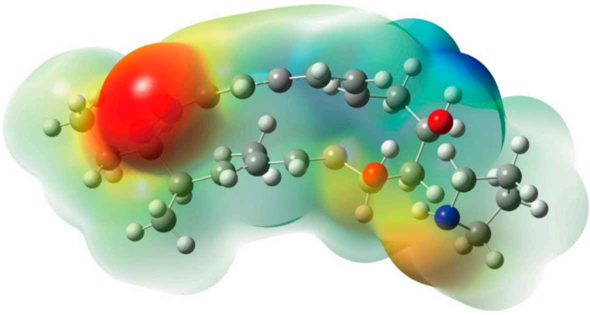

theozyme model (P37)

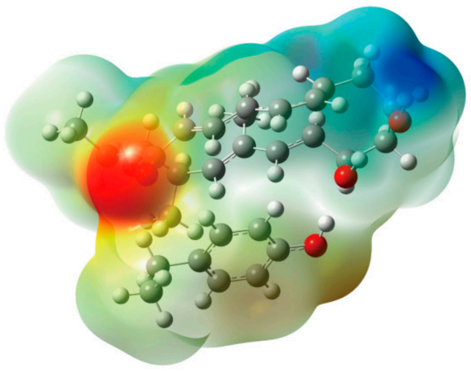

theozyme model (V56)

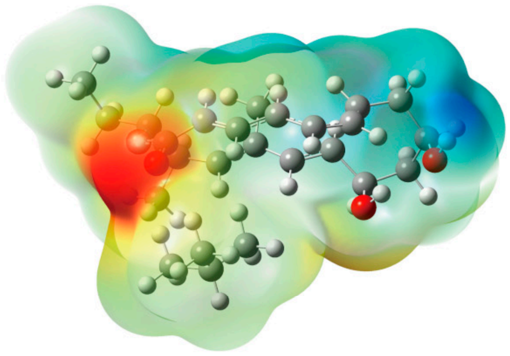

theozyme model (Y55)

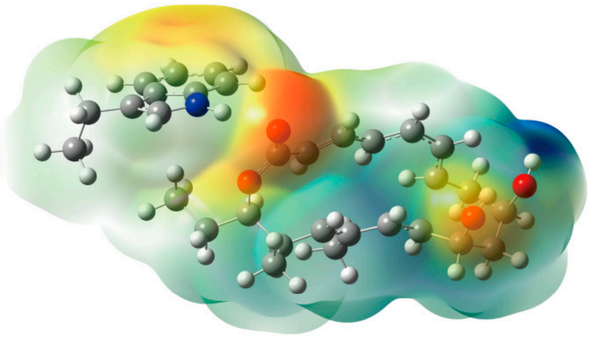

theozyme model (M69)

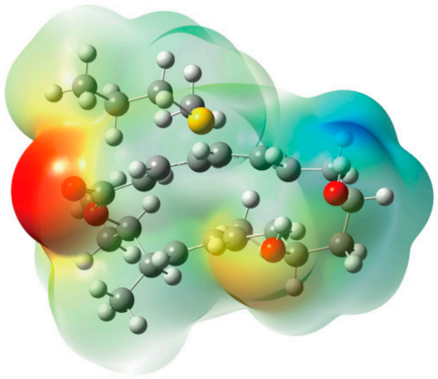

theozyme model (M47)

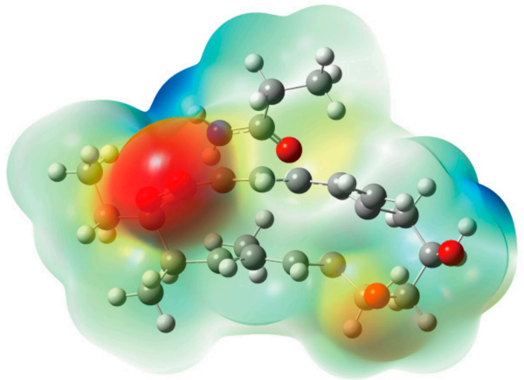

theozyme model (N87)

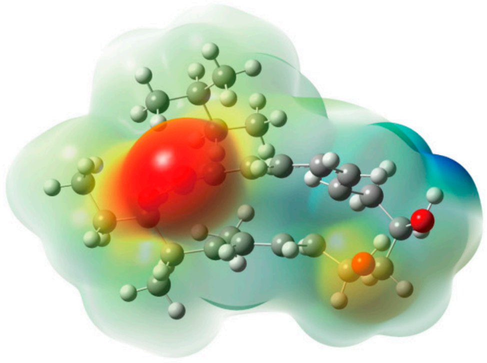

theozyme model (I87)

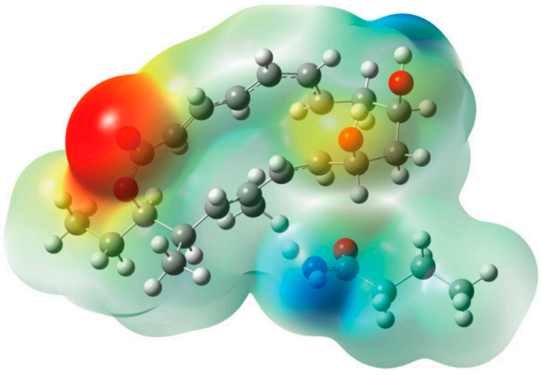

theozyme model (Q113)

Figure S3. The electrostatic potential analysis of the DFT-optimized the ambimodal transition state structures. The areas shaded in blue and red denote regions of electrostatic potential corresponding to negative and positive potentials, respectively, indicating repulsive and attractive interactions with a positive charge. Darker hues indicate an increased magnitude of positive or negative electrostatic potential.

Table S1. Gibbs free energy of 10 theozyme models at the B3LYP-D3/6-31G(d)//B3LYP-D3/6-311+G(d,p) level using the CPCM conductor model (diethyl ether, dielectric constant = 4.2).[a] The geometries were optimized in vacuo.[b]

Theozyme model	CPCM(diethylether)-B3LYP-D3/6-311+G(d,p)//B3LYP-D3/6-31G(d)	Gibbs free energies (in kcal/mol)
W67	**TS-1**	18.6
	**TS-2**	-2.1
	**2**	-12.1
	**3**	-7.4
Y55	**TS-3**	19.3
	**TS-4**	-0.1
	**5**	-12.5
	**6**	-7.2
M69	**TS-5**	17.8
	**TS-6**	-3.5
	**8**	-11.4
	**9**	-6.9
Y13	**TS-7**	22.5
	**TS-8**	-5.4
	**11**	-11.8
	**12**	-8.8
P37	**TS-9**	21.7
	**TS-10**	-2.6
	**14**	-12.7

	**15**	-8.1
Q113	**TS-11**	16.2
	**TS-12**	0.4
	**17**	-11.9
	**18**	-7.4
V56	**TS-13**	19.4
	**TS-14**	1.9
	**20**	-12.5
	**21**	-8.1
N87	**TS-15**	16.4
	**TS-16**	-3.7
	**23**	-12.7
	**24**	-7.2
F34	**TS-17**	18.0
	**TS-18**	-4.8
	**26**	-11.3
	**27**	-7.0
I89	**TS-19**	17.8
	**TS-20**	2.2
	**29**	-12.4
	**30**	-8.4

[a] Gibbs free energies are shown in kcal/mol.

[b] Green boxes represent ambimodal transition states, yellow represents cope rearrangement transition states, blue represents [6+4] adducts, and white represents [4+2] adducts. **1**, **4, 7**, **10**, **13**, **16**, **19**, **22**, **25**, **28,** which are not listed in the table, are the reactants of different theozyme models in top-down order, and their Gibbs free energies are all recorded as 0.0 kcal/mol.

III. Supplementary Data S1. DFT-computed energies and Cartesian coordinates

1

$G_{diethylether}$ = -1561.071538 Hartree

C	-3.26851100	-0.04561300	2.44124900
O	-1.29459100	-2.03824500	-1.28371800
C	-2.78616700	1.09149500	1.53798300
O	-1.67919900	-0.24256600	-0.07470500
C	-1.42430200	0.80072600	0.90098200
O	5.20173600	0.42639100	-1.29767900
C	-0.76918700	2.03096900	0.21400500
O	7.83929100	0.00496400	-1.04939000
C	0.65091900	1.73503000	-0.19168900
C	1.80458200	2.20052800	0.33800500
C	3.07011600	1.70918300	-0.22127600
C	4.30727900	2.11369500	0.11208900

C	5.62831400	-1.66562800	0.54800900
C	5.15962200	-2.75618600	-0.08513900
C	3.74931900	-2.93798000	-0.40935900
C	2.72965700	-2.26010700	0.17348000
C	1.37117400	-2.25738800	-0.29233300
C	0.44227300	-1.40836000	0.20856700
C	-0.87743000	-1.27505200	-0.41832500
C	-1.58451900	2.51459500	-1.00034900
C	5.56226200	1.53089500	-0.48760700
C	6.59579200	1.19102900	0.64517900
C	7.56264500	0.01968300	0.36265600
C	7.05170800	-1.35213600	0.87927600
C	1.88192600	3.18919600	1.47728700
H	-2.59291600	-0.19055700	3.29453700
H	-3.33133700	-0.98266800	1.88255800
H	-4.27015400	0.16405800	2.82833300
H	-3.51681600	1.25776100	0.74032200
H	-2.70539100	2.02518500	2.11067700
H	-0.74645300	0.42709800	1.67679600
H	-0.78033000	2.82043100	0.97433500
H	0.73924200	1.03925800	-1.02613500
H	2.98700800	0.93120100	-0.97649900
H	4.46759300	2.89810200	0.85022900
H	4.91647100	-0.89798100	0.83353500
H	5.85548600	-3.51603900	-0.44047500
H	3.51687400	-3.63464700	-1.21491100
H	2.94572800	-1.59605200	1.00870700
H	1.09447500	-2.90195200	-1.12511300
H	0.71909000	-0.73259200	1.00792800
H	-2.61132200	2.76982200	-0.72170700
H	-1.63439500	1.73279400	-1.76441000
H	-1.11508900	3.39969700	-1.44147500
H	6.03940300	2.30110000	-1.11894600
H	6.06982900	0.97122600	1.58173900
H	7.18090000	2.09909600	0.82906200
H	8.50477500	0.21910600	0.89529600
H	7.16393100	-1.32428700	1.97509800
H	7.72666800	-2.14397700	0.52334100
H	0.90098600	3.48224100	1.85629100
H	2.40361800	4.10181500	1.16146600
H	2.45576200	2.77080300	2.31407700
H	6.03988900	0.06378500	-1.63688800
H	8.45198700	-0.72417600	-1.23169100

C	-6.01291500	2.87394500	-0.10463300
C	-6.28329900	1.94542400	-1.30306100
C	-5.52051900	0.65412700	-1.23250900
C	-4.45878500	0.26590200	-2.01472300
C	-5.74729900	-0.42862400	-0.30497300
C	-4.78179700	-1.43279700	-0.59636300
C	-6.65711100	-0.64634900	0.74428500
N	-4.03463300	-1.00267400	-1.67040300
C	-4.68742700	-2.61604300	0.14364400
C	-6.57110800	-1.82371300	1.47952900
C	-5.59178200	-2.79641200	1.18541400
H	-4.95931400	3.17527700	-0.07769500
H	-6.23336200	2.36634000	0.84096800
H	-6.62620100	3.78164100	-0.15678000
H	-6.03135100	2.47074900	-2.23330200
H	-7.36180100	1.73689500	-1.35250800
H	-3.97466700	0.80379700	-2.81926400
H	-3.10484000	-1.37326000	-1.85634300
H	-7.41454700	0.09752300	0.97985800
H	-3.92588300	-3.35511000	-0.08880500
H	-7.26715100	-2.00040500	2.29554800
H	-5.54444700	-3.70295400	1.78320600

2

$G_{\text{diethylether}}$ = -1561.089311 Hartree

C	4.17513300	2.05555300	-1.20082800
O	1.68092500	0.48463700	2.75114900
C	2.87388900	2.83980300	-1.01277000
O	2.17276300	1.62157700	0.91416200
C	1.75646400	1.98037200	-0.43474700
O	-5.72670500	1.21175600	0.80012700
C	0.36334400	2.62833200	-0.37507500
O	-7.25573300	-1.13368900	0.45559500
C	-0.66556900	1.63114900	0.24852800
C	-1.73282200	1.10617800	-0.69870000
C	-2.99427900	0.99549500	-0.24512200
C	-4.16296700	0.28495600	-0.87247200
C	-4.25065500	-1.23480900	-0.43998600
C	-4.19519300	-1.50708400	1.05503700
C	-3.07865800	-1.51893100	1.80811200
C	-1.77303900	-1.16793900	1.26052200

C	-0.87210500	-0.34471400	1.81989200
C	0.08114200	0.39424000	0.93525300
C	1.35185100	0.83146700	1.63195600
C	0.36418100	3.95706300	0.39527200
C	-5.48590100	1.04154700	-0.59198700
C	-6.66703500	0.35862900	-1.29982000
C	-6.80578400	-1.11627000	-0.91653100
C	-5.48674800	-1.87975700	-1.11160700
C	-1.25911500	0.53314300	-2.01320200
H	4.03372500	1.20387300	-1.87622300
H	4.53230900	1.65472300	-0.24849900
H	4.95890400	2.69414200	-1.62244800
H	3.04393400	3.69683300	-0.35155600
H	2.52446700	3.24060200	-1.97390900
H	1.69974700	1.05256100	-1.02068300
H	0.07682900	2.83955900	-1.41199200
H	-1.17719600	2.14780500	1.06710000
H	-3.21621400	1.38210000	0.74721100
H	-4.04724200	0.26460400	-1.96587500
H	-3.36969200	-1.70744600	-0.89273000
H	-5.14513600	-1.67529000	1.55246300
H	-3.16581400	-1.65983900	2.88467900
H	-1.61158300	-1.43972200	0.21874000
H	-0.97795100	0.00519400	2.84454400
H	0.41911000	-0.26509000	0.12599000
H	1.00299000	4.70603200	-0.08317900
H	0.72020400	3.81473500	1.42164500
H	-0.65131000	4.36476000	0.44159500
H	-5.37575200	2.05631200	-0.99538800
H	-6.52708800	0.42236500	-2.38643200
H	-7.59466400	0.88513300	-1.05094100
H	-7.57983900	-1.58252500	-1.54556800
H	-5.30897400	-1.94175600	-2.19410300
H	-5.60444500	-2.91528600	-0.76172900
H	-0.54852000	-0.29165700	-1.85575600
H	-0.73279900	1.27898800	-2.62195400
H	-2.08097300	0.14094300	-2.61691900
H	-6.18589400	0.41137900	1.10947000
H	-7.30579900	-2.05715200	0.74812100
C	7.10921000	0.01011000	-1.73843300
C	7.13140800	-0.89794400	-0.49562800
C	5.80783500	-0.98546400	0.20858800
C	5.47751400	-0.45511700	1.43243800

C	4.62087900	-1.65069900	-0.27027200
C	3.62166200	-1.50017000	0.73312400
C	4.30221200	-2.35653100	-1.44381200
N	4.17681300	-0.78113900	1.76920700
C	2.34241800	-2.05325400	0.60080700
C	3.02190100	-2.87768100	-1.59546000
C	2.05238400	-2.72998200	-0.58054900
H	6.87754300	1.04302600	-1.45906700
H	6.34366000	-0.31713400	-2.45057800
H	8.07825000	0.00084100	-2.25158000
H	7.89383300	-0.53423900	0.20507500
H	7.45158800	-1.90553400	-0.79764800
H	6.08804800	0.12487700	2.11195600
H	3.62293200	-0.32926800	2.48870800
H	5.04860300	-2.49228600	-2.22253900
H	1.60885300	-1.96353600	1.39613300
H	2.76561800	-3.42026800	-2.50172700
H	1.06670900	-3.16979100	-0.71226600

3

$G_{diethylether}$ = -1561.079929 Hartree

C	-3.67547200	1.62915900	1.31336800
O	-1.45859900	-0.34464000	-2.32631500
C	-2.53472400	2.58717500	0.96131100
O	-1.65049000	1.19925600	-0.75504300
C	-1.27574000	1.85972700	0.48981300
O	5.30939400	0.54965600	-1.67834200
C	-0.03678200	2.78616100	0.29893700
O	7.39321800	-1.08847200	-0.82501800
C	1.18723800	1.98577800	-0.09310900
C	1.99191300	1.23661500	0.68200400
C	2.95804000	0.28066600	0.00522500
C	4.39159600	0.24556900	0.61002300
C	4.88572900	-1.19278100	0.95697500
C	4.55972800	-2.15330700	-0.16214000
C	3.32408200	-2.15155300	-0.66578700
C	2.30369100	-1.19200500	-0.11040000
C	1.06281300	-1.05481900	-0.93335300
C	-0.08328300	-0.61500300	-0.39746600
C	-1.10835900	0.04859900	-1.22569500
C	-0.29469400	3.90342200	-0.72469800

C	5.36582600	1.00396700	-0.33262800
C	6.80580300	0.97906200	0.20540000
C	7.30370200	-0.44442100	0.46225900
C	6.35833300	-1.19382900	1.40797100
C	1.82323000	1.12778900	2.17825600
H	-3.36842800	0.89788100	2.07116500
H	-4.00475800	1.07549500	0.43291100
H	-4.53828000	2.17682100	1.70666100
H	-2.86240600	3.28002200	0.17837300
H	-2.25894000	3.19594100	1.83316400
H	-1.02390900	1.10282300	1.23880300
H	0.12063400	3.24495300	1.28474900
H	1.36273000	1.94673600	-1.16836300
H	3.07405400	0.59097000	-1.03671600
H	4.40361100	0.80362600	1.55541300
H	4.29478300	-1.51232900	1.83081900
H	5.32369000	-2.83097200	-0.53412700
H	3.03076600	-2.82074700	-1.47133200
H	2.03330700	-1.50084800	0.91177500
H	1.17139100	-1.07133800	-2.01744600
H	-0.17704800	-0.55111900	0.68042700
H	-1.09233700	4.57844400	-0.40128000
H	-0.58226600	3.47510300	-1.69106900
H	0.61223700	4.49927400	-0.87173900
H	5.02288500	2.04595100	-0.37169500
H	6.85985600	1.53687100	1.14931800
H	7.46477200	1.47401500	-0.51542900
H	8.30774200	-0.40429900	0.91244000
H	6.43297400	-0.71820100	2.39530700
H	6.70600300	-2.22898100	1.54033400
H	1.48879700	0.11979200	2.46380500
H	1.09147000	1.83805000	2.57099600
H	2.76569200	1.29943900	2.71200600
H	5.82545900	-0.27530400	-1.71842500
H	7.64611800	-2.01417400	-0.68568700
C	-7.54259600	1.09683500	0.97682800
C	-7.58937300	0.15526100	-0.24088500
C	-6.23403400	-0.35141800	-0.64031500
C	-5.48868000	0.02882700	-1.73038300
C	-5.42735100	-1.30353700	0.08198100
C	-4.20972000	-1.45713500	-0.64097900
C	-5.60593500	-2.02761600	1.27333000
N	-4.28359500	-0.64671500	-1.75282700

C	-3.18566200	-2.30760500	-0.20587600
C	-4.58659500	-2.86224000	1.71699500
C	-3.38862800	-2.99947700	0.98375300
H	-6.94530300	1.98735400	0.75128900
H	-7.07938800	0.59988900	1.83644200
H	-8.54877400	1.42069400	1.26925600
H	-8.05059000	0.67896200	-1.08805800
H	-8.24898500	-0.69352100	-0.01028800
H	-5.73359800	0.73483700	-2.51296300
H	-3.48219400	-0.42151000	-2.33334500
H	-6.52870700	-1.93243800	1.84053300
H	-2.27481100	-2.42431600	-0.78380200
H	-4.71331200	-3.42348400	2.63923800
H	-2.61252400	-3.66695600	1.34980300

4

$G_{diethylether}$ = -1504.750864 Hartree

C	5.06160600	-3.59052000	0.12715600
O	3.08562600	-0.29397000	2.48982500
C	4.50100500	-2.70202900	-0.98690900
O	3.44846100	-1.18696400	0.50111500
C	3.15790500	-2.07006300	-0.61089200
O	-3.26936000	1.12825000	-0.23817700
C	2.47159600	-1.30605600	-1.77706900
O	-5.77470700	0.81195700	0.64549100
C	1.10444400	-0.80087600	-1.39283700
C	-0.10821700	-1.20103500	-1.83973800
C	-1.28168100	-0.46305600	-1.35661400
C	-2.53327400	-0.51651300	-1.84356900
C	-3.82648400	-1.41433100	1.26252800
C	-3.46817600	-1.14708100	2.53170000
C	-2.07943600	-0.93547500	2.92711600
C	-1.03091700	-1.48063500	2.26701400
C	0.35003600	-1.11924900	2.44167300
C	1.29733000	-1.56380000	1.58701800
C	2.65312400	-0.99027300	1.58955600
C	3.32673600	-0.12518700	-2.27921400
C	-3.62540800	0.45543600	-1.45334900
C	-5.07292800	-0.13874600	-1.43226400
C	-5.76537800	-0.43146200	-0.08903600

C	-5.22580800	-1.59519600	0.77046900
C	-0.33595200	-2.30853700	-2.84039700
H	4.37595300	-4.41513100	0.36046600
H	5.21419200	-3.00804700	1.04089700
H	6.02291200	-4.02690000	-0.16453100
H	5.21562200	-1.90498300	-1.21779700
H	4.35958900	-3.28605100	-1.90600200
H	2.49148900	-2.87463400	-0.27950700
H	2.38454300	-2.04255300	-2.58558700
H	1.12335100	0.03332800	-0.69367800
H	-1.08451600	0.23422100	-0.55001000
H	-2.78294100	-1.16757800	-2.68088300
H	-3.05694700	-1.44167500	0.49719900
H	-4.23428600	-1.03759100	3.30142600
H	-1.89099100	-0.26951300	3.76941900
H	-1.23667500	-2.19358300	1.46989900
H	0.62398000	-0.40183100	3.21282600
H	1.00398100	-2.24252800	0.79765700
H	4.28715900	-0.46396800	-2.68093300
H	3.51660200	0.57950300	-1.46548900
H	2.79566300	0.41307400	-3.07019400
H	-3.63479200	1.21085300	-2.25718100
H	-5.09506000	-1.05651200	-2.03153700
H	-5.72940800	0.57681200	-1.93950000
H	-6.80689900	-0.69034700	-0.33511200
H	-5.29898400	-2.50723200	0.16044700
H	-5.91702000	-1.73193200	1.61574800
H	0.57867700	-2.85039300	-3.09035600
H	-0.75302300	-1.90995000	-3.77468500
H	-1.06303600	-3.03453600	-2.45436900
H	-4.09413500	1.29051600	0.26505200
H	-5.95676200	0.61906000	1.57879800
C	4.16667500	3.63214800	1.73807500
C	3.82481200	2.51584300	0.73591100
C	2.40189100	2.61335900	0.22739200
C	2.10928700	3.18089800	-1.02114900
C	1.32226900	2.17592700	1.00747300
C	0.80164200	3.30215900	-1.48345400
C	0.00369600	2.29590600	0.56329600
C	-0.26670900	2.85770400	-0.69478300
O	-1.52327800	2.99159700	-1.19767200
H	5.20073700	3.54226700	2.09187100
H	3.50557100	3.58109700	2.61081300

H	4.04244300	4.62224300	1.28340100
H	3.98219000	1.54425200	1.21642600
H	4.51894900	2.56864800	-0.11363900
H	2.92714800	3.52675900	-1.65055000
H	1.52283000	1.71886900	1.97293600
H	0.58391200	3.72759500	-2.45847200
H	-0.81892900	1.93646200	1.17759400
H	-2.15913800	2.44939400	-0.67814000

5

$G_{\text{diethylether}}$ = -1504.765793 Hartree

C	5.65780300	-3.19326100	-0.55867200
O	3.02852100	-0.28919900	2.39395400
C	4.60679900	-2.41641500	-1.35725200
O	3.70441700	-1.30620800	0.54377300
C	3.32980900	-2.16727400	-0.56189100
O	-3.38693300	1.47827500	-0.34610700
C	2.16150900	-1.54898500	-1.35092800
O	-5.82831500	0.69233800	0.69576600
C	1.02164600	-1.08875100	-0.38639700
C	-0.37375200	-1.53099600	-0.79113300
C	-1.38664500	-0.66625000	-0.61399800
C	-2.86771300	-0.90367500	-0.73039300
C	-3.52699600	-1.44405400	0.60136300
C	-3.23817100	-0.64476300	1.86239500
C	-2.08721100	-0.66708400	2.55899300
C	-0.91655400	-1.42446000	2.12424700
C	0.32815500	-0.93005400	2.06807500
C	1.34683300	-1.50140900	1.13247200
C	2.74636100	-0.98686400	1.44543500
C	2.58513400	-0.37858300	-2.24838500
C	-3.58149200	0.36990200	-1.23860900
C	-5.07406100	0.11196400	-1.47801200
C	-5.77394900	-0.42119800	-0.22558500
C	-5.04425800	-1.63912000	0.36361900
C	-0.56813300	-2.97773200	-1.17839300
H	5.28239700	-4.18208800	-0.26663100
H	5.92316700	-2.65033200	0.35346600
H	6.56881000	-3.34114000	-1.14834800
H	5.01710300	-1.45112100	-1.67300100
H	4.33663900	-2.96404600	-2.27001600

H	2.99852300	-3.12714000	-0.13853300
H	1.79217000	-2.35720800	-1.99318500
H	1.02817500	0.00451100	-0.36987800
H	-1.13146100	0.32805000	-0.26636500
H	-3.06207600	-1.67961700	-1.48527800
H	-3.10059500	-2.44565800	0.74273000
H	-4.02427400	0.02010400	2.20660000
H	-1.97322600	0.00374100	3.41004900
H	-1.11453000	-2.37570900	1.63294100
H	0.56198500	0.03478200	2.50753800
H	1.35218800	-2.59890500	1.18596800
H	3.24695300	-0.70070400	-3.05909100
H	3.09674400	0.39094300	-1.66213600
H	1.70207300	0.09005900	-2.69455900
H	-3.10679900	0.67689400	-2.17687900
H	-5.18653500	-0.62342900	-2.28386300
H	-5.56332300	1.03735000	-1.80026900
H	-6.80368500	-0.71338800	-0.48063900
H	-5.18992500	-2.46940000	-0.34095600
H	-5.53408600	-1.94335200	1.29948700
H	-0.03362000	-3.64439100	-0.48696800
H	-0.17855800	-3.19479100	-2.18155800
H	-1.62048600	-3.27399200	-1.16816700
H	-4.10235600	1.46452800	0.31658600
H	-6.20422000	0.38091600	1.53391300
C	4.17372800	3.92661200	1.46119600
C	3.78742800	2.62477500	0.73744200
C	2.39682400	2.68542300	0.14223500
C	2.19078600	3.01748700	-1.20454600
C	1.26140100	2.45115500	0.92924400
C	0.91378400	3.09259000	-1.75421600
C	-0.02835200	2.53121700	0.39949700
C	-0.21107300	2.84349100	-0.95706200
O	-1.43113700	2.91540900	-1.55179800
H	5.18253600	3.85868800	1.88561000
H	3.47497900	4.13628800	2.27946000
H	4.14587100	4.78127400	0.77486000
H	3.85371300	1.78478400	1.43800700
H	4.51651900	2.42521400	-0.05922800
H	3.05261200	3.20818100	-1.84136800
H	1.39254400	2.19026700	1.97662200
H	0.76313800	3.33450200	-2.80203900
H	-0.89506600	2.32969000	1.02457900

H	-2.13426200	2.58861000	-0.94860700

6

$G_{diethylether}$ = -1504.758789 Hartree

C	5.03303200	-3.57736800	-0.24428400
O	2.98837100	-0.28858300	2.40283800
C	4.34481300	-2.62537500	-1.22659700
O	3.37105300	-1.27262300	0.45521600
C	3.01432000	-2.09267000	-0.69230600
O	-3.05021700	1.46273700	0.12925200
C	2.17817900	-1.30432300	-1.74581800
O	-5.66685600	0.85450600	0.63679700
C	0.88583300	-0.80921200	-1.13537800
C	-0.26563000	-1.48070100	-0.94970600
C	-1.30624100	-0.87207800	-0.02617400
C	-2.76854400	-0.90179900	-0.55676200
C	-3.77863100	-1.58168200	0.41663800
C	-3.56427900	-1.09336900	1.83065900
C	-2.32264200	-1.04844800	2.31684500
C	-1.17012500	-1.49485800	1.45552700
C	0.17963500	-1.08949100	1.95590200
C	1.28576600	-1.75516300	1.60015000
C	2.59618000	-1.07101400	1.56226100
C	2.96628100	-0.13992200	-2.36592100
C	-3.20559200	0.52827600	-0.95111400
C	-4.64121100	0.56362800	-1.49045400
C	-5.63779300	-0.03909500	-0.49837300
C	-5.22655100	-1.46079500	-0.09715000
C	-0.48501300	-2.90022500	-1.41485400
H	4.40592900	-4.45350600	-0.03509500
H	5.23245100	-3.06926500	0.70415400
H	5.98625200	-3.93677600	-0.64656500
H	5.00279400	-1.77683800	-1.44216400
H	4.15170500	-3.13441700	-2.18028400
H	2.41573000	-2.94753200	-0.35964400
H	1.96285100	-2.04167300	-2.53194200
H	0.95526300	0.19147300	-0.71261700
H	-1.04546100	0.17804200	0.11275700
H	-2.80338800	-1.48025700	-1.48956300
H	-3.53423100	-2.65620400	0.40755900
H	-4.41844200	-0.79286700	2.43264300

H	-2.11679900	-0.70460300	3.32806100
H	-1.21107900	-2.59012200	1.34751700
H	0.27489100	-0.09420100	2.38590100
H	1.18721700	-2.72081700	1.11732000
H	3.81862100	-0.49366100	-2.95436200
H	3.33481400	0.52774100	-1.58263200
H	2.31826600	0.44905900	-3.02242700
H	-2.51816200	0.88697200	-1.72460100
H	-4.69427600	-0.00549200	-2.42674400
H	-4.92123300	1.59862600	-1.71298300
H	-6.63936200	-0.06584500	-0.95319000
H	-5.34904700	-2.09910100	-0.98253700
H	-5.92429900	-1.85035600	0.65866800
H	-0.45603900	-3.60410200	-0.56969900
H	0.27064000	-3.22576800	-2.13442500
H	-1.46609500	-3.03190300	-1.88758700
H	-3.84762600	1.40160400	0.68958500
H	-6.21158200	0.45385000	1.33167600
C	4.43241200	3.54290100	1.54219700
C	3.95460800	2.42485200	0.59948700
C	2.52127200	2.61855800	0.15174100
C	2.21123000	3.13232200	-1.11526100
C	1.45204200	2.31881000	1.00729600
C	0.89480100	3.32495200	-1.52717300
C	0.12607100	2.51606900	0.61654100
C	-0.16267800	3.01466900	-0.66370800
O	-1.43054000	3.21940600	-1.11594800
H	5.47106600	3.37964600	1.85371900
H	3.81108400	3.57775700	2.44444800
H	4.36851500	4.52359700	1.05579300
H	4.05219800	1.45948200	1.10784800
H	4.60964300	2.39085500	-0.28151300
H	3.02056800	3.37434100	-1.80145500
H	1.66677200	1.90839900	1.99072000
H	0.66259900	3.71006000	-2.51561900
H	-0.69204500	2.27288300	1.28954900
H	-2.07448200	2.80270100	-0.50076400

7

$G_{\text{diethylether}}$ = -1675.308488 Hartree

C	5.32994000	0.65430900	-0.67365600

O	2.67764900	0.50174300	3.24917600
C	4.39667100	1.86715500	-0.72290200
O	3.23336600	1.31547900	1.26338300
C	3.00995600	1.56579400	-0.14596100
O	-4.27090000	1.58346800	0.95975700
C	1.98671800	2.71611900	-0.36759900
O	-6.01971700	-0.54741700	0.87346500
C	0.56264000	2.35031000	-0.00873400
C	-0.41357900	1.88720700	-0.82366200
C	-1.77537700	1.77182300	-0.28970700
C	-2.88566500	1.49477400	-0.99852300
C	-3.40075100	-1.59231100	-0.04565600
C	-3.17731500	-2.01045600	1.21795000
C	-1.97064500	-1.70387500	1.97261800
C	-0.80590900	-1.30229100	1.41009700
C	0.32744600	-0.76114800	2.10870200
C	1.34004600	-0.19677500	1.41354600
C	2.43724400	0.53654800	2.05907300
C	2.38778900	3.99581600	0.38981600
C	-4.29089500	1.65976900	-0.47310100
C	-5.38896400	0.76118800	-1.10849300
C	-5.70865900	-0.62991800	-0.51156700
C	-4.69124500	-1.77412000	-0.77903900
C	-0.19234300	1.52725500	-2.27382300
H	4.92727200	-0.17743300	-1.26446600
H	5.45279800	0.30686000	0.35677600
H	6.31981600	0.90268600	-1.07175300
H	4.84338500	2.69797800	-0.16611300
H	4.27064100	2.20333100	-1.76056600
H	2.63335700	0.66292400	-0.63840600
H	2.03216400	2.92605400	-1.44385100
H	0.29252600	2.54409100	1.02939800
H	-1.89589000	1.99763700	0.76777600
H	-2.82529300	1.28088600	-2.06430200
H	-2.64982700	-0.98221400	-0.53830300
H	-3.96320000	-2.55914800	1.73650900
H	-2.04042700	-1.75472700	3.05926200
H	-0.72970200	-1.29856400	0.32349000
H	0.33242600	-0.72613500	3.19681800
H	1.29275100	-0.22312800	0.33502400
H	3.35916100	4.37528100	0.05838900
H	2.45091100	3.79923700	1.46491300
H	1.64466600	4.78318200	0.22572100

H	-4.57473300	2.69021400	-0.75818100
H	-5.18075400	0.66064900	-2.18256700
H	-6.32929100	1.32265000	-1.03342900
H	-6.64314400	-0.94662200	-0.99174500
H	-4.51624900	-1.84569500	-1.86072900
H	-5.18351000	-2.69961300	-0.45840200
H	0.85347800	1.61571800	-2.57349200
H	-0.79362200	2.16279800	-2.93710400
H	-0.49329300	0.48870400	-2.45791100
H	-5.14083900	1.88245600	1.26996500
H	-5.19462800	-0.26147600	1.30517600
C	3.69253100	-3.37290400	1.39801200
C	3.44896800	-2.59090600	0.10279400
C	2.10374600	-2.95610400	-0.53059000
S	1.57414400	-1.88449300	-1.92847400
C	2.90983000	-2.20583200	-3.13486200
H	4.67268900	-3.13120500	1.82303400
H	3.65647800	-4.45579800	1.22634500
H	2.93272500	-3.12172100	2.14702900
H	4.26017100	-2.78551100	-0.61016100
H	3.47292500	-1.51769200	0.31601700
H	1.30132900	-2.85217600	0.20725300
H	2.10244100	-4.00009300	-0.86762700
H	3.87192200	-1.81856400	-2.78775900
H	2.99875600	-3.27739300	-3.34085800
H	2.63703700	-1.69127800	-4.06022500

8

$G_{diethylether}$ = -1675.318010 Hartree

C	-5.23952900	-1.81608500	-1.50168800
O	-2.85356800	-0.35782700	2.56260800
C	-3.99611700	-2.67266500	-1.24004800
O	-3.33795100	-1.43917000	0.68456900
C	-2.85892800	-1.87000700	-0.61749700
O	4.57308200	-1.57942200	1.01788800
C	-1.51792600	-2.60282300	-0.45063000
O	6.27454400	0.65342800	0.73805500
C	-0.46792500	-1.64765600	0.20330800
C	0.68101500	-1.21632100	-0.69317000
C	1.92113100	-1.18664900	-0.17298800
C	3.16731500	-0.56651500	-0.74390100

C	3.33611000	0.94984600	-0.32243700
C	3.21632600	1.24633900	1.16422900
C	2.06120200	1.34829800	1.84945200
C	0.77011700	1.08257200	1.22603100
C	-0.22433100	0.34395700	1.74441200
C	-1.17428700	-0.34764800	0.81757300
C	-2.49873500	-0.70413000	1.45758500
C	-1.65166200	-3.89916800	0.36223200
C	4.41802900	-1.40844700	-0.38639200
C	5.68000300	-0.81682800	-1.03445400
C	5.89928300	0.64964300	-0.65658700
C	4.64879500	1.49910600	-0.93048400
C	0.31819500	-0.62539400	-2.03497600
H	-5.01428600	-0.99273100	-2.19099300
H	-5.60708800	-1.38069200	-0.56746400
H	-6.04399200	-2.41400000	-1.94301000
H	-4.25194200	-3.50437200	-0.57475300
H	-3.62835600	-3.11026500	-2.17769300
H	-2.69567200	-0.97395900	-1.23437000
H	-1.18161800	-2.86946700	-1.45979900
H	-0.03590900	-2.16918600	1.06358000
H	2.06229700	-1.57125800	0.83473100
H	3.11009100	-0.55229500	-1.84216700
H	2.51674900	1.47573700	-0.82821800
H	4.14580700	1.35282400	1.71476600
H	2.09451800	1.49826100	2.92789500
H	0.69202100	1.34594500	0.17443400
H	-0.21068300	0.01214400	2.78047500
H	-1.41550100	0.31062400	-0.02679000
H	-2.30779800	-4.62441300	-0.12964000
H	-2.05830200	-3.69681900	1.35921300
H	-0.66931100	-4.36770400	0.48469700
H	4.25975800	-2.41801300	-0.78694500
H	5.59487300	-0.88375900	-2.12659500
H	6.55426600	-1.40268800	-0.73080800
H	6.73693200	1.05405800	-1.24595700
H	4.53501700	1.55927000	-2.02172800
H	4.81869400	2.52864900	-0.58423200
H	-0.30499500	-1.30574200	-2.62891100
H	1.19971300	-0.38482300	-2.63456900
H	-0.25928900	0.30343000	-1.91597700
H	5.06280400	-0.80490900	1.34570900
H	6.35915000	1.57537400	1.02737000

C	-5.32410700	1.51460500	1.05275500
C	-4.28119200	1.83842800	-0.02357600
C	-3.12079300	2.66871000	0.53357400
S	-1.75503600	2.99855700	-0.65526000
C	-2.64066000	3.96442900	-1.93075300
H	-6.15357200	0.93917500	0.62574100
H	-5.74154700	2.43132800	1.48763100
H	-4.88068000	0.92151900	1.85860500
H	-4.75833100	2.37874400	-0.85102700
H	-3.89006800	0.90626600	-0.44604700
H	-2.64191500	2.14636900	1.36901100
H	-3.48415800	3.62990800	0.91792500
H	-3.37219000	3.35816400	-2.47206000
H	-3.14234500	4.82869100	-1.48294600
H	-1.88940800	4.32405400	-2.63930100

9

$G_{diethylether}$ = -1675.308914 Hartree

C	5.03441900	1.44658600	-0.91454600
O	2.40710200	0.75653200	2.96488000
C	3.87610900	2.44531100	-0.98901700
O	2.81219400	1.67586700	0.98520700
C	2.56782300	1.87602400	-0.43571500
O	-4.26014000	1.17795000	1.28455100
C	1.33041000	2.78868900	-0.69813100
O	-6.24219100	-0.71107900	0.75277500
C	0.05387200	2.14015400	-0.20735700
C	-0.66313900	1.15431400	-0.77729000
C	-1.71863500	0.45778800	0.06218600
C	-3.07029100	0.18824000	-0.66019600
C	-3.53122500	-1.29986300	-0.58336300
C	-3.35930300	-1.83907400	0.81711000
C	-2.19840500	-1.63464200	1.44242500
C	-1.10463100	-0.87551300	0.73819200
C	0.02521800	-0.44413800	1.62002300
C	1.22168100	-0.14601800	1.09769800
C	2.18031600	0.75171900	1.77444700
C	1.49292300	4.18472000	-0.07566000
C	-4.14505300	1.17705300	-0.13235600
C	-5.50796900	0.93890500	-0.80316300
C	-5.98436300	-0.50689000	-0.65148300

C	-4.93590400	-1.48954000	-1.18558300
C	-0.30273200	0.54751500	-2.11218100
H	4.79499100	0.52664200	-1.46191100
H	5.24146100	1.17668800	0.12532100
H	5.94781300	1.87107900	-1.34552700
H	4.13558100	3.35300900	-0.43352300
H	3.70116100	2.74342400	-2.03134800
H	2.39233400	0.90945400	-0.91887100
H	1.29184000	2.89385800	-1.79155400
H	-0.25180300	2.46200400	0.78823300
H	-1.95108000	1.09523400	0.91951700
H	-2.96397000	0.41591000	-1.72902000
H	-2.83834900	-1.86427400	-1.22848000
H	-4.17102700	-2.38529000	1.29063700
H	-2.01838800	-1.99823600	2.45163900
H	-0.71033600	-1.49148800	-0.08562300
H	-0.20874600	-0.13938900	2.63966400
H	1.42222100	-0.40866300	0.06708800
H	2.33173900	4.73400400	-0.51348100
H	1.66712000	4.10135800	1.00258400
H	0.58518100	4.77748300	-0.23074000
H	-3.79945700	2.18707900	-0.38775200
H	-5.43944700	1.16644200	-1.87492900
H	-6.24640700	1.61664100	-0.36235800
H	-6.92358700	-0.64222800	-1.21027900
H	-4.88189200	-1.35573800	-2.27455800
H	-5.27529200	-2.52296500	-1.02111900
H	0.38290500	1.17843600	-2.68405100
H	-1.18599800	0.37049100	-2.73676700
H	0.18918600	-0.42758800	-1.98464000
H	-4.77920900	0.38982300	1.52458600
H	-6.47659300	-1.64249500	0.88615100
C	4.27830500	-2.15438300	2.33725100
C	4.20993200	-1.91648800	0.82430600
C	3.13431700	-2.78111200	0.15961900
S	2.71339200	-2.31538100	-1.57010500
C	4.32794900	-2.55667400	-2.39368400
H	5.09955900	-1.58358000	2.78362300
H	4.43854300	-3.21490200	2.56841200
H	3.35257600	-1.82750300	2.82249600
H	5.18465300	-2.12652900	0.36581800
H	4.00042400	-0.86016900	0.62870900
H	2.18773000	-2.68464500	0.70207500

H	3.41664200	-3.84087700	0.18186500
H	5.07497800	-1.84201800	-2.03752300
H	4.69355400	-3.57705400	-2.23943300
H	4.16964200	-2.39758300	-3.46375000

10

$G_{diethylether}$ = -1504.731823 Hartree

C	-3.78290400	3.08972200	-2.01448000
O	-0.52687000	4.60788300	1.12307400
C	-3.61808800	1.99768700	-0.95408900
O	-1.89052200	3.12159800	0.20950200
C	-2.17411000	1.87299100	-0.46111900
O	4.13915600	-1.05940400	1.99094500
C	-1.93736500	0.65795100	0.47657500
O	6.63173300	-0.92085100	0.82899500
C	-0.47750300	0.39063700	0.77857600
C	0.37905700	-0.40744400	0.10085700
C	1.68951500	-0.69432600	0.69290800
C	2.56718500	-1.62119300	0.26594900
C	4.76970100	0.29528300	-1.14435400
C	5.08475000	1.48887000	-0.59941900
C	4.09611500	2.46554200	-0.16689000
C	2.81910200	2.50769700	-0.61629200
C	1.75635400	3.27408000	-0.02819500
C	0.46950800	3.06744300	-0.38511000
C	-0.64521000	3.66638100	0.36508800
C	-2.70760500	0.80136300	1.80081500
C	3.76240900	-2.08662700	1.06086600
C	4.97177900	-2.63867100	0.25568500
C	6.08670100	-1.68634900	-0.23755000
C	5.76345400	-0.77841000	-1.45699900
C	0.03526900	-1.07085100	-1.21143000
H	-3.16742600	2.87911900	-2.89849000
H	-3.47802100	4.06140100	-1.61445500
H	-4.82523900	3.16591300	-2.34303200
H	-4.26699800	2.21520300	-0.09856800
H	-3.92466000	1.02370800	-1.35477200
H	-1.53325900	1.76117700	-1.34328700
H	-2.34690600	-0.20537500	-0.06053600
H	-0.10788000	0.84991600	1.69549500
H	1.93183900	-0.15240000	1.60457300

H	2.36181300	-2.21118500	-0.62574800
H	3.72241600	0.03364400	-1.26069700
H	6.13229100	1.71791800	-0.40494900
H	4.40214300	3.16702000	0.60947000
H	2.53174400	1.84377800	-1.43059100
H	1.97594800	3.95734300	0.79044900
H	0.25997100	2.36107600	-1.17778700
H	-3.78538400	0.87668800	1.63321800
H	-2.38338800	1.69679900	2.34072100
H	-2.52949700	-0.07351800	2.43397700
H	3.39291800	-2.95392400	1.63927000
H	4.58892000	-3.22603200	-0.59025600
H	5.48110200	-3.35353700	0.91508800
H	6.90643500	-2.33867500	-0.56452200
H	5.40739000	-1.40919700	-2.28241300
H	6.71758600	-0.33500100	-1.76479000
H	-0.94446200	-0.76798000	-1.58670800
H	0.02795300	-2.16265800	-1.11515300
H	0.78319000	-0.81365600	-1.97353600
H	4.76164800	-1.46080400	2.61845100
H	5.89579100	-0.37023000	1.15229500
C	-6.43028100	-0.80188100	1.99011600
C	-6.05809200	-0.72678600	0.50703500
C	-4.89145600	-1.59586600	0.06811900
C	-4.07210100	-2.29383300	0.96477600
C	-4.59225700	-1.70482200	-1.29738600
C	-2.99927000	-3.06927200	0.52550300
C	-3.52480900	-2.47545900	-1.75560000
C	-2.72774800	-3.17009700	-0.83942700
O	-1.67203900	-3.95901100	-1.22065700
H	-6.67593000	-1.82736300	2.28911500
H	-7.30275100	-0.17299900	2.19532800
H	-5.61282000	-0.45113600	2.62985400
H	-5.83338200	0.31800800	0.24900600
H	-6.93703100	-0.99246100	-0.09646400
H	-4.26246400	-2.23504500	2.03121400
H	-5.21029600	-1.17756200	-2.02136300
H	-2.36753900	-3.60401800	1.22746900
H	-3.31842500	-2.54647500	-2.82225200
H	-1.59161500	-3.93174900	-2.18667000

11

$G_{diethylether}$ = -1504.748533 Hartree

C	5.25067200	2.17309200	1.82863700
O	1.89179500	4.50977200	-1.05438800
C	4.53540300	1.25360300	0.83450600
O	3.09370400	2.95491900	-0.01384800
C	3.07784900	1.64534200	0.61352900
O	-3.61688500	-0.79170400	-1.95922900
C	2.25371600	0.67855900	-0.24929500
O	-6.14855800	-0.30970400	-0.81226200
C	0.78772700	1.19582200	-0.36871300
C	-0.25837700	0.38123800	0.36715100
C	-1.41182200	0.10364500	-0.26427500
C	-2.68672800	-0.44787500	0.30637200
C	-3.65896400	0.69839300	0.80066000
C	-3.91819200	1.82543800	-0.18691600
C	-3.08653000	2.85685900	-0.43411600
C	-1.76343000	2.95282600	0.17354000
C	-0.62063300	3.28005100	-0.45139900
C	0.67502500	2.73633100	0.06637100
C	1.90475400	3.49598300	-0.39735200
C	2.86313600	0.47364900	-1.64408700
C	-3.36947000	-1.40676500	-0.69858100
C	-4.64706400	-2.01159800	-0.09673100
C	-5.63299500	-0.94263800	0.37919700
C	-4.96509500	0.06059200	1.33150100
C	-0.02063200	0.12123400	1.83437500
H	4.77026200	2.13951900	2.81457000
H	5.22503100	3.20865300	1.47612700
H	6.29803600	1.87858800	1.95527300
H	5.05770400	1.27183600	-0.12819700
H	4.55122100	0.21451800	1.19113500
H	2.59681100	1.74056800	1.59884000
H	2.25278700	-0.28992300	0.26155500
H	0.51917500	1.18802400	-1.42979500
H	-1.50220700	0.38842200	-1.31006700
H	-2.46684300	-1.05193100	1.19571500
H	-3.15476200	1.13738700	1.67100600
H	-4.83859300	1.76609200	-0.75977900
H	-3.34662500	3.57125200	-1.21440000
H	-1.67447300	2.52085700	1.16867400
H	-0.61628600	3.68395700	-1.46155200

H	0.68753500	2.76438300	1.16439000
H	3.86092100	0.02760700	-1.59246600
H	2.94398200	1.42275100	-2.18500200
H	2.22913900	-0.20123000	-2.22721700
H	-2.66183400	-2.22000100	-0.89893000
H	-4.37857200	-2.64402000	0.75899400
H	-5.13581300	-2.64774900	-0.84255100
H	-6.46830300	-1.42862000	0.90741900
H	-4.74094600	-0.48133500	2.26087300
H	-5.68351900	0.84763700	1.60261400
H	0.96492100	-0.32351400	2.01110600
H	-0.76464200	-0.56015700	2.25292800
H	-0.06077600	1.05233800	2.41890600
H	-4.46759700	-0.32525200	-1.88372000
H	-6.73031700	0.41781200	-0.54196500
C	4.76273800	-2.95575800	-2.17641900
C	4.53605300	-2.79323200	-0.67082300
C	3.09620900	-2.91099400	-0.19867000
C	1.99777800	-2.88333700	-1.06900400
C	2.82994400	-3.02494900	1.17263300
C	0.68717400	-2.95126600	-0.59844500
C	1.52634900	-3.10365200	1.66086400
C	0.44827300	-3.06631400	0.77103900
O	-0.85825200	-3.13189700	1.18861900
H	4.37583400	-3.91613400	-2.53578700
H	5.83230700	-2.91411800	-2.40739100
H	4.27144200	-2.16005400	-2.74702900
H	4.93103100	-1.81537800	-0.35699800
H	5.14136300	-3.53687700	-0.13474400
H	2.15638700	-2.79890600	-2.13895800
H	3.65918900	-3.05464700	1.87667900
H	-0.15663600	-2.90814600	-1.27890700
H	1.34692900	-3.19427800	2.73097300
H	-0.87490200	-3.22668500	2.15325400

12

$G_{\text{diethylether}}$ = -1504.743296 Hartree

C	5.48317300	-2.12881800	-1.54629500
O	2.30606300	-4.62225000	0.94257000
C	4.88745800	-1.17638700	-0.50586400
O	3.33401600	-2.74849900	0.35010200

C	3.39934700	-1.43325500	-0.26068400
O	-3.27893300	-0.97116300	2.04001200
C	2.70683300	-0.34479900	0.61257600
O	-5.75523300	-0.65431100	0.79016900
C	1.23452300	-0.64449600	0.76801300
C	0.25115900	-0.43125800	-0.12282500
C	-1.09254700	-1.09121500	0.10682700
C	-2.31673400	-0.15876000	-0.10481500
C	-3.34803800	-0.71735700	-1.12972400
C	-3.62247100	-2.17958800	-0.86387800
C	-2.58802000	-3.00355200	-0.67996200
C	-1.18697500	-2.45368900	-0.75150100
C	-0.11282700	-3.35194000	-0.22194000
C	1.15945000	-3.22944300	-0.62417800
C	2.28188500	-3.62607500	0.25592100
C	3.37497800	-0.17980700	1.98555000
C	-2.95937100	0.17812900	1.26611800
C	-4.17320800	1.10586400	1.09861400
C	-5.21670800	0.51965800	0.14653400
C	-4.59329300	0.18460400	-1.21350900
C	0.49068800	0.23772600	-1.45516400
H	4.98332400	-2.01801700	-2.51711000
H	5.36704700	-3.16755400	-1.22237800
H	6.55069700	-1.93371800	-1.69520300
H	5.42587100	-1.28197900	0.44222800
H	5.00549800	-0.13347600	-0.83003900
H	2.89546000	-1.44932200	-1.23278800
H	2.82828000	0.58838200	0.04631000
H	0.97951400	-1.19392300	1.67432600
H	-1.13833600	-1.43478300	1.14411700
H	-1.97877700	0.80257000	-0.50755100
H	-2.85683200	-0.65966900	-2.11529500
H	-4.64742800	-2.53989300	-0.82601300
H	-2.72684200	-4.06361100	-0.48007600
H	-0.96005900	-2.18628000	-1.79565400
H	-0.32968700	-3.91288000	0.68686800
H	1.37786000	-2.62757300	-1.49951800
H	4.40758100	0.17285400	1.90029600
H	3.38437000	-1.13440200	2.52265400
H	2.82096000	0.54597500	2.59125100
H	-2.19395500	0.70311100	1.85221300
H	-3.84244300	2.07034800	0.69480700
H	-4.62996100	1.28015500	2.07861400

H	-6.02845900	1.24955600	0.00039300
H	-4.31023300	1.13500800	-1.68589900
H	-5.34827000	-0.27616800	-1.86807500
H	1.31386000	0.95501800	-1.41502800
H	-0.39193100	0.78244500	-1.80268200
H	0.72901000	-0.50270900	-2.23336600
H	-4.11041900	-1.32767300	1.68013900
H	-6.36404900	-1.08692400	0.17171700
C	3.87377900	4.78144900	0.94955900
C	3.42701100	3.42512900	0.37619500
C	1.95273500	3.40021000	0.03289600
C	0.99876800	2.96362200	0.96249100
C	1.49069500	3.86206900	-1.20608600
C	-0.36284000	2.97986100	0.67428700
C	0.13018500	3.88118300	-1.51469500
C	-0.79933800	3.43447700	-0.57152700
O	-2.15148700	3.39517600	-0.82132500
H	3.68843000	5.58814900	0.23112900
H	4.94306300	4.77616600	1.19057600
H	3.31750100	5.01739600	1.86376500
H	3.64968000	2.63503000	1.10370200
H	4.01977000	3.19798700	-0.51986700
H	1.32747100	2.58870000	1.92844100
H	2.20619600	4.20822500	-1.94888500
H	-1.09381900	2.63308800	1.39639400
H	-0.20574500	4.23042300	-2.48965800
H	-2.32138700	3.75708900	-1.70470300

13

$G_{\text{diethylether}}$ = -1504.743296 Hartree

C	-7.38324400	0.22296400	-0.59482100
O	-4.33807900	1.35877900	2.75926400
C	-6.45847900	-0.99093300	-0.46700900
O	-5.05840000	0.01462800	1.15578400
C	-5.01280300	-0.59305500	-0.15498600
O	2.34906500	-0.55789600	0.40865400
C	-4.01267900	-1.78225700	-0.19400300
O	4.14692400	1.29533900	-0.42912100
C	-2.56821200	-1.35526700	-0.06617600
C	-1.63212300	-1.24716400	-1.03732000
C	-0.24239400	-0.98173600	-0.64571200

C	0.83723400	-1.03739500	-1.44675900
C	1.47534500	2.19849500	-1.33498600
C	1.33066900	3.01457900	-0.27331800
C	0.17649200	2.97055100	0.61625300
C	-1.04055900	2.51583400	0.23724400
C	-2.13094200	2.20596700	1.12231900
C	-3.21701000	1.53945700	0.67447100
C	-4.22448900	1.00017100	1.60535900
C	-4.32596200	-2.83190800	0.89027800
C	2.26491200	-1.03059000	-0.93149900
C	3.33443000	-0.41525100	-1.88186800
C	3.72017100	1.07169500	-1.78496100
C	2.68362000	2.13691400	-2.21434700
C	-1.91943600	-1.44401600	-2.50793100
H	-7.05623300	0.88720700	-1.40495600
H	-7.38778400	0.79963900	0.33499300
H	-8.41121800	-0.08728300	-0.81062200
H	-6.82511900	-1.65152900	0.32584900
H	-6.46137700	-1.57130400	-1.39920600
H	-4.70404500	0.14568900	-0.90354600
H	-4.16414400	-2.24767400	-1.17573800
H	-2.24158800	-1.17623200	0.95803300
H	-0.06565600	-0.78400100	0.40985500
H	0.73303100	-1.26536200	-2.50770200
H	0.71368400	1.44858800	-1.52464700
H	2.12807300	3.71571400	-0.02085800
H	0.33145200	3.27543800	1.65112000
H	-1.19837000	2.27392700	-0.81298200
H	-2.03866700	2.41797800	2.18600200
H	-3.27924000	1.30188600	-0.37929300
H	-5.32107000	-3.26713400	0.75630600
H	-4.28564100	-2.37724000	1.88518000
H	-3.59388200	-3.64494600	0.85253200
H	2.53903200	-2.09780900	-0.88205700
H	3.04654200	-0.62478700	-2.91969000
H	4.26719900	-0.95958200	-1.70160700
H	4.58641200	1.19864900	-2.45363200
H	2.39858300	1.93028700	-3.25461100
H	3.20233100	3.10716300	-2.21861400
H	-2.98253700	-1.57698100	-2.71849200
H	-1.38634600	-2.31951200	-2.90077300
H	-1.57236700	-0.57744800	-3.08525300
H	2.75536600	0.32799700	0.38872300

H	4.35559400	2.23592400	-0.31723400
N	4.82558500	-1.74381300	1.79670800
C	5.41785000	-0.50689700	2.35144000
C	6.82467800	-0.39920300	1.72985700
C	6.63553000	-1.05610400	0.35459900
C	5.69222200	-2.22147100	0.70242900
H	4.82049000	0.37501500	2.07427400
H	3.89855500	-1.53298300	1.43633600
H	5.46038000	-0.54319900	3.44805300
H	7.18431600	0.63423100	1.67716600
H	7.54772000	-0.97316800	2.32367700
H	6.13522800	-0.34912300	-0.31753900
H	7.57403200	-1.37873900	-0.11078400
H	5.09050900	-2.56123500	-0.15055200
H	6.28264600	-3.08845100	1.03734600

14

$G_{\text{diethylether}}$ = -1331.256791 Hartree

C	7.52062600	0.20225600	-0.98161200
O	4.37125600	0.63017900	2.84519300
C	6.24804000	0.97725600	-1.33689100
O	5.24106400	0.69677000	0.80160100
C	5.01692900	0.44640700	-0.60913500
O	-2.58874500	0.57800900	-0.13505200
C	3.67114900	1.06102300	-1.03056700
O	-4.30069500	-1.57707900	0.35226100
C	2.51107100	0.45579000	-0.17472100
C	1.48862400	-0.36713300	-0.94015600
C	0.18662500	-0.20838700	-0.64213300
C	-0.98562800	-1.05605700	-1.05666600
C	-1.26877900	-2.23877500	-0.04283100
C	-1.36810900	-1.85257000	1.42502200
C	-0.32583300	-1.59854900	2.23926000
C	1.05160600	-1.58336600	1.75839700
C	1.97284400	-0.64780100	2.03515700
C	3.08216900	-0.39120300	1.06395700
C	4.26238000	0.34481300	1.67658300
C	3.66533600	2.59405800	-0.93391800
C	-2.24182700	-0.18370100	-1.29331700
C	-3.42023300	-1.03568200	-1.78756500
C	-3.74552800	-2.18728900	-0.83465200

C	-2.50348800	-3.03698700	-0.52699500
C	2.01671500	-1.46650300	-1.82983000
H	7.43152400	-0.85496100	-1.26137200
H	7.71003600	0.25201800	0.09488100
H	8.39047400	0.61519500	-1.50331800
H	6.37730800	2.03581300	-1.08716000
H	6.05445300	0.92186200	-2.41656500
H	4.97177600	-0.64243700	-0.76311800
H	3.52370000	0.78886900	-2.08239800
H	1.97562300	1.28981600	0.29043100
H	-0.08273200	0.57332000	0.06449200
H	-0.77371300	-1.54756600	-2.01710100
H	-0.40606500	-2.90870700	-0.14792100
H	-2.36880800	-1.74868800	1.83294700
H	-0.51878800	-1.26506600	3.25814200
H	1.27613200	-2.28030400	0.95255800
H	1.80793500	0.10531300	2.80240900
H	3.46534200	-1.33931400	0.66290900
H	4.39268700	3.04943200	-1.61332600
H	3.90038300	2.92250700	0.08448700
H	2.67547500	2.98231400	-1.19676900
H	-1.99490600	0.55908000	-2.06084700
H	-3.17436800	-1.45712000	-2.77026400
H	-4.30612700	-0.40373700	-1.90945700
H	-4.51389300	-2.82951500	-1.29050000
H	-2.24262100	-3.56873100	-1.45240200
H	-2.75914100	-3.81169100	0.20946700
H	2.60585200	-2.19400100	-1.25226600
H	2.68351500	-1.08062200	-2.61078400
H	1.21797400	-2.02133200	-2.32789900
H	-3.13227300	0.00377900	0.43402800
H	-4.51142300	-2.27470700	0.99231900
N	-4.69488000	2.83256000	-0.36257100
C	-5.06204900	2.92375400	1.05589100
C	-5.76097100	1.58832800	1.41365500
C	-6.42119500	1.14836800	0.07976900
C	-5.83723700	2.14017600	-0.97650600
H	-4.17860000	3.13248500	1.66810900
H	-3.88126300	2.21742200	-0.42390500
H	-5.76257200	3.76117700	1.18679100
H	-5.01328400	0.84830700	1.72394900
H	-6.47955700	1.68917100	2.23497500
H	-6.15993000	0.11159700	-0.15142800

H	-7.51447200	1.21228400	0.12232500
H	-5.52713000	1.64965400	-1.90613400
H	-6.59682300	2.88678800	-1.24548000

15

$G_{diethylether}$ = -1331.249378 Hartree

C	7.20321300	-0.10588400	-0.23565500
O	3.87949700	1.50220200	2.73212700
C	6.15069400	0.59986300	-1.09542800
O	4.70536100	1.07924800	0.71784400
C	4.73023800	0.39857600	-0.56336200
O	-2.30242900	0.47858300	0.00611600
C	3.61639700	0.91693900	-1.52393900
O	-4.27497900	-1.42149100	0.36340100
C	2.24073300	0.60711400	-0.97307900
C	1.57145600	-0.55947400	-0.97892500
C	0.35173700	-0.69368400	-0.08426800
C	-0.87051000	-1.40790700	-0.73149000
C	-1.43865600	-2.57751800	0.13142300
C	-1.53444400	-2.17141500	1.58347800
C	-0.47968200	-1.58502000	2.15331300
C	0.76256400	-1.34341400	1.33477200
C	1.75725400	-0.42147000	1.96626100
C	3.05304100	-0.44923300	1.63058400
C	3.90094700	0.75587300	1.78024200
C	3.75030100	2.42029500	-1.81406900
C	-1.94913700	-0.35551300	-1.09751700
C	-3.19314300	-1.00840400	-1.71887900
C	-3.78616400	-2.09250700	-0.81774300
C	-2.73528600	-3.15217800	-0.46981300
C	2.09922500	-1.80807300	-1.64356600
H	7.02964700	-1.18921800	-0.20600000
H	7.17233200	0.27051400	0.79127600
H	8.21082000	0.06071700	-0.63129700
H	6.36551000	1.67312900	-1.13481900
H	6.18719000	0.22482300	-2.12697100
H	4.57368500	-0.67574900	-0.41696000
H	3.77339200	0.36369100	-2.46035600
H	1.79477600	1.42909000	-0.41298500
H	0.01396800	0.31096800	0.18358400
H	-0.56845700	-1.85762900	-1.68616000

H	-0.68889700	-3.38326400	0.07570600
H	-2.44724800	-2.36595600	2.14056100
H	-0.49014800	-1.27179900	3.19472600
H	1.24423100	-2.30956700	1.11546400
H	1.37030400	0.44012900	2.50984700
H	3.42905600	-1.28012400	1.04304400
H	4.68444900	2.65462200	-2.33310300
H	3.72820100	2.99048500	-0.87921600
H	2.92116500	2.76117600	-2.44300100
H	-1.50181600	0.32713000	-1.82974100
H	-2.92675100	-1.46781700	-2.67925200
H	-3.94677600	-0.23913700	-1.91505100
H	-4.63288400	-2.57205500	-1.33131200
H	-2.49543900	-3.69470600	-1.39428000
H	-3.16681100	-3.89424100	0.21753100
H	2.35667400	-2.57246900	-0.89597800
H	2.99534400	-1.61845800	-2.23945700
H	1.35669700	-2.26758500	-2.30734300
H	-2.89746800	-0.04144400	0.57661000
H	-4.64192100	-2.08407300	0.96883800
N	-4.16034300	2.91951300	-0.42869200
C	-4.60551700	3.06126000	0.96310900
C	-5.49531500	1.82926600	1.26650300
C	-6.10670000	1.46511700	-0.11300500
C	-5.34078500	2.38129800	-1.11918000
H	-3.74436500	3.15370800	1.63292500
H	-3.43083700	2.20395400	-0.43767500
H	-5.19705800	3.98381200	1.05279200
H	-4.87440500	1.00139100	1.62859900
H	-6.25344700	2.02965100	2.03218100
H	-5.94523300	0.40531200	-0.33106500
H	-7.18641500	1.65009400	-0.14794200
H	-5.04043300	1.86218300	-2.03646400
H	-5.98260100	3.22034000	-1.42091300

16

$G_{\mathrm{diethylether}}$ = -1406.520176 Hartree

C	-7.09343800	-0.99847500	-0.79205600
O	-4.19486400	-0.53464800	2.92213100
C	-5.83393900	-1.80786800	-1.11411600
O	-4.69828900	-1.18127300	0.86575800

C	-4.56298500	-1.14790400	-0.57100300
O	2.50688900	0.84999200	0.40689200
C	-3.25018200	-1.84219000	-1.03283800
O	3.55005100	3.41135500	0.50883600
C	-2.00125400	-1.08464000	-0.64361400
C	-1.19051500	-0.33367500	-1.42430500
C	0.07469700	0.15586700	-0.86048000
C	1.09209000	0.70195300	-1.55187200
C	0.68561700	3.66488400	-0.25872500
C	0.50133600	3.76224300	1.07465400
C	-0.51934900	3.02788800	1.80318300
C	-1.64705500	2.52007200	1.24803800
C	-2.50812300	1.55924000	1.87641900
C	-3.44555500	0.88597800	1.17099500
C	-4.12863800	-0.28659400	1.73536000
C	-3.14900600	-3.28531200	-0.50022900
C	2.49021100	0.91428700	-1.02482200
C	3.26650100	2.14459400	-1.57025500
C	3.16479300	3.50832100	-0.85125700
C	1.82732400	4.29362600	-0.99269300
C	-1.47545700	-0.01993200	-2.87486600
H	-7.04482900	0.00386900	-1.23612200
H	-7.20718300	-0.88403300	0.29006100
H	-7.98943100	-1.49408600	-1.18089000
H	-5.92376500	-2.81305800	-0.68835400
H	-5.72438600	-1.92468500	-2.20055600
H	-4.54898900	-0.11222300	-0.92897400
H	-3.31956900	-1.88467900	-2.12661900
H	-1.69622300	-1.22882800	0.39272000
H	0.20568900	0.01138000	0.21033000
H	0.99509500	0.87559600	-2.62274800
H	0.04193500	3.00352000	-0.83064200
H	1.20606200	4.35323500	1.65713500
H	-0.32321200	2.82959000	2.85742000
H	-1.87409200	2.76252400	0.21070100
H	-2.32990100	1.28128700	2.91415700
H	-3.59035000	1.14123800	0.12919700
H	-3.97104000	-3.90944500	-0.86386900
H	-3.17876800	-3.29079800	0.59408200
H	-2.20905100	-3.74409800	-0.82478700
H	3.05604000	0.04317800	-1.40167600
H	3.02081100	2.27169000	-2.63371600
H	4.32928300	1.87851300	-1.52468000

H	3.92378700	4.14522700	-1.32539600
H	1.59087800	4.40496500	-2.05906900
H	2.02820000	5.29411800	-0.59189800
H	-2.46159300	-0.36127400	-3.19562300
H	-0.72898300	-0.48096500	-3.53480900
H	-1.42569700	1.06239300	-3.04867900
H	3.36160500	0.40943600	0.64497100
H	3.04301000	2.65906100	0.86833400
C	6.75082600	-4.62854500	0.23717500
C	5.90950100	-3.36886100	0.01912800
C	4.67766000	-3.32492600	0.93450600
C	3.91248400	-2.01819200	0.79673200
N	2.56235800	-2.09870000	0.74573900
O	4.51141100	-0.93478600	0.74434500
H	7.62331600	-4.63883600	-0.42510100
H	6.17003600	-5.53788900	0.03827600
H	7.11466200	-4.68652400	1.27044900
H	5.58173600	-3.31378800	-1.02751900
H	6.50541700	-2.46938500	0.20470800
H	5.00011600	-3.39590100	1.98271000
H	4.02379100	-4.18627300	0.74590800
H	2.07604200	-2.98097000	0.78093200
H	2.02619800	-1.23833600	0.67742900

17

$G_{\mathrm{diethylether}}$ = -1406.523278 Hartree

C	6.37168000	2.27086600	-1.33568900
O	5.07445100	-1.94893000	0.94162100
C	5.18610600	2.45197100	-0.38280100
O	5.06521900	0.15331700	0.22435900
C	4.27537200	1.22892400	-0.34247000
O	-2.47915800	-1.55612900	1.94096100
C	2.97358200	1.38782700	0.46145300
O	-4.15956200	-3.27894800	0.48038300
C	2.17590400	0.04378000	0.46398000
C	0.82541500	0.09107200	-0.22594100
C	-0.22171100	-0.52306700	0.36265600
C	-1.56528500	-0.82612400	-0.24059500
C	-1.59204500	-2.22361600	-0.97897900
C	-1.07533600	-3.40901800	-0.18040600
C	0.22386000	-3.70961600	0.01150800

C	1.29527600	-2.84724100	-0.47768900
C	2.38186500	-2.45934800	0.20746600
C	3.04851200	-1.16185200	-0.13354800
C	4.47112500	-1.05843800	0.39219300
C	3.22075500	1.86776900	1.89927700
C	-2.68781400	-0.70491100	0.81732000
C	-4.06272200	-0.92130100	0.16871300
C	-4.15842000	-2.26690800	-0.55206900
C	-3.00983200	-2.45673000	-1.55352100
C	0.77117200	0.60730800	-1.64910400
H	6.03034300	2.10924000	-2.36579000
H	6.97132900	1.40518600	-1.03871000
H	7.01790400	3.15504000	-1.33117400
H	5.55245600	2.65376900	0.62961400
H	4.58007700	3.31656500	-0.68524700
H	4.01888100	0.95890000	-1.37783000
H	2.38255400	2.15487900	-0.05396300
H	2.00286500	-0.23721100	1.50841800
H	-0.08376900	-0.93824300	1.35923700
H	-1.79483900	-0.08360700	-1.01240000
H	-0.92961900	-2.09784000	-1.84450300
H	-1.82078600	-4.03689400	0.29814000
H	0.48794000	-4.54239900	0.66206300
H	1.08502500	-2.32400100	-1.40890000
H	2.64474700	-2.90873100	1.16250400
H	3.08857800	-1.02444700	-1.22281400
H	3.66853500	2.86616000	1.92651900
H	3.88822600	1.18101200	2.43126700
H	2.27342700	1.91254000	2.44830400
H	-2.65217000	0.31549800	1.21736600
H	-4.24096300	-0.11880400	-0.55726800
H	-4.84345700	-0.86618600	0.93557000
H	-5.11407000	-2.32080600	-1.09624400
H	-3.17752100	-1.74367400	-2.37180600
H	-3.07205900	-3.45938000	-2.00079500
H	1.53604700	1.36703000	-1.84145400
H	-0.20064200	1.05287800	-1.88179700
H	0.94835000	-0.20441800	-2.36992400
H	-2.86059800	-2.42186600	1.71221300
H	-4.15480300	-4.14890000	0.05147100
C	-5.96018000	4.06662300	0.28584000
C	-4.80861800	3.09384400	0.02296300
C	-3.43108500	3.76831600	0.12561700

C	-2.32081400	2.78819000	-0.23553800
N	-1.31032000	2.65521200	0.66974200
O	-2.34902800	2.16118700	-1.29379100
H	-6.92969800	3.56308900	0.20250600
H	-5.94926900	4.89416400	-0.43417900
H	-5.89486600	4.50054700	1.29131600
H	-4.89572600	2.65503600	-0.97695100
H	-4.85518700	2.25860000	0.73534400
H	-3.28593000	4.19685500	1.12573400
H	-3.37942000	4.59846900	-0.59076200
H	-1.39219100	3.05409900	1.59320900
H	-0.65341600	1.88748700	0.55142800

18

$G_{diethylether}$ = -1406.516819 Hartree

C	-6.37653300	1.36932900	1.46602500
O	-4.77207500	-1.73288200	-1.73676700
C	-5.22371300	2.05497800	0.72710200
O	-4.68233200	0.16686800	-0.59683500
C	-4.10007100	1.08100700	0.36657300
O	2.10230100	-1.54569900	-1.89087400
C	-2.81598200	1.77334700	-0.18531500
O	4.11006400	-3.08769100	-0.72878300
C	-1.73282900	0.75084300	-0.45848000
C	-0.88378200	0.16883600	0.41197900
C	-0.09425800	-1.04510500	-0.04255400
C	1.38210300	-1.07601700	0.44507000
C	1.76883200	-2.40715900	1.15822700
C	1.24394500	-3.59652500	0.39022500
C	-0.03310100	-3.58808100	0.00167500
C	-0.89388900	-2.39561300	0.33415800
C	-2.20482000	-2.34693400	-0.38399800
C	-3.25082200	-1.66771900	0.10469300
C	-4.29560500	-1.13524200	-0.79901000
C	-3.09771200	2.60350100	-1.44769300
C	2.32302300	-0.73612100	-0.74026800
C	3.79480100	-0.76033200	-0.30218500
C	4.18912900	-2.09933300	0.32105400
C	3.27057600	-2.44875600	1.49681000
C	-0.78774000	0.56053000	1.86815500
H	-6.03384900	0.92379100	2.40859700

H	-6.80421600	0.57244400	0.85015200
H	-7.17223200	2.08355900	1.70329400
H	-5.59871700	2.51935200	-0.19136900
H	-4.79799300	2.85701600	1.34484500
H	-3.82105500	0.53647300	1.27503200
H	-2.49172500	2.45206000	0.61467000
H	-1.73220700	0.36896100	-1.47978600
H	-0.06601400	-1.05713900	-1.13600200
H	1.54429800	-0.27681200	1.17371000
H	1.23790700	-2.39650700	2.12427100
H	1.89965800	-4.43501700	0.16906700
H	-0.47202400	-4.41226400	-0.55588100
H	-1.07130100	-2.36479900	1.42113700
H	-2.21813200	-2.65812800	-1.42837700
H	-3.21413600	-1.30900200	1.12795900
H	-3.79157900	3.42581800	-1.25012700
H	-3.53177700	1.97151600	-2.22982200
H	-2.16825600	3.03673900	-1.83419500
H	2.07403400	0.28143000	-1.06173200
H	3.95919400	0.03276100	0.43634600
H	4.43265800	-0.55882500	-1.16998200
H	5.22882100	-2.04443700	0.67993200
H	3.47480400	-1.72749200	2.29912800
H	3.53627100	-3.43880900	1.89684800
H	-1.62598000	1.18179500	2.19477300
H	0.13873200	1.11835800	2.05410300
H	-0.76150200	-0.32852500	2.51083900
H	2.54024200	-2.39826400	-1.71933500
H	4.27904300	-3.95848000	-0.33701300
C	5.80034400	4.01821800	-0.36512200
C	4.64732500	3.05058700	-0.08930300
C	3.27468100	3.73238900	-0.17199700
C	2.14894400	2.77729900	0.21336200
N	1.04350500	2.80125800	-0.58032300
O	2.23887700	2.04826700	1.20112600
H	6.76810600	3.50892000	-0.29806100
H	5.80536200	4.84331400	0.35794500
H	5.72327800	4.45660300	-1.36789000
H	4.74634400	2.60720300	0.90727000
H	4.67916100	2.21739900	-0.80384500
H	3.11514400	4.15664600	-1.17189800
H	3.24176800	4.57175600	0.53631700
H	1.04170000	3.30061900	-1.45701900

H	0.30987000	2.11269100	-0.42968000

19

$G_{diethylether}$ = -1277.118516 Hartree

C	5.70307900	-1.90791900	1.62667600
O	3.22213700	2.01899300	1.21291900
C	5.04868500	-1.99306000	0.24478000
O	3.82974600	0.02532800	0.47806200
C	3.64318500	-1.38576600	0.22751200
O	-3.21120500	0.51767900	-1.47311700
C	2.87138200	-1.62026400	-1.10032200
O	-5.45407500	0.62264300	0.14195700
C	1.45025000	-1.10862000	-1.06280400
C	0.29929000	-1.80834900	-0.93495900
C	-0.96684200	-1.09132800	-1.12313500
C	-2.19270300	-1.63865000	-1.20945600
C	-3.33616200	-0.67878400	1.73513600
C	-3.09406600	0.51429000	2.31655500
C	-1.77192700	1.12058000	2.37848700
C	-0.61276500	0.42329900	2.31324600
C	0.67653500	1.00372900	2.05985100
C	1.72915700	0.23657000	1.69715000
C	2.95474500	0.83398500	1.14767600
C	3.59363900	-0.98487000	-2.30541600
C	-3.41866800	-0.88871200	-1.67071700
C	-4.78749400	-1.37707700	-1.12063800
C	-5.35814100	-0.79492000	0.19395600
C	-4.70113600	-1.25395700	1.52651200
C	0.23643700	-3.29417200	-0.67152200
H	5.11603900	-2.45100900	2.37818300
H	5.78277700	-0.86500400	1.94813700
H	6.70965400	-2.33934900	1.61211500
H	5.67080100	-1.47106800	-0.48992800
H	4.97513300	-3.03999400	-0.07849100
H	3.07495300	-1.84291600	1.04516900
H	2.86288300	-2.70895600	-1.23680200
H	1.35906800	-0.03684900	-1.23265400
H	-0.88466500	-0.01829000	-1.26978000
H	-2.33680900	-2.71158900	-1.09222300
H	-2.51072700	-1.21190900	1.27272800
H	-3.93252100	1.09659700	2.69832700

H	-1.72776300	2.20926200	2.41387900
H	-0.65599400	-0.66494800	2.32985200
H	0.77812300	2.08704000	2.04160200
H	1.60223400	-0.83730500	1.68338600
H	4.58434700	-1.42281200	-2.46111100
H	3.71569800	0.09204600	-2.15178300
H	3.00996000	-1.13689200	-3.21905000
H	-3.46936700	-1.07962300	-2.75896100
H	-4.76831600	-2.47383500	-1.05817000
H	-5.53542600	-1.13006200	-1.88518000
H	-6.39680800	-1.14559500	0.24049200
H	-4.66782400	-2.35149000	1.54409200
H	-5.37485600	-0.92796100	2.32733000
H	1.22112000	-3.73715500	-0.50818600
H	-0.23744500	-3.82463400	-1.50767100
H	-0.37214400	-3.49983800	0.21863000
H	-3.91776400	0.98003600	-1.95174800
H	-4.53446000	0.94207600	0.11088700
C	1.21763200	4.50281300	-0.35099100
C	0.63085800	3.65063600	-1.48590200
C	1.72229300	2.76864600	-2.11114500
C	-0.54828900	2.80222800	-0.99107500
H	2.01408700	5.15976400	-0.72127900
H	0.44883300	5.13453100	0.11171600
H	1.65840700	3.86412900	0.42400800
H	0.25176300	4.32937400	-2.26539200
H	2.20119100	2.15231600	-1.34072700
H	1.31286600	2.10403400	-2.88258600
H	2.51000300	3.37675400	-2.57196300
H	-0.21663300	2.06598800	-0.24998800
H	-1.31720700	3.42312700	-0.51394200
H	-1.02989700	2.25290200	-1.80822900

20

$G_{diethylether}$ = -1277.127332 Hartree

C	6.26129300	-2.19150000	0.29180100
O	3.36016500	1.63284400	1.61116500
C	5.19880000	-1.84929200	-0.75711600
O	4.19176900	-0.12128300	0.53292300
C	3.89071700	-1.37435300	-0.13250300
O	-3.31990100	0.66085800	-1.45920300

C	2.71725600	-1.17073800	-1.10650200
O	-5.59111800	0.15571100	0.14510900
C	1.47490200	-0.59349100	-0.35162800
C	0.22283900	-1.45038600	-0.39806500
C	-0.95514000	-0.83806600	-0.61271500
C	-2.34797900	-1.38928400	-0.47557300
C	-2.91989700	-1.24254700	0.99292200
C	-2.82532400	0.14033600	1.61760900
C	-1.71792400	0.68219200	2.15938400
C	-0.41947900	0.01872200	2.10543200
C	0.73934400	0.60389800	1.76549000
C	1.84406900	-0.20702900	1.16350300
C	3.17339300	0.53078300	1.15010600
C	3.07983500	-0.26685600	-2.29436200
C	-3.29016800	-0.76021200	-1.53304400
C	-4.69852700	-1.36892100	-1.44572500
C	-5.30422100	-1.24621400	-0.04621400
C	-4.36103400	-1.80542700	1.03043400
C	0.35597500	-2.89704600	0.01082400
H	5.92310600	-3.00335700	0.94785800
H	6.47567800	-1.31932300	0.91679800
H	7.19446700	-2.51123600	-0.18398200
H	5.57559900	-1.06701100	-1.42473100
H	4.97875900	-2.72647900	-1.38029800
H	3.59149100	-2.10646700	0.63251900
H	2.47392400	-2.16514600	-1.49902600
H	1.22582100	0.36341100	-0.81746700
H	-0.93946500	0.22580500	-0.83288300
H	-2.34606000	-2.47134200	-0.67346300
H	-2.30566800	-1.91873800	1.60138900
H	-3.72668500	0.74454400	1.59617300
H	-1.75442600	1.70873300	2.52144200
H	-0.44161800	-1.06859200	2.14610500
H	0.82377900	1.68399100	1.68051300
H	1.98331200	-1.14479700	1.71812000
H	3.86258300	-0.70597700	-2.92107200
H	3.42773800	0.71227900	-1.94740100
H	2.19797000	-0.10713300	-2.92414400
H	-2.87153300	-0.98823700	-2.52147400
H	-4.65223200	-2.43293300	-1.71074500
H	-5.35469100	-0.87241700	-2.16850400
H	-6.25125800	-1.80689000	-0.01147000
H	-4.32094500	-2.89420100	0.88782800

H	-4.79940400	-1.64341200	2.02547700
H	0.78454400	-2.98396700	1.02019500
H	1.02420400	-3.45621500	-0.65621400
H	-0.60489800	-3.41747100	0.02129200
H	-3.97305900	0.90052400	-0.77886000
H	-5.93531900	0.27610500	1.04393100
C	0.49022300	4.89939100	0.30732400
C	0.17230900	4.04090800	-0.92527400
C	1.40461400	3.22313000	-1.34037400
C	-1.04157800	3.13778200	-0.66925600
H	1.33977500	5.56779400	0.12243700
H	-0.36945300	5.51572400	0.59766100
H	0.75296300	4.26356900	1.16337200
H	-0.07975800	4.71771800	-1.75584400
H	1.76800400	2.61492500	-0.50296200
H	1.17756600	2.55283100	-2.17880700
H	2.23216000	3.87551500	-1.64448200
H	-0.83769200	2.42369300	0.13637800
H	-1.91810100	3.72629900	-0.37006600
H	-1.32427300	2.55816600	-1.55442200

21

$G_{diethylether}$ = -1277.120520 Hartree

C	-6.05610600	-1.76729900	-0.82440300
O	-3.14087400	1.97900600	-1.33285900
C	-5.23257800	-1.64242800	0.46066300
O	-3.91848000	0.16712500	-0.31873500
C	-3.79738700	-1.18537600	0.19322400
O	2.87368000	0.75512800	1.11894600
C	-2.86652200	-1.25272200	1.44256100
O	5.34876800	0.03899200	0.07705600
C	-1.45794400	-0.83491300	1.08182700
C	-0.49208400	-1.54283700	0.46927600
C	0.71553500	-0.79258000	-0.06283800
C	2.09262100	-1.44301800	0.25133600
C	2.99650300	-1.62637500	-1.00569700
C	2.97116900	-0.38628000	-1.86764500
C	1.79343200	0.16512300	-2.16628600
C	0.52629700	-0.44925400	-1.62860000
C	-0.67305100	0.43900100	-1.71413800
C	-1.92195500	-0.04365900	-1.68728600

C	-3.02330600	0.78676400	-1.15138900
C	-3.39094600	-0.39742500	2.60721600
C	2.78986400	-0.64135200	1.38407900
C	4.17207300	-1.22326400	1.72050800
C	5.07551100	-1.31578200	0.48930600
C	4.40314300	-2.12533800	-0.62533800
C	-0.65105300	-2.99270100	0.08142700
H	-5.61490500	-2.50370100	-1.50821500
H	-6.09985400	-0.80567800	-1.34464300
H	-7.08113600	-2.08550700	-0.60599800
H	-5.71158000	-0.92738900	1.13806300
H	-5.19348300	-2.60717600	0.98394500
H	-3.37263100	-1.83891100	-0.57658900
H	-2.87865200	-2.30903400	1.74575100
H	-1.25798200	0.22062000	1.25889100
H	0.73250300	0.19265200	0.40749000
H	1.93633000	-2.45005500	0.66081900
H	2.53120900	-2.43235600	-1.59624000
H	3.90268700	0.03265500	-2.23967100
H	1.71935200	1.05822200	-2.78189200
H	0.33038400	-1.40036600	-2.14845500
H	-0.51747000	1.49774500	-1.52088400
H	-2.08498300	-1.10674200	-1.82518400
H	-4.35839500	-0.75315400	2.97414000
H	-3.50829200	0.64398400	2.28866200
H	-2.68398800	-0.42116000	3.44326200
H	2.14737800	-0.72049300	2.27025800
H	4.05818800	-2.23140900	2.13951300
H	4.64932900	-0.59570700	2.48027300
H	6.02321000	-1.80240900	0.76760200
H	4.33574300	-3.16660600	-0.28200700
H	5.04715900	-2.13880500	-1.51704100
H	-0.76724400	-3.09764600	-1.00737600
H	-1.52296200	-3.45759200	0.54897300
H	0.22692900	-3.59067900	0.35393200
H	3.61992900	0.88204900	0.50563400
H	5.85426200	0.01070100	-0.74989800
C	-0.25684500	5.37032300	0.33741800
C	0.48944800	4.08104300	0.70552400
C	-0.48239300	3.01753600	1.23684200
C	1.29313400	3.54874500	-0.48945700
H	-0.79594500	5.78062900	1.20006800
H	0.43276400	6.14146500	-0.02798100

H	-0.99330500	5.17528700	-0.45304000
H	1.20306200	4.31341200	1.51017400
H	-1.22333300	2.74370200	0.47398900
H	0.06368600	2.11559800	1.53673600
H	-1.03348700	3.38456400	2.11168100
H	0.63276300	3.37468600	-1.34974800
H	2.05571600	4.27100000	-0.80746100
H	1.79293600	2.60600400	-0.24156400

22

$G_{diethylether}$ = -1367.229436 Hartree

C	-4.16592300	0.56252400	2.70342700
O	-2.44165500	-2.33144600	-0.62261800
C	-3.72676200	1.43121400	1.52080900
O	-2.79507400	-0.34358400	0.26042000
C	-2.43511900	0.92260000	0.87493000
O	4.17959400	0.21373600	-1.39886900
C	-1.82768600	1.90079600	-0.17149800
O	6.81453500	-0.14057300	-1.08020700
C	-0.39427800	1.55792800	-0.48985500
C	0.74291300	2.14584400	-0.05495200
C	2.02267400	1.59093600	-0.51219000
C	3.24925600	2.07937300	-0.26272500
C	4.58427900	-1.53919800	0.76837600
C	4.10775300	-2.72351800	0.34357600
C	2.69155300	-2.96565100	0.09320000
C	1.68138800	-2.20200500	0.57865300
C	0.30430400	-2.30225200	0.18380200
C	-0.61986100	-1.39395700	0.57846900
C	-1.98311300	-1.40691400	0.03702100
C	-2.65601300	1.95696500	-1.46898300
C	4.51649500	1.43621600	-0.76770700
C	5.55941500	1.29410300	0.39910100
C	6.52625300	0.09324200	0.31032600
C	6.01162700	-1.18090200	1.03197300
C	0.78514200	3.33734700	0.87190600
H	-3.40494500	0.55362100	3.49443700
H	-4.33504700	-0.47067600	2.38601400
H	-5.09836800	0.93623100	3.13889200
H	-4.51671200	1.46556200	0.76330300
H	-3.55272300	2.46020400	1.86092200

H	-1.69803600	0.75141100	1.66795300
H	-1.87065900	2.88524100	0.30888000
H	-0.27841200	0.71028300	-1.16526000
H	1.96066400	0.69016000	-1.11813000
H	3.39093500	2.98520200	0.32499900
H	3.87597100	-0.73287300	0.92951800
H	4.79994800	-3.53337400	0.11370000
H	2.44394700	-3.79887900	-0.56457300
H	1.91627400	-1.39613800	1.27191500
H	0.00369200	-3.09054600	-0.50455900
H	-0.31814200	-0.58513300	1.23185800
H	-3.72526300	2.08876000	-1.28247700
H	-2.53582800	1.02403300	-2.02889100
H	-2.30133800	2.77632600	-2.10262600
H	4.97636600	2.11089700	-1.51117600
H	5.04164800	1.23386600	1.36366600
H	6.14491900	2.21991300	0.42326700
H	7.46440800	0.37426800	0.81219500
H	6.13110200	-0.98581300	2.11001300
H	6.68164100	-2.02120100	0.79905300
H	-0.20692700	3.66993800	1.18382200
H	1.28690000	4.18599600	0.38943400
H	1.36109000	3.10256900	1.77630900
H	5.02564000	-0.18254600	-1.67529300
H	7.42975300	-0.88754800	-1.14201700
C	-6.99436700	-0.85192400	0.80813500
C	-5.93131000	-1.42407600	-0.12625200
C	-5.50734500	-0.43458600	-1.20857900
N	-4.67701000	-0.96177200	-2.16484400
O	-5.90642000	0.72715100	-1.24088400
H	-6.64058200	0.06511100	1.28645100
H	-7.25380100	-1.57501200	1.58923400
H	-7.90287800	-0.59676600	0.25400700
H	-6.28881000	-2.33994300	-0.61414900
H	-5.02656700	-1.71196500	0.42362700
H	-4.03470500	-1.68955200	-1.85646300
H	-4.26157100	-0.26611400	-2.77245900

23

$G_{diethylether}$ = -1367.236697 Hartree

C	-5.53559100	-1.26446900	-1.32152600

O	-3.05108300	0.39815800	2.55049500
C	-4.37229000	-2.21309700	-1.00759300
O	-3.61433300	-0.87270500	0.81896800
C	-3.16809000	-1.47711500	-0.43277400
O	4.27621800	-1.62918700	1.12222800
C	-1.91413400	-2.32151300	-0.15913600
O	6.13885800	0.42269600	0.59572200
C	-0.77163400	-1.40636900	0.38736000
C	0.38739500	-1.15662300	-0.56450700
C	1.63569300	-1.15193400	-0.06326200
C	2.91246500	-0.68966000	-0.71120600
C	3.20710400	0.84218500	-0.44181200
C	3.14546300	1.28728100	1.01092500
C	2.01864500	1.53867600	1.70511500
C	0.69710900	1.31202100	1.13264100
C	-0.34176500	0.70123400	1.72612100
C	-1.35227300	0.00719600	0.86897200
C	-2.71680900	-0.13297200	1.51322900
C	-2.18142100	-3.48833500	0.80332700
C	4.10337200	-1.58720300	-0.28987700
C	5.39155500	-1.16218900	-1.01272000
C	5.73121600	0.31238900	-0.78552300
C	4.54318500	1.22681200	-1.12126800
C	0.03591100	-0.72101900	-1.96817900
H	-5.20011900	-0.42564500	-1.94289900
H	-5.95556100	-0.85771000	-0.39501800
H	-6.34041900	-1.78646800	-1.84987800
H	-4.70174900	-2.99214700	-0.31121300
H	-4.03982600	-2.71877500	-1.92384900
H	-2.91743300	-0.66134400	-1.12019700
H	-1.60539000	-2.74160300	-1.12371400
H	-0.36663000	-1.87712500	1.28901200
H	1.76793900	-1.42960500	0.98015700
H	2.83302500	-0.77806500	-1.80421300
H	2.41834300	1.37946000	-0.98377000
H	4.09381900	1.37662000	1.53139800
H	2.08996800	1.78671900	2.76336200
H	0.62097400	1.47200800	0.05940000
H	-0.33698300	0.46509500	2.78816300
H	-1.52139200	0.58613000	-0.04519200
H	-2.91762900	-4.19036500	0.39908400
H	-2.55571100	-3.12506800	1.76675400
H	-1.25604500	-4.04491900	0.98665800

H	3.85966900	-2.61578900	-0.58501300
H	5.27617900	-1.32863800	-2.09130100
H	6.22544900	-1.78214900	-0.66629000
H	6.58282300	0.59056600	-1.42559700
H	4.40830800	1.19056100	-2.21106000
H	4.79948800	2.26838600	-0.87998200
H	-0.47290000	-1.51828000	-2.52562000
H	0.91660400	-0.43212400	-2.54700600
H	-0.65324500	0.13546500	-1.96764200
H	4.83837300	-0.87200900	1.36248800
H	6.31176900	1.35751700	0.78816900
C	-1.27772800	3.69491300	-1.14392700
C	-2.34483500	3.35983800	-0.10447800
C	-3.32806900	2.30033100	-0.59368900
N	-4.41815300	2.10198300	0.20973000
O	-3.15362900	1.66087100	-1.63087900
H	-0.72761400	2.79614900	-1.43762000
H	-0.56500400	4.42202400	-0.74171400
H	-1.72830800	4.10931600	-2.05090700
H	-2.90407300	4.25597500	0.19341800
H	-1.87485000	2.97186600	0.81104100
H	-4.34774200	2.36091000	1.18627600
H	-4.92212400	1.23825800	0.04666200

24

$G_{\text{diethylether}}$ = -1367.223241 Hartree

C	-5.79599400	0.22463600	-0.94007600
O	-3.25190400	-0.32260900	3.04551300
C	-4.99765300	-1.07405400	-1.08693100
O	-3.85544300	-0.83381600	0.97214700
C	-3.60935900	-0.97607700	-0.45144200
O	3.25737000	-2.26467100	1.10207100
C	-2.67676900	-2.18493000	-0.76783000
O	5.59149900	-0.78616600	0.71475300
C	-1.28336400	-1.94787000	-0.22274100
C	-0.30030500	-1.18812200	-0.73783100
C	0.89464100	-0.85354700	0.13860100
C	2.25595600	-0.77195200	-0.61249200
C	3.03290400	0.55938600	-0.35647900
C	3.02150500	0.90243400	1.11411200
C	1.85110100	0.86897300	1.75573500

C	0.61386400	0.50080800	0.97929400
C	-0.61047700	0.30143600	1.80898600
C	-1.83459300	0.46512000	1.28823600
C	-3.01261100	-0.21908900	1.86359600
C	-3.24614700	-3.50998400	-0.23716400
C	3.10247700	-2.03227400	-0.29255100
C	4.46429400	-1.99221300	-1.00570800
C	5.24982700	-0.71898200	-0.68581800
C	4.42614900	0.53227800	-1.01029700
C	-0.40604700	-0.50137300	-2.08139100
H	-5.27711700	1.06294400	-1.42459100
H	-5.92750700	0.47398900	0.11721100
H	-6.78694500	0.13620900	-1.39806200
H	-5.54647000	-1.89986800	-0.62168600
H	-4.87307000	-1.32606100	-2.14848400
H	-3.12788000	-0.07050100	-0.83655100
H	-2.64007900	-2.23515600	-1.86452200
H	-1.11520900	-2.37743600	0.76485100
H	0.99895600	-1.62710400	0.90428600
H	2.07644900	-0.80220700	-1.69418400
H	2.46065900	1.34731600	-0.87003900
H	3.94571700	1.16134700	1.62419000
H	1.76554600	1.09025100	2.81724400
H	0.42490600	1.27258800	0.22496800
H	-0.50318300	-0.20602200	2.76760200
H	-1.92929200	0.91628800	0.30714400
H	-4.20368200	-3.76038400	-0.70432500
H	-3.40209100	-3.45031700	0.84522000
H	-2.54845600	-4.32973300	-0.43824200
H	2.53873700	-2.89901400	-0.66162300
H	4.31551900	-2.03832300	-2.09240800
H	5.04959700	-2.86891700	-0.70904000
H	6.17827700	-0.70491700	-1.27801600
H	4.31085000	0.57920700	-2.10163000
H	4.98901800	1.43340400	-0.72469500
H	-1.42416500	-0.52139100	-2.48035500
H	0.23680900	-0.98698500	-2.82837200
H	-0.08446300	0.54506000	-2.02719900
H	3.94698900	-1.65115000	1.41234200
H	6.01960000	0.04824500	0.96123300
C	0.70219000	4.36150700	0.70279200
C	-0.70936700	3.92694800	0.31118900
C	-0.74564900	3.19575800	-1.02656700

N	-1.99827500	2.86536500	-1.47887600
O	0.26311900	2.85889100	-1.63795900
H	1.35795900	3.49513300	0.82481800
H	0.67917000	4.91175700	1.64899400
H	1.14021600	5.00605200	-0.06524400
H	-1.38838000	4.78980200	0.26403200
H	-1.12762200	3.24901800	1.06810600
H	-2.82800100	3.27856200	-1.07766300
H	-2.07000500	2.47482800	-2.40870700

25

$G_{diethylether}$ = -1429.523873 Hartree

C	-7.70895500	-1.31855200	-0.88516400
O	-5.40964600	0.49207400	2.63972100
C	-6.47870500	-2.22343000	-0.77293500
O	-5.49739600	-0.97029400	0.98182900
C	-5.22297100	-1.45280500	-0.35351100
O	1.62290200	0.83036900	0.68661400
C	-3.92294400	-2.30465400	-0.39167700
O	3.07182800	3.05004800	0.09930600
C	-2.67620400	-1.48397300	-0.16336200
C	-1.72680800	-1.11687400	-1.05451700
C	-0.53294500	-0.41947600	-0.55780200
C	0.58609100	-0.15379800	-1.25565900
C	0.21097700	3.23966000	-0.77666100
C	-0.35402200	4.02006600	0.16288100
C	-1.50884800	3.58845000	0.94522400
C	-2.43162900	2.72222300	0.46659700
C	-3.45496400	2.06158700	1.23304000
C	-4.18766500	1.06659000	0.68680600
C	-5.07027700	0.21811600	1.50682800
C	-3.96377600	-3.46045500	0.62779100
C	1.86925900	0.35062100	-0.62888400
C	2.72298400	1.31458200	-1.51551000
C	2.68787600	2.83451100	-1.27697600
C	1.39287300	3.60084700	-1.61741800
C	-1.79037200	-1.42400100	-2.53279100
H	-7.55438500	-0.52986400	-1.63253800
H	-7.91931300	-0.83844400	0.07524600
H	-8.59313800	-1.89269300	-1.18223700
H	-6.67068800	-3.01687700	-0.04274900

H	-6.27624000	-2.71143800	-1.73562300
H	-5.10746700	-0.60808500	-1.04232500
H	-3.89486700	-2.73635100	-1.39942100
H	-2.51373300	-1.19955600	0.87621100
H	-0.54061200	-0.13852100	0.49349300
H	0.67247200	-0.44244700	-2.30314900
H	-0.17529500	2.23526400	-0.92045100
H	0.06931400	5.00196100	0.38322400
H	-1.59734600	3.95701700	1.96728000
H	-2.35869900	2.42276400	-0.57810500
H	-3.58052800	2.29902700	2.28789300
H	-4.02074300	0.81706200	-0.35247800
H	-4.79041900	-4.14900000	0.42640300
H	-4.08742200	-3.07015000	1.64289100
H	-3.03058700	-4.03138400	0.58874900
H	2.50360700	-0.55004200	-0.54040300
H	2.49167700	1.13323200	-2.57235200
H	3.77071100	1.03105200	-1.37219600
H	3.47406000	3.25890600	-1.92091300
H	1.17341800	3.41218900	-2.67840700
H	1.60863300	4.67670800	-1.53281400
H	-2.73456100	-1.88328200	-2.83219700
H	-0.97885600	-2.10187400	-2.82771100
H	-1.66414200	-0.50584900	-3.12042700
H	2.09563800	1.67759400	0.78639300
H	2.80194100	3.94452500	0.36062400
C	6.54457900	-4.11749000	-0.56317200
C	6.91218300	-3.42265200	0.76021400
C	6.41140300	-1.99530900	0.81404300
C	7.20940600	-0.93576200	0.36276500
C	5.11653600	-1.70690700	1.26631300
C	6.72825900	0.37483300	0.36210000
C	4.62826200	-0.39934700	1.26933000
C	5.43540100	0.64833800	0.81440500
H	6.91240000	-5.15003000	-0.58450200
H	6.97695600	-3.58158200	-1.41593700
H	5.45747200	-4.13801400	-0.70120600
H	8.00221400	-3.43890200	0.88703300
H	6.49135900	-3.99448700	1.59717400
H	8.21853100	-1.14172700	0.01074000
H	4.48410600	-2.51818000	1.62203200
H	7.36569300	1.18325300	0.01173800
H	3.61714200	-0.19287700	1.60885300

H	5.05017000	1.66389400	0.80863900

26

$G_{diethylether}$ = -1429.531780 Hartree

C	-8.04153800	1.85191300	1.12740900
O	-5.54707800	-0.14803400	-2.68268300
C	-6.62867600	2.42274500	0.97151200
O	-6.04413400	1.00942900	-0.85238900
C	-5.62641500	1.38030600	0.48574400
O	1.73304700	-0.28577500	-0.60232800
C	-4.15446500	1.82628400	0.45748100
O	3.00885600	-2.74491000	-0.13545200
C	-3.25374700	0.67632900	-0.10027200
C	-2.24664000	0.09628400	0.87850600
C	-1.00387800	-0.16940700	0.43759100
C	0.08657200	-0.95844300	1.11006000
C	0.01911200	-2.50219400	0.76344000
C	-0.07425700	-2.85198000	-0.71362900
C	-1.18986000	-2.78456900	-1.46516100
C	-2.44981900	-2.26742900	-0.94198400
C	-3.25940200	-1.39130200	-1.55650600
C	-4.14082700	-0.49997500	-0.73889000
C	-5.28689100	0.10710600	-1.53105800
C	-3.94256400	3.11437400	-0.35202600
C	1.47929200	-0.35618900	0.80041800
C	2.58411500	-1.11661800	1.54995000
C	2.57440800	-2.61395500	1.23861600
C	1.18741900	-3.23248200	1.46927000
C	-2.77167400	-0.34839800	2.22239800
H	-8.06143000	1.04033000	1.86554400
H	-8.39836400	1.45032500	0.17421100
H	-8.74304300	2.62462400	1.45936100
H	-6.64276200	3.25729700	0.26228300
H	-6.26796600	2.81986900	1.92986900
H	-5.71431400	0.49422300	1.13257300
H	-3.87468500	2.03174700	1.49751100
H	-2.68949100	1.08095400	-0.94683800
H	-0.74584200	0.13424300	-0.57449800
H	-0.03031800	-0.90607900	2.20213600
H	-0.90233000	-2.85624200	1.24297900
H	0.84834400	-3.15302100	-1.20011200

H	-1.12375800	-2.99591000	-2.53171800
H	-2.63465900	-2.45955700	0.11361600
H	-3.11842200	-1.11940600	-2.60016900
H	-4.58528000	-1.06231400	0.09344600
H	-4.47190900	3.96563900	0.08756900
H	-4.29456200	2.99106200	-1.38216200
H	-2.87685300	3.36528900	-0.38425600
H	1.47553200	0.68535000	1.14396300
H	2.43814100	-0.99046500	2.63034100
H	3.56135300	-0.69541800	1.29586800
H	3.30453900	-3.12354700	1.88513900
H	1.00958500	-3.22125800	2.55340700
H	1.19919400	-4.29086500	1.17239300
H	-3.55445400	-1.11273600	2.10811500
H	-3.22751600	0.47794400	2.78188100
H	-1.99102500	-0.77845000	2.85440400
H	2.08354000	-1.15294000	-0.87292300
H	2.98448000	-3.68390600	-0.37771800
C	7.94801100	3.73433100	0.81752000
C	7.88035900	3.13303500	-0.59775300
C	7.12390300	1.82224000	-0.62698400
C	7.78869200	0.60524900	-0.42469500
C	5.73428900	1.79921800	-0.80534800
C	7.08556400	-0.60021500	-0.40121900
C	5.02340900	0.59787500	-0.78341400
C	5.70031700	-0.60865500	-0.58030200
H	8.49787100	4.68287100	0.81986200
H	8.44864600	3.04589300	1.50798400
H	6.94102200	3.92072400	1.20800900
H	8.89983200	2.98193100	-0.97537600
H	7.40034100	3.85269300	-1.27327800
H	8.86848600	0.60499600	-0.28718300
H	5.20365600	2.73584800	-0.96698100
H	7.62158900	-1.53403600	-0.24782600
H	3.94509200	0.59503500	-0.92024800
H	5.14335200	-1.54129500	-0.56240100

27

$G_{diethylether}$ = -1429.524954 Hartree

C	7.74038200	1.46475600	-0.65164800
O	4.79738200	0.80517500	3.00435300

C	6.42971600	2.17231400	-1.00746900
O	5.29318700	1.47534200	0.94938900
C	5.20042500	1.41725100	-0.49741400
O	-1.48228100	-0.52918500	0.66335200
C	3.84640900	1.98710300	-1.02061300
O	-2.95704400	-2.85109300	0.29945700
C	2.68468900	1.13914100	-0.54802900
C	2.26051100	-0.04833200	-1.01583300
C	1.28111100	-0.84652100	-0.17341100
C	0.11575600	-1.51192700	-0.96185600
C	-0.04367000	-3.03638400	-0.66865500
C	0.06441600	-3.30854500	0.81324600
C	1.07805300	-2.76556800	1.49040300
C	2.07186500	-1.89842800	0.76132300
C	2.97474300	-1.10152800	1.64877800
C	4.16496900	-0.65796200	1.22521500
C	4.78011300	0.54972700	1.82187600
C	3.63726300	3.45491100	-0.61582400
C	-1.19428600	-0.71534900	-0.72069100
C	-2.38320100	-1.34555300	-1.46283900
C	-2.57793100	-2.81697500	-1.09493900
C	-1.29752100	-3.62021900	-1.34740800
C	2.87993300	-0.72997900	-2.21212800
H	7.78226400	0.46141000	-1.09453000
H	7.83551600	1.36038700	0.43337600
H	8.60459400	2.02872500	-1.01863200
H	6.42769300	3.18148000	-0.58172400
H	6.33852200	2.28073800	-2.09651600
H	5.27896200	0.37779600	-0.83408200
H	3.92310000	1.93288800	-2.11549500
H	2.20603400	1.51214100	0.35753200
H	0.81551400	-0.16660400	0.54512500
H	0.31036300	-1.43204400	-2.03924100
H	0.82122600	-3.52631700	-1.14478200
H	-0.67130700	-3.94173600	1.30246900
H	1.20712400	-2.92672100	2.55817300
H	2.67790800	-2.52804300	0.09054500
H	2.54098500	-0.67130500	2.55120300
H	4.57390600	-1.04585100	0.29821200
H	4.39425900	4.11183600	-1.05446300
H	3.68689700	3.55855200	0.47339800
H	2.65438700	3.80445100	-0.94917400
H	-1.03169200	0.29707900	-1.11040800

H	-2.21212600	-1.28655500	-2.54557800
H	-3.29369200	-0.78271800	-1.23796400
H	-3.39792500	-3.23846600	-1.69555700
H	-1.13809000	-3.65132000	-2.43370300
H	-1.44050800	-4.66346700	-1.02936100
H	3.42053800	-1.63884800	-1.90999200
H	3.58926800	-0.08783300	-2.74006400
H	2.12506500	-1.05120800	-2.94002200
H	-1.85433900	-1.36817100	0.99016100
H	-3.03989700	-3.77791000	0.57265400
C	-7.26782400	3.99117600	-0.99294000
C	-7.27506200	3.44681400	0.44675200
C	-6.64071300	2.07580000	0.54312900
C	-7.41080000	0.91566700	0.38546400
C	-5.26040100	1.93613400	0.74015200
C	-6.81903900	-0.34795300	0.42353200
C	-4.66056400	0.67613700	0.77947200
C	-5.44185600	-0.47255700	0.62033700
H	-7.72906400	4.98452900	-1.04409600
H	-7.81908600	3.32197600	-1.66347900
H	-6.24260400	4.06918100	-1.37251100
H	-8.30926500	3.40410200	0.81194300
H	-6.74144700	4.14848400	1.10066200
H	-8.48495100	1.00626600	0.23416000
H	-4.64900100	2.82773400	0.86766200
H	-7.43590900	-1.23576100	0.30414700
H	-3.58820800	0.58091900	0.92902500
H	-4.96988700	-1.45063100	0.65063300

28

$G_{\text{diethylether}}$ = -1316.410387 Hartree

C	-5.29282600	1.41676100	-0.67785800
O	-3.30027800	1.59920100	2.80037100
C	-5.02214200	-0.09070100	-0.69554400
O	-3.76532000	0.00117200	1.32887900
C	-3.68257400	-0.45109900	-0.04434100
O	2.67563900	-1.28222400	1.37289900
C	-3.34215500	-1.97015700	-0.09764400
O	5.35838800	-1.14117900	1.22291200
C	-1.90737300	-2.16572400	0.30873900
C	-0.82946600	-2.45204300	-0.45597700

C	0.49447800	-2.30694400	0.15830600
C	1.69204400	-2.46288400	-0.43084400
C	3.54590400	1.32164200	0.39621600
C	3.26355600	1.99668300	1.52522100
C	1.89909900	2.23127300	1.99279100
C	0.85202800	1.45783400	1.61425800
C	-0.52680200	1.63434900	1.99135300
C	-1.49199300	0.85745300	1.44641200
C	-2.89383100	0.89374700	1.89996400
C	-4.28508000	-2.81191400	0.78086900
C	2.98732100	-2.10928100	0.26178500
C	3.98175700	-1.44905400	-0.74098200
C	5.13444200	-0.63304400	-0.10425200
C	4.89353300	0.90311100	-0.09819600
C	-0.89344400	-2.83055600	-1.91571300
H	-4.50854600	1.96716700	-1.21166400
H	-5.32460000	1.78793300	0.35098700
H	-6.25071400	1.64803900	-1.15631300
H	-5.82830800	-0.61546000	-0.17086900
H	-5.01047800	-0.46516000	-1.72821500
H	-2.89158600	0.10086200	-0.56595900
H	-3.47929100	-2.26372000	-1.14538900
H	-1.72315700	-1.95175300	1.36181800
H	0.49595100	-1.95583200	1.18765900
H	1.77958100	-2.82412500	-1.45413300
H	2.71476900	1.01007400	-0.22994000
H	4.07698000	2.38238100	2.14089800
H	1.73573300	3.05610100	2.68643600
H	1.05943800	0.58986700	0.99361200
H	-0.79919600	2.38469000	2.73218400
H	-1.20667100	0.12480000	0.70326800
H	-5.32837300	-2.71534700	0.46360300
H	-4.21876700	-2.49285200	1.82555700
H	-4.00970100	-3.87005100	0.72405200
H	3.47210100	-3.03221300	0.62441000
H	3.43465400	-0.79733500	-1.42890600
H	4.40346600	-2.25659800	-1.35129200
H	6.04706800	-0.80075900	-0.69576700
H	5.02367100	1.24192000	-1.13735900
H	5.69471700	1.37761700	0.48707400
H	-1.91721500	-2.93311400	-2.28167300
H	-0.38326500	-3.78789500	-2.08213400
H	-0.38318400	-2.08626500	-2.53862600

H	3.52721700	-1.09760700	1.80811500
H	6.08291400	-0.63549800	1.62225700
C	-1.62091700	2.63718100	-1.91127700
C	-0.22329800	2.34193800	-2.47451300
C	0.10920400	0.84396200	-2.33416800
C	0.82766600	3.23577100	-1.79861200
C	1.37742200	0.40394700	-3.07185700
H	-2.38556800	2.02908500	-2.41079400
H	-1.89156500	3.69139900	-2.04422500
H	-1.66223000	2.41616200	-0.83789200
H	-0.23414200	2.57950000	-3.55018900
H	-0.74063600	0.25860400	-2.71256100
H	0.19877600	0.59041700	-1.26923100
H	1.82716300	3.09145000	-2.22351900
H	0.57139700	4.29560200	-1.91462500
H	0.88528300	3.02163100	-0.72451900
H	2.26848100	0.91182000	-2.68522800
H	1.54121800	-0.67371500	-2.96262300
H	1.30985300	0.62891800	-4.14359900

29

$G_{diethylether}$ = -1316.416085 Hartree

C	-5.55652700	-1.53768000	-1.42853800
O	-3.06419000	-0.07371500	2.62436400
C	-4.38507700	-2.46080100	-1.07747800
O	-3.65686000	-1.13804800	0.76317900
C	-3.19905000	-1.69876400	-0.49584600
O	4.24869300	-1.70005800	1.10550700
C	-1.91910400	-2.51756700	-0.25260300
O	6.06255500	0.40945700	0.62900100
C	-0.79762500	-1.59358500	0.32185700
C	0.36480400	-1.29771600	-0.61200000
C	1.60786900	-1.27085900	-0.09777900
C	2.87823200	-0.76350400	-0.72415300
C	3.13049000	0.77097500	-0.42692900
C	3.04746800	1.19087800	1.03223200
C	1.90908700	1.39785900	1.72167600
C	0.59828000	1.14262400	1.13631300
C	-0.42114400	0.48866500	1.71585000
C	-1.41829300	-0.21297600	0.85087100
C	-2.75709000	-0.45367600	1.51929100

C	-2.15741500	-3.72019600	0.67312200
C	4.08805500	-1.63761100	-0.30683300
C	5.37098200	-1.16751100	-1.01092600
C	5.67009900	0.31153900	-0.75722400
C	4.46143400	1.20007800	-1.08948500
C	0.02513500	-0.83197400	-2.00768700
H	-5.26026400	-0.78814100	-2.17285800
H	-5.90435400	-1.00600900	-0.53785800
H	-6.39655800	-2.10755900	-1.83969100
H	-4.71173800	-3.21729000	-0.35580700
H	-4.03793400	-2.99570600	-1.97167400
H	-2.96372600	-0.86731000	-1.17660600
H	-1.60340100	-2.90137400	-1.23004100
H	-0.38835900	-2.08022300	1.21298500
H	1.73749800	-1.56709300	0.94080700
H	2.81183900	-0.83431400	-1.81952500
H	2.33234100	1.29755900	-0.96583800
H	3.98949300	1.30043500	1.56010800
H	1.96524400	1.63178100	2.78396500
H	0.52140000	1.32667700	0.06698300
H	-0.41318500	0.23433900	2.77331600
H	-1.63149100	0.39377900	-0.03606200
H	-2.87554000	-4.42747700	0.24633400
H	-2.53999000	-3.39722300	1.64743300
H	-1.21764300	-4.25789900	0.83839300
H	3.87351600	-2.66683900	-0.62186600
H	5.26953500	-1.31968100	-2.09304900
H	6.21752300	-1.77120300	-0.66656900
H	6.51958100	0.62196500	-1.38520800
H	4.33688700	1.17725700	-2.18094600
H	4.68821800	2.24401200	-0.82987800
H	-0.55551300	0.10176400	-1.98682300
H	-0.58788700	-1.56370000	-2.54834300
H	0.91562700	-0.64278700	-2.61187800
H	4.78499100	-0.93061800	1.36527900
H	6.20990000	1.34500200	0.83852200
C	-4.19747300	2.30656200	-0.88367900
C	-3.13867800	3.34678200	-0.48550100
C	-1.82674900	3.09894700	-1.25661900
C	-2.94947400	3.36317200	1.03903800
C	-0.67658900	4.05117100	-0.91402300
H	-4.33189700	2.26252700	-1.97190600
H	-5.16951000	2.53623000	-0.43246200

H	-3.91434200	1.30416600	-0.53821500
H	-3.50889600	4.33884900	-0.78898100
H	-2.04154700	3.15882000	-2.33312000
H	-1.49673800	2.06699700	-1.07450300
H	-2.28004200	4.16910700	1.35775700
H	-3.91081900	3.51173200	1.54429800
H	-2.53110400	2.41918800	1.40568900
H	-0.32675500	3.90369300	0.11280300
H	0.17940200	3.88760800	-1.57987300
H	-0.98522300	5.09885600	-1.01916400

30

$G_{\text{diethylether}}$ = -1316.408770 Hartree

C	-5.81443400	-0.49513200	-1.03708400
O	-3.17333500	-0.44672600	2.89492900
C	-4.89909700	-1.72083100	-1.10870400
O	-3.73613800	-1.21370100	0.89080100
C	-3.51181500	-1.44760100	-0.52416200
O	3.28538500	-2.02584300	1.42430900
C	-2.48227200	-2.59222600	-0.77170000
O	5.59408700	-0.55043600	0.88980600
C	-1.11525200	-2.21224700	-0.24359900
C	-0.19519100	-1.39795100	-0.79097900
C	0.95331300	-0.91824800	0.07967800
C	2.35361200	-0.92789200	-0.59951900
C	3.07410300	0.45458000	-0.56237800
C	2.97179100	1.07577000	0.81104200
C	1.78475100	1.09574500	1.42176600
C	0.58705400	0.51587300	0.71769600
C	-0.61974500	0.31316500	1.57701100
C	-1.85253600	0.30680400	1.05258100
C	-2.95745200	-0.43145100	1.70463500
C	-2.93964600	-3.92795800	-0.16458500
C	3.21542500	-2.06902000	0.00524200
C	4.61779300	-2.11025600	-0.62413700
C	5.34350800	-0.76818800	-0.51357700
C	4.50522700	0.36132700	-1.12346700
C	-0.37297700	-0.74806300	-2.14240100
H	-5.39415300	0.34696000	-1.60177800
H	-5.94386500	-0.17549600	0.00134600
H	-6.80310200	-0.71676500	-1.45302300

H	-5.35523200	-2.55508800	-0.56495400
H	-4.77490000	-2.04345600	-2.15107900
H	-3.12144600	-0.53872700	-0.99473300
H	-2.44013500	-2.69942100	-1.86463200
H	-0.91385800	-2.57825900	0.76332200
H	1.02736100	-1.58020400	0.94679800
H	2.24002800	-1.17980600	-1.66226900
H	2.51304100	1.10731900	-1.24931000
H	3.85700300	1.49688700	1.28049200
H	1.65436900	1.52800500	2.41089300
H	0.32482500	1.17245500	-0.12209800
H	-0.46703400	-0.07953700	2.58194700
H	-1.99104400	0.65263900	0.03419400
H	-3.86438800	-4.29030800	-0.62358500
H	-3.11513800	-3.81552400	0.91067400
H	-2.16998300	-4.69368500	-0.30814100
H	2.70088100	-3.01131000	-0.22334700
H	4.53989300	-2.37135900	-1.68756400
H	5.20836700	-2.88843900	-0.12963700
H	6.30694100	-0.82955800	-1.04331800
H	4.45682400	0.18975400	-2.20740100
H	5.02099200	1.32362600	-0.98918200
H	-0.57154000	0.32882100	-2.03952000
H	-1.20236000	-1.17983100	-2.70833000
H	0.52763100	-0.83375800	-2.76200100
H	3.93340600	-1.33591000	1.65289900
H	5.99579900	0.32585700	0.99475700
C	-2.63397600	4.57886200	-0.09984100
C	-1.10181600	4.52665100	-0.19070600
C	-0.66814900	3.59945700	-1.34248600
C	-0.50455200	4.10954000	1.16190800
C	0.84184100	3.55654700	-1.60304700
H	-3.08288000	4.90456300	-1.04619500
H	-2.96332500	5.26910900	0.68548200
H	-3.03977400	3.58729900	0.14133100
H	-0.73789900	5.53875700	-0.42860000
H	-1.17900900	3.91932200	-2.26151500
H	-1.03760200	2.58437100	-1.13230200
H	0.59041400	4.09899700	1.14749000
H	-0.81927700	4.80003500	1.95326900
H	-0.84609000	3.10587200	1.44265300
H	1.39650900	3.16873300	-0.74193200
H	1.07397500	2.91740200	-2.46364500

H	1.22875500	4.55975300	-1.82134400

TS-1

$G_{diethylether}$ = -1561.057602 Hartree

Imaginary frequency: -374.0 cm^{-1}

C	-3.77566200	1.63855300	1.39769700
O	-1.54441000	-0.29914500	-2.39134700
C	-2.63564100	2.57246200	0.98226200
O	-1.85133200	1.14018200	-0.73922300
C	-1.41436300	1.81468800	0.46064100
O	5.77963200	0.57858500	-1.58546400
C	-0.17666500	2.71716900	0.17522900
O	7.51815200	-1.40399100	-0.50105300
C	1.05649300	1.94876700	-0.24950500
C	2.10868900	1.54103600	0.53341900
C	3.29453100	1.04982100	-0.09997000
C	4.51690500	0.75996900	0.51956600
C	4.74387200	-1.34862700	0.84419100
C	4.50026400	-2.02649500	-0.36508700
C	3.25220800	-2.07718800	-0.99133300
C	2.09784900	-1.53508700	-0.44172800
C	0.92075500	-1.20976000	-1.14672800
C	-0.06834700	-0.48323400	-0.54050900
C	-1.17913800	0.09579000	-1.29010700
C	-0.48720300	3.79343500	-0.88209300
C	5.80147000	1.08238900	-0.26122100
C	7.08376800	0.70800700	0.50110400
C	7.27771300	-0.78241700	0.77592000
C	6.09874400	-1.42438800	1.52843400
C	2.05382600	1.58092900	2.04594400
H	-3.44542600	0.90572100	2.14397500
H	-4.15758500	1.08508400	0.53845000
H	-4.60681200	2.20658400	1.82911600
H	-2.98929900	3.26170500	0.20772800
H	-2.30873000	3.18555100	1.83327100
H	-1.13923000	1.07214000	1.21854500
H	0.02853100	3.21998400	1.12944100
H	1.19202600	1.86777900	-1.32602800
H	3.29900200	1.02969100	-1.18703600
H	4.59132600	0.99855400	1.58221600
H	3.91520600	-1.29825900	1.54455200

H	5.35285800	-2.39541000	-0.92548600
H	3.20745400	-2.45199900	-2.01310600
H	2.09544700	-1.29481600	0.61812700
H	0.86038600	-1.39125100	-2.21818800
H	-0.02821900	-0.33700700	0.52951800
H	-1.27219300	4.47701700	-0.54609900
H	-0.81861400	3.32608200	-1.81535000
H	0.40805700	4.38688100	-1.09453900
H	5.81946700	2.17593500	-0.37521700
H	7.09326200	1.23839800	1.46215900
H	7.94297600	1.06293100	-0.07816700
H	8.17600500	-0.90026100	1.40221900
H	6.03877500	-0.94006400	2.51124700
H	6.34661700	-2.47878600	1.72886600
H	1.07504400	1.88655100	2.42203100
H	2.80157000	2.26963700	2.45913000
H	2.26897100	0.58991100	2.46628900
H	6.14122600	-0.32325800	-1.55303000
H	7.66030300	-2.35287200	-0.35729400
C	-7.61867800	1.00986500	1.02410300
C	-7.64687500	0.04166000	-0.17324600
C	-6.28020900	-0.43577700	-0.56994900
C	-5.55277000	-0.06030500	-1.67421900
C	-5.44043700	-1.34474500	0.16989800
C	-4.22277800	-1.47697100	-0.55744900
C	-5.58771100	-2.04278400	1.38095900
N	-4.32882100	-0.69967600	-1.68949300
C	-3.16694000	-2.27771800	-0.10499100
C	-4.53777800	-2.82993700	1.83994500
C	-3.33908500	-2.94413500	1.10374300
H	-7.04268700	1.90889500	0.77777200
H	-7.14109300	0.54227100	1.89226500
H	-8.63127000	1.31623800	1.31347900
H	-8.12665000	0.53489100	-1.02844300
H	-8.28241800	-0.81925600	0.07925900
H	-5.82427400	0.61845700	-2.47208100
H	-3.53466200	-0.45884900	-2.27623400
H	-6.50981800	-1.96374300	1.95181000
H	-2.25359600	-2.37519400	-0.68265200
H	-4.64006700	-3.37057300	2.77748400
H	-2.53729300	-3.57241700	1.48361200

TS-2

$G_{diethylether}$ = -1561.071134 Hartree

Imaginary frequency: -264.2 cm^{-1}

C	-3.88078700	1.73162300	1.31174900
O	-1.48730200	-0.18477900	-2.45207600
C	-2.68059100	2.61649600	0.96335100
O	-1.90169000	1.17083500	-0.75179300
C	-1.48043100	1.80979800	0.47176800
O	5.63240800	0.74962300	-1.49227200
C	-0.18908300	2.64556700	0.23461000
O	7.51623100	-1.12853900	-0.60043000
C	0.98812900	1.80028600	-0.21678800
C	2.02928800	1.32104800	0.57335800
C	3.10618300	0.66955300	-0.06016400
C	4.40745800	0.32784300	0.61855800
C	4.72527400	-1.21958200	0.77244000
C	4.51517100	-1.98376200	-0.50429600
C	3.30132100	-2.03625600	-1.08377100
C	2.14479300	-1.39952800	-0.47637300
C	0.99659900	-1.03569900	-1.18729200
C	-0.03528100	-0.35135500	-0.57180500
C	-1.16591000	0.18744200	-1.33103100
C	-0.41763400	3.78091300	-0.77967900
C	5.56709500	1.07135000	-0.11145500
C	6.91226000	0.83850100	0.59511600
C	7.25289500	-0.64561700	0.73343700
C	6.11394800	-1.41541900	1.41478400
C	1.91303200	1.27521000	2.08445000
H	-3.61634500	0.96238700	2.04723700
H	-4.25668700	1.21983400	0.42390000
H	-4.69781400	2.33014200	1.72883400
H	-2.96859100	3.34036200	0.19313500
H	-2.35649800	3.19275300	1.84086600
H	-1.26727700	1.04123400	1.22468300
H	0.04453300	3.09391600	1.20902900
H	1.18209600	1.84086800	-1.28573500
H	3.20611200	0.80918000	-1.13155000
H	4.39119000	0.71437000	1.64676400
H	3.99265500	-1.58170000	1.50717100
H	5.37405100	-2.42630400	-0.99804000
H	3.17943200	-2.48855900	-2.06688800

H	2.05083600	-1.47668500	0.60539000
H	1.00275400	-1.08228200	-2.27510200
H	-0.12416600	-0.38984000	0.50641100
H	-1.15177400	4.50616200	-0.41715600
H	-0.77859500	3.37620400	-1.73119600
H	0.51874800	4.31625600	-0.96842000
H	5.33743700	2.14338400	-0.07370800
H	6.88036500	1.28084300	1.59901900
H	7.70431200	1.34311300	0.03181300
H	8.16751700	-0.75361800	1.33663800
H	6.07485000	-1.07923900	2.45995400
H	6.35791100	-2.48671000	1.44755700
H	2.65089900	1.92600200	2.57080500
H	2.08902800	0.25871100	2.45922600
H	0.92525800	1.58114900	2.43701200
H	6.13192600	-0.08274900	-1.56775700
H	7.69875400	-2.07975000	-0.55011100
C	-7.61576800	0.91761400	0.92904400
C	-7.59594900	-0.11906700	-0.20964400
C	-6.20756100	-0.54540900	-0.58920100
C	-5.51003800	-0.20352800	-1.72305200
C	-5.31495100	-1.36094800	0.19640000
C	-4.09918600	-1.47787700	-0.53637500
C	-5.41450500	-1.98453000	1.45198300
N	-4.25504000	-0.78089700	-1.71417600
C	-3.00145200	-2.19713000	-0.04834600
C	-4.32088200	-2.68491000	1.94849900
C	-3.12626300	-2.78910200	1.20439200
H	-7.09122300	1.83087500	0.62699400
H	-7.11002500	0.52878500	1.81977600
H	-8.64262400	1.18426600	1.20707300
H	-8.10547500	0.29661300	-1.08846500
H	-8.18273400	-0.99681800	0.09710700
H	-5.82316000	0.40866000	-2.55860700
H	-3.47902400	-0.52393800	-2.31760900
H	-6.33403100	-1.91524500	2.02813800
H	-2.09239100	-2.29461400	-0.63292400
H	-4.38622500	-3.16675300	2.92071800
H	-2.29095900	-3.35325600	1.61181800

TS-3

$G_{\text{diethylether}}$ = -1504.731459 Hartree

Imaginary frequency: -370.9 cm^{-1}

C	5.19211700	-3.69010000	-0.20387100
O	3.11214800	-0.15512600	2.34701300
C	4.49001100	-2.72203200	-1.16064300
O	3.58542100	-1.36017300	0.54646500
C	3.18750000	-2.16954000	-0.57863600
O	-3.40500500	1.45622600	-0.09867300
C	2.33280000	-1.35960500	-1.59807900
O	-5.88989700	0.49819400	0.76667500
C	1.04622500	-0.82698800	-1.01030000
C	-0.22262300	-1.33193900	-1.15469900
C	-1.31829000	-0.53236100	-0.69703300
C	-2.68188200	-0.74465900	-0.93288300
C	-3.65939600	-1.63851600	0.75455200
C	-3.37799700	-0.87771500	1.90480000
C	-2.10186200	-0.73260000	2.45294500
C	-0.97807200	-1.40173700	1.98623600
C	0.35499000	-1.00143100	2.21717800
C	1.39689700	-1.59900600	1.56458200
C	2.72936900	-0.99239100	1.54892300
C	3.12861700	-0.18938800	-2.20780300
C	-3.55537600	0.49666000	-1.14949500
C	-5.02918900	0.16313400	-1.41837000
C	-5.76728000	-0.50803100	-0.26032700
C	-5.08561700	-1.79096000	0.25015900
C	-0.51104000	-2.68550500	-1.76634200
H	4.55898900	-4.55878300	0.01733600
H	5.42321500	-3.19053100	0.74177200
H	6.12877600	-4.05945300	-0.63510500
H	5.15628700	-1.88458000	-1.39276900
H	4.25807000	-3.22368600	-2.10963000
H	2.59097300	-3.02216100	-0.23084700
H	2.09881100	-2.07553600	-2.39713400
H	1.13100200	0.16016200	-0.56416300
H	-1.05128500	0.41673300	-0.24932000
H	-2.93871700	-1.56681400	-1.60341700
H	-3.04971200	-2.52337300	0.59632200
H	-4.15556500	-0.22606300	2.28957400
H	-1.95668000	0.04257000	3.20492600
H	-1.11686900	-2.27979300	1.36082900
H	0.54992600	-0.09928100	2.79278200
H	1.21945300	-2.51907200	1.02548500

H	3.96774800	-0.54585900	-2.81326300
H	3.51808900	0.45637000	-1.41613600
H	2.48098200	0.42058700	-2.84466200
H	-3.16865500	1.00734900	-2.04045400
H	-5.08475500	-0.49445900	-2.29476900
H	-5.55758200	1.08910000	-1.66991300
H	-6.77581800	-0.78048400	-0.60755700
H	-5.10766500	-2.51663100	-0.57257600
H	-5.71323800	-2.22222200	1.04620100
H	0.39862700	-3.24339300	-1.99963900
H	-1.09265200	-2.59295200	-2.69262700
H	-1.10564500	-3.30226900	-1.07950200
H	-4.09414700	1.28752900	0.56826700
H	-6.34404800	0.10702900	1.52947000
C	4.28218500	3.74766000	1.44315200
C	3.85369500	2.57988300	0.53752400
C	2.41373400	2.69818800	0.08476600
C	2.08503800	3.15138100	-1.20070600
C	1.35623500	2.38972000	0.95192500
C	0.76262300	3.28210700	-1.61678500
C	0.02359500	2.52835300	0.55681900
C	-0.28316800	2.96913500	-0.74008600
O	-1.55523900	3.10398600	-1.20580200
H	5.32667500	3.63904000	1.75863100
H	3.65969400	3.78470400	2.34449700
H	4.17629300	4.70846400	0.92539000
H	3.99420000	1.63722600	1.07795100
H	4.50999000	2.54710300	-0.34255400
H	2.88500200	3.39665200	-1.89660800
H	1.58565700	2.01574600	1.94643500
H	0.51589900	3.61839400	-2.61936500
H	-0.78348300	2.27486100	1.24071700
H	-2.20029100	2.70261900	-0.58501100

TS-4

$G_{\text{diethylether}}$ = -1504.746543 Hartree

Imaginary frequency: -268.7 cm^{-1}

C	5.30135000	-3.59189700	-0.33757600
O	3.01788600	-0.15506900	2.32459000
C	4.51878200	-2.62120500	-1.22699700
O	3.59872700	-1.38219500	0.56723300
C	3.21196900	-2.16265600	-0.58070800

O	-3.28837200	1.43095800	-0.06103700
C	2.27772600	-1.35338700	-1.52628100
O	-5.83353000	0.62274400	0.63548500
C	1.02927900	-0.84932400	-0.82856900
C	-0.25210400	-1.37844000	-0.96762800
C	-1.31201000	-0.70844900	-0.32697000
C	-2.77720300	-0.92562200	-0.60876800
C	-3.62866400	-1.58312800	0.55531400
C	-3.41972700	-0.90441400	1.88111000
C	-2.20014100	-0.83283300	2.44383000
C	-1.03663700	-1.46144200	1.83845000
C	0.27248100	-1.03478900	2.08140800
C	1.35302900	-1.60772200	1.43787800
C	2.68651000	-0.99991400	1.51317000
C	3.01060500	-0.16603700	-2.17753400
C	-3.40761700	0.41691200	-1.06740800
C	-4.86841600	0.23699200	-1.49939500
C	-5.72472000	-0.38455400	-0.39513500
C	-5.10763800	-1.69009100	0.12672100
C	-0.44200400	-2.74986800	-1.58628700
H	4.72133000	-4.50154200	-0.13647700
H	5.53768600	-3.12270300	0.62222300
H	6.24072100	-3.89212400	-0.81418300
H	5.13318000	-1.74088900	-1.44312500
H	4.27920100	-3.09262900	-2.18956500
H	2.67221800	-3.05913800	-0.24857900
H	1.98862500	-2.06372200	-2.31271000
H	1.09959000	0.16894600	-0.45928900
H	-1.08601100	0.28902900	0.02686000
H	-2.88217100	-1.61060300	-1.46132900
H	-3.24939900	-2.61192300	0.63316000
H	-4.25809500	-0.39284100	2.34299700
H	-2.04585000	-0.22952000	3.33769300
H	-1.17291500	-2.46173400	1.43125600
H	0.43315100	-0.09001100	2.59641500
H	1.25231400	-2.60411100	1.02586400
H	3.79792900	-0.50378900	-2.85865200
H	3.46055800	0.46889900	-1.40917400
H	2.30804900	0.45203000	-2.74434100
H	-2.82402200	0.79318700	-1.91376100
H	-4.90599400	-0.41416300	-2.38120600
H	-5.28727900	1.20805700	-1.78399000
H	-6.73058600	-0.59582100	-0.78794900

H	-5.19508700	-2.43203500	-0.67863200
H	-5.70943400	-2.07450100	0.96235700
H	-1.44422800	-3.14958900	-1.41034700
H	0.26898700	-3.47307400	-1.16687000
H	-0.27820800	-2.73607300	-2.67156900
H	-4.05724800	1.35710300	0.53517300
H	-6.31741400	0.24526100	1.38640400
C	4.23407100	3.78836800	1.48947800
C	3.82551300	2.60629500	0.59330000
C	2.39688300	2.71684500	0.10430500
C	2.09909700	3.14041500	-1.19847200
C	1.31879200	2.42639600	0.95212100
C	0.78710200	3.25532300	-1.65096900
C	-0.00418300	2.55055500	0.52106300
C	-0.27947100	2.95794000	-0.79390500
O	-1.54019800	3.08294100	-1.29156800
H	5.27030300	3.68351700	1.83233200
H	3.58970200	3.84201900	2.37450800
H	4.14292000	4.74049700	0.95322000
H	3.95141300	1.67171600	1.15120800
H	4.50251400	2.55820100	-0.27030400
H	2.91546700	3.37278900	-1.87961100
H	1.52384900	2.07637400	1.96059000
H	0.56480700	3.57007800	-2.66624200
H	-0.82776800	2.31722100	1.19180900
H	-2.19483700	2.70097600	-0.66705400

TS-5

$G_{diethylether}$ = -1675.283748 Hartree

Imaginary frequency: -371.2 cm^{-1}

C	-5.25081300	-0.89716700	-1.21871400
O	-2.72527100	-0.99482100	2.97170800
C	-4.19811900	-2.00960500	-1.24068500
O	-3.27684400	-1.46314700	0.86820900
C	-2.90916600	-1.60780500	-0.52057800
O	4.51282400	-1.52442400	1.00874300
C	-1.74406500	-2.62684500	-0.69894700
O	6.22396400	0.73564400	0.68260800
C	-0.41767200	-2.14408700	-0.14915500
C	0.56344000	-1.44837800	-0.81431500
C	1.84227700	-1.28965700	-0.19199400

C	2.99873100	-0.75657000	-0.77530500
C	3.31067200	1.28312100	-0.16949500
C	3.25124400	1.35842700	1.23450300
C	2.08915800	1.13055000	1.97740900
C	0.85283900	0.88336100	1.39691000
C	-0.26675800	0.29743600	2.02530400
C	-1.34213800	-0.07721900	1.26708200
C	-2.46339600	-0.85580200	1.79282100
C	-2.08532600	-3.99614000	-0.08418200
C	4.34542800	-1.38991100	-0.39229200
C	5.54830000	-0.71699600	-1.07548300
C	5.79622900	0.74139900	-0.69308600
C	4.57711800	1.65115500	-0.92434300
C	0.32953400	-0.82320100	-2.17275900
H	-4.87396700	0.01051100	-1.70664300
H	-5.51491400	-0.64066800	-0.18839700
H	-6.16287100	-1.20707800	-1.74042900
H	-4.60567900	-2.91175700	-0.77188200
H	-3.94481900	-2.26880500	-2.27726400
H	-2.59048700	-0.64029200	-0.92685900
H	-1.64646700	-2.75066200	-1.78611000
H	-0.15578200	-2.54148600	0.82907400
H	1.97435600	-1.74980200	0.78431700
H	2.93363200	-0.49791600	-1.83369900
H	2.40391900	1.54598400	-0.70629700
H	4.18366300	1.44293700	1.78310900
H	2.18322100	1.02276300	3.05731300
H	0.72398600	1.12227800	0.34455400
H	-0.22288200	0.00741300	3.07352300
H	-1.38473500	0.25257100	0.24002600
H	-2.96545600	-4.44389400	-0.55534200
H	-2.28982100	-3.89381100	0.98653300
H	-1.24686400	-4.68921500	-0.20984800
H	4.31409900	-2.42151100	-0.77201400
H	5.41266300	-0.76754100	-2.16369500
H	6.44778500	-1.29182200	-0.82962300
H	6.61947600	1.12271700	-1.31791200
H	4.37193000	1.65092000	-2.00230300
H	4.86309000	2.68545700	-0.67486500
H	-0.67985000	-1.00151200	-2.54826300
H	1.03943300	-1.20316000	-2.91811900
H	0.46053900	0.26513500	-2.12409600
H	4.90880100	-0.69601900	1.32801700

H	6.39107300	1.65229200	0.95286600
C	-4.07479900	2.43729900	2.10814000
C	-3.80342900	2.23259500	0.61317100
C	-2.50288200	2.91372700	0.17417800
S	-1.87808400	2.41457300	-1.48315600
C	-3.27934100	2.91635700	-2.54513200
H	-5.04073200	2.00547600	2.39132700
H	-4.09040100	3.50230200	2.37127700
H	-3.30434400	1.94419500	2.71103200
H	-4.64099900	2.62451600	0.02213700
H	-3.74858000	1.16090800	0.39920700
H	-1.68912900	2.64845900	0.85711700
H	-2.60762200	4.00536200	0.19855000
H	-4.17327800	2.32015400	-2.34169000
H	-3.50903200	3.97837400	-2.41090700
H	-2.97340000	2.75077400	-3.58169300

TS-6

$G_{diethylether}$ = -1675.303264 Hartree

Imaginary frequency: -273.0 cm^{-1}

C	5.22130600	1.16383100	-1.26107900
O	2.55020200	0.98993500	2.90725900
C	4.09754800	2.20490500	-1.23415600
O	3.20056100	1.48652100	0.83693300
C	2.83763900	1.67936500	-0.54687900
O	-4.44050200	1.47895900	0.98821800
C	1.59972500	2.61432100	-0.66596500
O	-6.29722200	-0.60163000	0.64857200
C	0.34007700	2.00159600	-0.07965700
C	-0.62852200	1.26368100	-0.75542800
C	-1.78661900	0.87940700	-0.04512200
C	-3.02981300	0.31130800	-0.68136500
C	-3.38917800	-1.18505800	-0.29369400
C	-3.33583900	-1.42723200	1.18842900
C	-2.18793700	-1.25559000	1.86995800
C	-0.95838500	-0.87915300	1.19331100
C	0.13408100	-0.28661000	1.83974800
C	1.23815000	0.11829500	1.11579200
C	2.34369000	0.87150900	1.71588300
C	1.85490000	3.98835600	-0.02228500
C	-4.22611300	1.26958800	-0.39945700

C	-5.50373100	0.79299000	-1.10957200
C	-5.88468000	-0.63921100	-0.73371500
C	-4.71390400	-1.60370900	-0.96358800
C	-0.34119800	0.65698800	-2.11302300
H	4.90445300	0.25756800	-1.79261200
H	5.50052800	0.87661100	-0.24292700
H	6.11162000	1.55681700	-1.76395900
H	4.44152800	3.10657700	-0.71607600
H	3.83087300	2.50032300	-2.25771600
H	2.58907100	0.71266000	-1.00267800
H	1.45301800	2.75844600	-1.74496800
H	0.03757100	2.42515400	0.87477600
H	-1.98829800	1.40794600	0.88109500
H	-2.90076700	0.29129600	-1.77179700
H	-2.60174500	-1.79014300	-0.76386000
H	-4.25404800	-1.66318600	1.71638000
H	-2.17784700	-1.31436500	2.95745300
H	-0.76177400	-1.34962900	0.23120800
H	0.03223300	0.05977500	2.86710600
H	1.39437700	-0.30077300	0.13143700
H	2.67648900	4.51865800	-0.51311500
H	2.10925400	3.87394100	1.03653300
H	0.95963500	4.61487300	-0.09491100
H	-3.95337300	2.25306500	-0.80166700
H	-5.35700900	0.83721000	-2.19638000
H	-6.32779900	1.46794100	-0.85505300
H	-6.73880100	-0.96131100	-1.34946000
H	-4.55897200	-1.67339000	-2.04898500
H	-4.99399300	-2.61306000	-0.62994300
H	0.57280000	1.05152900	-2.56210400
H	-1.15798200	0.83119300	-2.82280400
H	-0.20800500	-0.43039500	-2.03126000
H	-4.96836000	0.72728100	1.31097400
H	-6.49935300	-1.50746000	0.93005000
C	4.16317400	-2.19749400	2.25814400
C	3.96913100	-2.06352100	0.74297200
C	2.73800800	-2.83648400	0.25907800
S	2.19448800	-2.44955500	-1.45634500
C	3.69856200	-2.88159400	-2.40205700
H	5.08451900	-1.69865700	2.57740100
H	4.22543000	-3.24982400	2.56243400
H	3.33344300	-1.72906200	2.79832600
H	4.86072100	-2.42882100	0.21757400

H	3.86669100	-1.00571600	0.48155300
H	1.86913000	-2.58754000	0.87786800
H	2.90009200	-3.91812400	0.34293400
H	4.52796000	-2.20742400	-2.17080100
H	3.99996900	-3.91522600	-2.20353600
H	3.45018500	-2.78506100	-3.46245600

TS-7

$G_{diethylether}$ = -1504.707803 Hartree

Imaginary frequency: -373.4659 cm^{-1}

C	-4.23985500	2.78597000	-2.03533300
O	-0.89189000	4.61408700	1.05744500
C	-3.87824000	1.77975500	-0.93915200
O	-2.21212800	3.14596700	0.03709700
C	-2.40003700	1.84401900	-0.55040000
O	4.23192900	-0.65835900	2.10037500
C	-1.95328100	0.71021100	0.41705100
O	6.64976700	-0.86174300	0.60164300
C	-0.46104700	0.67613600	0.66838100
C	0.49092900	-0.03954400	-0.01458400
C	1.80638900	-0.14057500	0.53888700
C	2.84614900	-0.96307500	0.08781200
C	4.26855200	0.15363600	-1.07379300
C	4.65391000	1.29706700	-0.34984100
C	3.79124300	2.35665300	-0.05310200
C	2.48662300	2.43414500	-0.52167500
C	1.45603100	3.23822800	0.00943000
C	0.16529600	3.07048600	-0.41135700
C	-0.97182900	3.68274600	0.28131300
C	-2.68833900	0.79410400	1.76608200
C	3.75888600	-1.59690300	1.14926900
C	4.88471000	-2.45616300	0.54893900
C	5.90903900	-1.70548100	-0.30079700
C	5.27979500	-0.91304800	-1.45992700
C	0.20210300	-0.70998000	-1.33961800
H	-3.66765800	2.59473500	-2.95220300
H	-4.01852000	3.80556100	-1.70611100
H	-5.30459400	2.72907900	-2.28724900
H	-4.48803000	1.97100800	-0.04937600
H	-4.09645200	0.75677400	-1.26992200
H	-1.81315400	1.75921100	-1.47414800

H	-2.24931700	-0.22292200	-0.07667900
H	-0.14323600	1.14018100	1.59984600
H	1.97913400	0.36074300	1.48808200
H	2.60610700	-1.64380100	-0.73115100
H	3.49374600	0.29466000	-1.82162300
H	5.62000200	1.28902200	0.14439700
H	4.11975600	3.09501700	0.67737600
H	2.20097600	1.79249200	-1.35122200
H	1.65041500	3.87918200	0.86743100
H	-0.02007500	2.46440400	-1.28830700
H	-3.77008300	0.69672000	1.64237200
H	-2.48544000	1.75194500	2.25614700
H	-2.35457400	-0.01308600	2.42580600
H	3.12201900	-2.28406400	1.72493300
H	4.43767700	-3.24482400	-0.07024600
H	5.41712400	-2.94636800	1.37123800
H	6.59752200	-2.44573800	-0.73869300
H	4.80406300	-1.64134300	-2.12898800
H	6.09098000	-0.45294900	-2.04667200
H	-0.82206600	-0.53710100	-1.67717500
H	0.34886100	-1.79393200	-1.28028000
H	0.87793200	-0.32805000	-2.11698700
H	5.04837600	-0.27461600	1.73829200
H	7.30947600	-0.37103500	0.08668100
C	-6.06043700	-1.38700200	2.18464100
C	-5.78434500	-1.28031800	0.68266800
C	-4.52205600	-1.96591900	0.18697800
C	-3.55608400	-2.51153000	1.04252000
C	-4.28398200	-2.05121200	-1.19217600
C	-2.40047900	-3.11827100	0.55064300
C	-3.13550100	-2.65426500	-1.70262800
C	-2.19150800	-3.20026600	-0.82646700
O	-1.04765200	-3.82239500	-1.26041300
H	-6.12646100	-2.43292800	2.50563600
H	-7.00827900	-0.89811900	2.43242500
H	-5.27492600	-0.90225600	2.77496600
H	-5.73856900	-0.21776200	0.40389600
H	-6.64228000	-1.68876000	0.13090800
H	-3.69486800	-2.46365700	2.11740600
H	-5.01493300	-1.63916600	-1.88500500
H	-1.65588100	-3.53549200	1.22093600
H	-2.97846500	-2.71091400	-2.77853400
H	-1.03232200	-3.80991800	-2.22995600

TS-8

$G_{diethylether}$ = -1504.736966 Hartree

Imaginary frequency: -273.5 cm^{-1}

C	-5.48045100	2.18726800	-1.71915300
O	-2.14198700	4.53477000	1.12475800
C	-4.84342800	1.28940600	-0.65452400
O	-3.33837300	2.93584900	0.14262500
C	-3.36702800	1.61380100	-0.42595100
O	3.56490900	0.39884600	2.02502900
C	-2.62431800	0.59897900	0.48724600
O	6.00265500	0.30027600	0.61053200
C	-1.15119400	0.91691600	0.64604400
C	-0.10223800	0.36988400	-0.08679200
C	1.21373800	0.68871300	0.30775500
C	2.45239400	-0.03509000	-0.15049300
C	3.43285300	0.79312200	-1.07997000
C	3.75634500	2.14756100	-0.51296000
C	2.78608000	3.05172700	-0.28191000
C	1.40006900	2.78582400	-0.63140300
C	0.30736300	3.44132400	-0.04926400
C	-0.98722500	3.09780800	-0.38837800
C	-2.15291900	3.60390400	0.34591700
C	-3.27998500	0.49289200	1.87487100
C	3.20144500	-0.60569500	1.08919700
C	4.40262900	-1.46165600	0.65997800
C	5.37930400	-0.69365400	-0.23044300
C	4.66478000	-0.06470900	-1.43486300
C	-0.38183000	-0.29373500	-1.41597900
H	-4.98303100	2.06490500	-2.68965000
H	-5.39832400	3.23867300	-1.42773000
H	-6.54114700	1.94885900	-1.85345100
H	-5.38246100	1.40190900	0.29224000
H	-4.92063300	0.23355300	-0.94805800
H	-2.87162200	1.61685100	-1.40593200
H	-2.71869300	-0.36973100	-0.02030500
H	-0.88731900	1.37118900	1.59805900
H	1.32570700	1.13577600	1.29080200
H	2.16124200	-0.90845000	-0.74684300
H	2.87448900	0.93905500	-2.01571100
H	4.77596300	2.35574000	-0.20519600

H	3.01227700	3.97624500	0.24758700
H	1.22453100	2.28983000	-1.58474500
H	0.45883600	4.05744700	0.83566300
H	-1.15919700	2.58494900	-1.32717400
H	-4.30464900	0.11349700	1.81297200
H	-3.30933600	1.47383600	2.36060300
H	-2.70788800	-0.18815000	2.51408900
H	2.49522000	-1.25246900	1.62248600
H	4.03449100	-2.33804800	0.11396700
H	4.92928600	-1.81512600	1.55296300
H	6.15593400	-1.38277600	-0.59790400
H	4.34747300	-0.88847100	-2.08913200
H	5.37933400	0.53300200	-2.01899200
H	-1.21508800	-0.99891300	-1.35587800
H	0.47954200	-0.84590100	-1.79633200
H	-0.64202500	0.46135500	-2.17290800
H	4.39728300	0.79212300	1.70931500
H	6.59284500	0.83743600	0.05933800
C	-4.36424900	-4.39593100	1.04392000
C	-3.80997400	-3.13345600	0.36073400
C	-2.32722100	-3.23457600	0.07367300
C	-1.37716300	-2.83476800	1.02480200
C	-1.85626700	-3.77526900	-1.12903400
C	-0.01100500	-2.95389600	0.78835700
C	-0.49006600	-3.90081400	-1.38428000
C	0.43537300	-3.48148200	-0.42510000
O	1.79388200	-3.53919200	-0.63085900
H	-4.21130000	-5.27887800	0.41292300
H	-5.43759400	-4.29863300	1.24356100
H	-3.85375200	-4.57667600	1.99663000
H	-4.00275700	-2.26319400	1.00029200
H	-4.35626700	-2.95788500	-0.57528600
H	-1.71324500	-2.40822500	1.96658900
H	-2.56766300	-4.09732400	-1.88634400
H	0.71627900	-2.62750000	1.52336600
H	-0.14574500	-4.30871900	-2.33320200
H	1.96544000	-3.95193300	-1.49133500

TS-9

$G_{diethylether}$ = -1331.216651 Hartree

Imaginary frequency: -379.1 cm^{-1}

C	-7.51654500	0.55297400	-0.51721100
O	-4.36929900	-1.03475700	2.87305700
C	-6.50567100	-0.42533800	-1.12283700
O	-5.25632000	-0.67758400	0.86794000
C	-5.11251500	-0.27804700	-0.50727900
O	2.42667000	-0.79796500	0.00686400
C	-4.01029300	-1.10872300	-1.22978000
O	4.22992800	1.36799400	0.31057000
C	-2.61518600	-0.86264100	-0.69519000
C	-1.67118000	0.01267300	-1.17455100
C	-0.32826100	-0.08452300	-0.69103000
C	0.79205000	0.60026100	-1.18432400
C	1.30011600	2.26765700	0.05567000
C	1.38780400	1.83668000	1.39454500
C	0.29778900	1.37219500	2.13439000
C	-1.00791100	1.38021800	1.65975700
C	-2.07805300	0.61145600	2.16396000
C	-3.26933900	0.55977700	1.49393400
C	-4.30643900	-0.42303300	1.82614900
C	-4.31168900	-2.61810200	-1.18194100
C	2.12363700	-0.16144900	-1.22913200
C	3.29078100	0.68308000	-1.76531800
C	3.67706400	1.89128600	-0.91384100
C	2.50728900	2.85692400	-0.65668400
C	-2.00537100	1.08697000	-2.18765600
H	-7.21043700	1.59352300	-0.68433300
H	-7.60058900	0.39433200	0.56200500
H	-8.50816800	0.41980300	-0.96285000
H	-6.85323600	-1.45306600	-0.97359200
H	-6.42243800	-0.26529200	-2.20617000
H	-4.83535500	0.78254000	-0.56496400
H	-4.05082000	-0.78044800	-2.27714200
H	-2.27593100	-1.58839500	0.04074400
H	-0.12301300	-0.87063900	0.03118500
H	0.63128300	1.21909200	-2.06885500
H	0.36366400	2.73186400	-0.23990700
H	2.37520600	1.70585000	1.82594400
H	0.49705200	0.87225500	3.08141500
H	-1.23894400	2.00636000	0.80180300
H	-1.91382900	-0.07132400	2.99555600
H	-3.45005400	1.26866600	0.69685000
H	-5.24627600	-2.86071000	-1.69629200
H	-4.39649900	-2.95659600	-0.14418100

H	-3.50639100	-3.18231900	-1.66368800
H	1.98689700	-0.99018200	-1.93697200
H	3.03288700	1.04104000	-2.77039300
H	4.16888200	0.03723100	-1.86482000
H	4.46305700	2.44725100	-1.44743900
H	2.19776700	3.25783400	-1.63012500
H	2.88577800	3.71782900	-0.08366200
H	-3.06938900	1.11545800	-2.43236900
H	-1.45042000	0.94619400	-3.12383700
H	-1.73287900	2.07871200	-1.80336600
H	2.83793200	-0.12783500	0.57948600
H	4.54929300	2.10962700	0.84823900
N	5.09622600	-2.39066600	0.27700800
C	5.58328800	-1.67473100	1.47691200
C	6.95986300	-1.08942000	1.09493400
C	6.82687800	-0.86060400	-0.41795000
C	6.01238300	-2.09613700	-0.83992300
H	4.89696200	-0.86150400	1.75539900
H	4.15767600	-2.06953300	0.05603100
H	5.65734300	-2.34965200	2.33981900
H	7.20022500	-0.17996000	1.65640500
H	7.75280400	-1.82144300	1.29493800
H	6.25070800	0.05463500	-0.59725800
H	7.79008500	-0.77577200	-0.93422300
H	5.45373000	-1.95164200	-1.77333400
H	6.69109000	-2.94704400	-1.00618200

TS-10

$G_{diethylether}$ = -1331.241480 Hartree

Imaginary frequency: -273.3 cm^{-1}

C	-7.18419300	0.48115700	0.41155600
O	-3.51427800	-2.73800900	1.30906400
C	-6.20386700	0.33842800	-0.75662100
O	-4.73568800	-1.12476800	0.38594500
C	-4.76201300	0.14721900	-0.28615200
O	2.63991400	0.02009200	-1.09797700
C	-3.70234200	0.19551700	-1.42411300
O	4.64913300	1.32558700	0.30650500
C	-2.27947100	0.06598400	-0.90888900
C	-1.37995500	1.10134500	-0.65973400
C	-0.05368200	0.76599300	-0.32966300

C	1.08480400	1.75179300	-0.26814800
C	1.73368500	1.97916500	1.16414200
C	2.03498000	0.69321600	1.88192800
C	1.05534200	-0.17951800	2.18452900
C	-0.33673900	0.10993200	1.88486600
C	-1.32598700	-0.86786300	1.75172900
C	-2.61728600	-0.53069200	1.38733900
C	-3.61867300	-1.55551600	1.05823700
C	-3.95923200	-0.88307900	-2.49176800
C	2.16070800	1.34347500	-1.31586300
C	3.31515500	2.35509800	-1.37049700
C	3.97315900	2.56209900	-0.00606700
C	2.93510500	2.94200100	1.05821000
C	-1.85122600	2.53390000	-0.50443500
H	-6.94068300	1.35516300	1.02885700
H	-7.14593900	-0.40566600	1.05113200
H	-8.21126800	0.60138700	0.05064900
H	-6.49132300	-0.51753700	-1.37631100
H	-6.24128400	1.22976800	-1.39731700
H	-4.53430900	0.95060800	0.42779900
H	-3.82428100	1.18212800	-1.89076300
H	-1.84076500	-0.91736100	-1.05799400
H	0.26627200	-0.24621700	-0.55268700
H	0.72247600	2.74335800	-0.57147600
H	0.96029600	2.51194200	1.73480500
H	3.06839100	0.44078300	2.09369800
H	1.30106900	-1.15779700	2.59455800
H	-0.67164100	1.13226700	2.05280300
H	-1.04127900	-1.91787100	1.70647900
H	-2.95490200	0.48759800	1.53982400
H	-4.91937200	-0.73391200	-2.99451200
H	-3.96410800	-1.87836200	-2.03531100
H	-3.17367700	-0.85840100	-3.25429400
H	1.67196700	1.31445700	-2.29639300
H	2.93693800	3.32253400	-1.72417300
H	4.06486200	2.00553000	-2.08813000
H	4.72120500	3.36573200	-0.08071500
H	2.56868600	3.94691600	0.80782900
H	3.42416300	3.03039700	2.03843100
H	-1.41173800	3.19324500	-1.26373200
H	-1.56012100	2.93729300	0.47433000
H	-2.93696500	2.62726700	-0.58121600
H	3.30375500	0.06971600	-0.38680300

H	5.05887600	1.40515100	1.18173100
N	4.32068500	-2.44390500	-1.70508600
C	3.47380200	-3.42589800	-1.01456900
C	3.26676700	-2.87859700	0.41467500
C	4.62320200	-2.20041200	0.72406100
C	5.28430100	-1.99847500	-0.68172000
H	2.53898500	-3.58021300	-1.56326000
H	3.72997300	-1.64691000	-1.94091600
H	4.00228400	-4.38976100	-0.97910700
H	2.46390600	-2.13332000	0.40224000
H	2.99919500	-3.65163500	1.14411200
H	4.49124400	-1.24981300	1.25309600
H	5.24901700	-2.83496200	1.36191800
H	5.57101300	-0.95804900	-0.86455900
H	6.19358200	-2.60897600	-0.75552400

TS-11

$G_{diethylether}$ = -1406.492189 Hartree

Imaginary frequency: -357.9 cm^{-1}

C	-7.09343800	-0.99847500	-0.79205600
O	-4.19486400	-0.53464800	2.92213100
C	-5.83393900	-1.80786800	-1.11411600
O	-4.69828900	-1.18127300	0.86575800
C	-4.56298500	-1.14790400	-0.57100300
O	2.50688900	0.84999200	0.40689200
C	-3.25018200	-1.84219000	-1.03283800
O	3.55005100	3.41135500	0.50883600
C	-2.00125400	-1.08464000	-0.64361400
C	-1.19051500	-0.33367500	-1.42430500
C	0.07469700	0.15586700	-0.86048000
C	1.09209000	0.70195300	-1.55187200
C	0.68561700	3.66488400	-0.25872500
C	0.50133600	3.76224300	1.07465400
C	-0.51934900	3.02788800	1.80318300
C	-1.64705500	2.52007200	1.24803800
C	-2.50812300	1.55924000	1.87641900
C	-3.44555500	0.88597800	1.17099500
C	-4.12863800	-0.28659400	1.73536000
C	-3.14900600	-3.28531200	-0.50022900
C	2.49021100	0.91428700	-1.02482200
C	3.26650100	2.14459400	-1.57025500

C	3.16479300	3.50832100	-0.85125700
C	1.82732400	4.29362600	-0.99269300
C	-1.47545700	-0.01993200	-2.87486600
H	-7.04482900	0.00386900	-1.23612200
H	-7.20718300	-0.88403300	0.29006100
H	-7.98943100	-1.49408600	-1.18089000
H	-5.92376500	-2.81305800	-0.68835400
H	-5.72438600	-1.92468500	-2.20055600
H	-4.54898900	-0.11222300	-0.92897400
H	-3.31956900	-1.88467900	-2.12661900
H	-1.69622300	-1.22882800	0.39272000
H	0.20568900	0.01138000	0.21033000
H	0.99509500	0.87559600	-2.62274800
H	0.04193500	3.00352000	-0.83064200
H	1.20606200	4.35323500	1.65713500
H	-0.32321200	2.82959000	2.85742000
H	-1.87409200	2.76252400	0.21070100
H	-2.32990100	1.28128700	2.91415700
H	-3.59035000	1.14123800	0.12919700
H	-3.97104000	-3.90944500	-0.86386900
H	-3.17876800	-3.29079800	0.59408200
H	-2.20905100	-3.74409800	-0.82478700
H	3.05604000	0.04317800	-1.40167600
H	3.02081100	2.27169000	-2.63371600
H	4.32928300	1.87851300	-1.52468000
H	3.92378700	4.14522700	-1.32539600
H	1.59087800	4.40496500	-2.05906900
H	2.02820000	5.29411800	-0.59189800
H	-2.46159300	-0.36127400	-3.19562300
H	-0.72898300	-0.48096500	-3.53480900
H	-1.42569700	1.06239300	-3.04867900
H	3.36160500	0.40943600	0.64497100
H	3.04301000	2.65906100	0.86833400
C	6.75082600	-4.62854500	0.23717500
C	5.90950100	-3.36886100	0.01912800
C	4.67766000	-3.32492600	0.93450600
C	3.91248400	-2.01819200	0.79673200
N	2.56235800	-2.09870000	0.74573900
O	4.51141100	-0.93478600	0.74434500
H	7.62331600	-4.63883600	-0.42510100
H	6.17003600	-5.53788900	0.03827600
H	7.11466200	-4.68652400	1.27044900
H	5.58173600	-3.31378800	-1.02751900

H	6.50541700	-2.46938500	0.20470800
H	5.00011600	-3.39590100	1.98271000
H	4.02379100	-4.18627300	0.74590800
H	2.07604200	-2.98097000	0.78093200
H	2.02619800	-1.23833600	0.67742900

TS-12

$G_{diethylether}$ = -1406.503246 Hartree

Imaginary frequency: -276.7 cm^{-1}

C	-6.50813300	1.47982000	1.59649800
O	-4.71728000	-1.67504600	-1.78913600
C	-5.34516000	2.06016200	0.78610100
O	-4.92450800	0.07224100	-0.42893800
C	-4.28513100	1.00945600	0.45586600
O	2.42268500	-1.46308200	-1.81148900
C	-2.98878400	1.58165700	-0.18504100
O	4.28799300	-3.08286200	-0.47495700
C	-1.94787200	0.51008000	-0.46144600
C	-0.83563900	0.20026700	0.32581100
C	0.10827000	-0.71309000	-0.18538600
C	1.46269400	-0.95815700	0.42144200
C	1.67872600	-2.38369800	1.08151800
C	1.25629700	-3.50866900	0.17921400
C	-0.01944300	-3.62169600	-0.23721700
C	-1.04753900	-2.70112500	0.21746400
C	-2.23884900	-2.45073600	-0.46764500
C	-3.13896600	-1.50270700	-0.00992900
C	-4.29960100	-1.09009000	-0.81133500
C	-3.28351400	2.35400600	-1.48332200
C	2.55870500	-0.66302200	-0.64388800
C	3.96005400	-0.76669400	-0.02528900
C	4.21649900	-2.13249100	0.61092000
C	3.11964000	-2.49373900	1.62153800
C	-0.74974500	0.62461700	1.77934800
H	-6.16036200	1.07788600	2.55648000
H	-6.98883200	0.66708700	1.04383200
H	-7.26188100	2.24607500	1.80717300
H	-5.72475800	2.48753200	-0.14814300
H	-4.86103200	2.87414900	1.34234600
H	-4.00796200	0.50876500	1.39325500
H	-2.58713700	2.28663900	0.55458100

H	-1.89143300	0.20430500	-1.50364500
H	0.05992500	-0.93260600	-1.24775600
H	1.62584600	-0.23590400	1.22785400
H	1.01909300	-2.38711600	1.96048200
H	2.01442100	-4.18195300	-0.20793000
H	-0.28947700	-4.35996900	-0.99088300
H	-1.01026100	-2.40470800	1.26434400
H	-2.36718200	-2.82827600	-1.48082000
H	-3.10600200	-1.21457500	1.03404400
H	-3.91630700	3.22762200	-1.30103600
H	-3.79280200	1.70753000	-2.20565900
H	-2.35042100	2.70657100	-1.93686700
H	2.40977500	0.36635100	-0.98377500
H	4.06296200	0.00913600	0.74217300
H	4.71244400	-0.58096000	-0.79995100
H	5.18730700	-2.11649600	1.13012900
H	3.22975000	-1.81260800	2.47588500
H	3.29445300	-3.50709700	2.01099200
H	-1.62460500	1.19620900	2.09892100
H	0.14327900	1.23301300	1.96238400
H	-0.68221900	-0.25576900	2.43239700
H	2.85582500	-2.31416900	-1.62332800
H	4.40136200	-3.96806900	-0.09518000
C	5.80938900	4.05565300	-0.47699200
C	4.66288000	3.09129700	-0.16555100
C	3.28616700	3.76835400	-0.24290200
C	2.17621400	2.80743100	0.16872200
N	1.09567400	2.74297700	-0.65869600
O	2.25991600	2.14045000	1.19935900
H	6.77924100	3.55017900	-0.41158600
H	5.82120900	4.89582200	0.22833500
H	5.71801200	4.47221700	-1.48777400
H	4.77664800	2.66936200	0.83872100
H	4.68733000	2.24332300	-0.86330300
H	3.11401800	4.17735700	-1.24693900
H	3.25776200	4.61543000	0.45558500
H	1.10865600	3.18160500	-1.56725600
H	0.40022600	2.02032600	-0.49691700

TS-13

$G_{diethylether}$ = -1277.089629 Hartree

Imaginary frequency: -373.8 cm^{-1}

C	5.49267000	-2.87799600	0.92466300
O	3.29835400	1.41350900	1.57653100
C	4.74665000	-2.46455100	-0.34741900
O	3.81800300	-0.50022100	0.58377200
C	3.43452100	-1.73891500	-0.04282600
O	-3.41138000	1.26951800	-1.17871700
C	2.54058500	-1.49302300	-1.29466100
O	-5.79884100	0.66964000	0.27518800
C	1.22270200	-0.82383300	-0.97338600
C	-0.00882100	-1.40849700	-0.81028600
C	-1.16772300	-0.56972000	-0.75257000
C	-2.50531800	-0.98617200	-0.78725200
C	-3.36939400	-1.00917400	1.16477300
C	-3.14561100	0.23596700	1.78360500
C	-1.88290000	0.70464600	2.15353500
C	-0.71885500	-0.04345900	2.03318900
C	0.58941700	0.48343300	2.00802600
C	1.65478200	-0.30163100	1.65994500
C	2.95080400	0.27802400	1.30589300
C	3.27291400	-0.66465200	-2.36748800
C	-3.49963600	-0.09517000	-1.55019900
C	-4.94032200	-0.63300400	-1.51919700
C	-5.59322000	-0.69435500	-0.13916500
C	-4.77977200	-1.50348400	0.88668600
C	-0.18601300	-2.90661800	-0.68190300
H	4.89105700	-3.56664900	1.53143200
H	5.71984200	-1.99919900	1.53560000
H	6.43498600	-3.38094900	0.68222600
H	5.38281300	-1.80816100	-0.95049100
H	4.51859700	-3.34706300	-0.96004800
H	2.86942800	-2.35863800	0.66480500
H	2.34671500	-2.49428200	-1.70197700
H	1.23954000	0.25916500	-1.05232100
H	-1.00393500	0.50138700	-0.83425500
H	-2.67733700	-2.05222000	-0.94866200
H	-2.68345500	-1.80854200	1.43045500
H	-3.96962200	0.94024500	1.82903700
H	-1.78901800	1.75352500	2.43227000
H	-0.80429900	-1.11834200	1.89714200
H	0.73827900	1.55703000	2.10183500
H	1.51979900	-1.37330200	1.61481100
H	4.15867600	-1.18364400	-2.74593600

H	3.58855400	0.29903000	-1.95578100
H	2.60808600	-0.47118200	-3.21579900
H	-3.18396000	-0.12090200	-2.60309200
H	-4.95106100	-1.64319800	-1.94881000
H	-5.55834700	0.00356700	-2.16156800
H	-6.57421300	-1.18373500	-0.24664000
H	-4.73908600	-2.53855600	0.52440300
H	-5.34660700	-1.53857500	1.83058800
H	0.76704200	-3.43879500	-0.64144900
H	-0.76396800	-3.31831100	-1.51911700
H	-0.73683500	-3.15372600	0.23495300
H	-4.00767200	1.39803600	-0.42192900
H	-6.22232300	0.66269500	1.14803200
C	1.94475300	4.53516400	0.53023500
C	1.69079100	3.95732700	-0.86974800
C	2.95023800	3.25408700	-1.39814500
C	0.49180100	2.99969700	-0.85092500
H	2.76585600	5.26237800	0.51631000
H	1.05303000	5.04204200	0.92068900
H	2.22305000	3.73365900	1.22531700
H	1.44892000	4.78757700	-1.55089000
H	3.26505000	2.46346800	-0.70658000
H	2.77489200	2.80715400	-2.38526300
H	3.78585900	3.95848200	-1.49224100
H	0.67999200	2.17219000	-0.15800400
H	-0.42718700	3.50011100	-0.52251200
H	0.29951500	2.57211400	-1.84302100

TS-14

$G_{diethylether}$ = -1277.103721 Hartree

Imaginary frequency: -259.6 cm^{-1}

C	-6.14820100	-2.15719700	-0.68934900
O	-3.24133300	1.82373900	-1.35522700
C	-5.26412400	-1.83180200	0.51839900
O	-4.11962500	-0.04851200	-0.53692000
C	-3.88590300	-1.31389700	0.10784000
O	3.19897800	0.73614900	1.20412000
C	-2.87935200	-1.15748000	1.28418500
O	5.55781000	-0.05170600	-0.09686700
C	-1.51887100	-0.66213000	0.82971200
C	-0.36704700	-1.41887600	0.62781200

C	0.83227400	-0.73599200	0.35013800
C	2.19643700	-1.37330500	0.36681300
C	2.94375400	-1.45605900	-1.03090800
C	2.93291600	-0.15048800	-1.77488600
C	1.77485000	0.42342600	-2.15052000
C	0.49200600	-0.21156300	-1.89803000
C	-0.71285100	0.49047900	-1.81586200
C	-1.89900500	-0.14071300	-1.48777300
C	-3.10224600	0.63247600	-1.15763900
C	-3.41706800	-0.21423000	2.37625300
C	3.07613000	-0.66005400	1.43714100
C	4.45009300	-1.33587400	1.57178700
C	5.20126800	-1.40791200	0.24192600
C	4.34558400	-2.07238800	-0.84496200
C	-0.42572400	-2.92632200	0.47836700
H	-5.69604600	-2.93916500	-1.31249800
H	-6.28585100	-1.26732400	-1.31081800
H	-7.13474900	-2.51086400	-0.37102400
H	-5.75526500	-1.07718200	1.14179600
H	-5.12416600	-2.72569600	1.14109700
H	-3.46196900	-2.02708800	-0.61216400
H	-2.77468000	-2.16363800	1.71133900
H	-1.36355000	0.39968800	0.98670500
H	0.84948000	0.32861100	0.55611900
H	2.10146300	-2.41767700	0.69516200
H	2.36089000	-2.18288000	-1.61382400
H	3.87204400	0.36914700	-1.93426000
H	1.77404600	1.42424500	-2.57826400
H	0.43399500	-1.28811700	-2.04991000
H	-0.69412200	1.57731400	-1.77794600
H	-1.98963100	-1.20875800	-1.64241600
H	-4.32533000	-0.60858300	2.84176100
H	-3.64807300	0.76769500	1.95019000
H	-2.66766200	-0.07750300	3.16286800
H	2.55147100	-0.74607700	2.39643600
H	4.31923000	-2.35745100	1.95090000
H	5.05170200	-0.78248300	2.30051700
H	6.12356700	-1.99374400	0.37757400
H	4.23785800	-3.13086500	-0.57099700
H	4.88588400	-2.05797200	-1.80215700
H	0.10354300	-3.43908300	1.29192600
H	0.04654100	-3.24576800	-0.45953600
H	-1.45127100	-3.30349200	0.46764700

H	3.90006400	0.85646800	0.53917100
H	5.99938800	-0.05914000	-0.96033400
C	-0.48073700	5.32101400	0.12522500
C	0.20143900	4.13889700	0.82738400
C	-0.83974400	3.10620500	1.28221500
C	1.26057400	3.49597800	-0.07870800
H	-1.21271900	5.80794500	0.78105100
H	0.25052200	6.07724900	-0.18596200
H	-1.01476900	4.97902600	-0.77094700
H	0.71314000	4.52100800	1.72377200
H	-1.41036100	2.72225400	0.42642500
H	-0.35681900	2.26045500	1.78752000
H	-1.56023400	3.54735500	1.98205000
H	0.79770700	3.12074200	-1.00017100
H	2.02616600	4.22608600	-0.37091000
H	1.76321000	2.65473200	0.41190700

TS-15

$G_{diethylether}$ = -1367.201086 Hartree

Imaginary frequency: -359.4 cm^{-1}

C	-5.79959700	0.50628900	-1.16277700
O	-3.39619600	-0.35177100	3.03286900
C	-5.04808900	-0.82756700	-1.20648300
O	-4.04703900	-0.54633600	0.91465100
C	-3.70971100	-0.76081100	-0.46785300
O	3.55550700	-2.34416000	0.93764600
C	-2.81776500	-2.02571600	-0.65040800
O	5.71111600	-0.49646200	0.66798000
C	-1.41055800	-1.84767800	-0.12159300
C	-0.31168900	-1.38303200	-0.80222800
C	0.97823700	-1.48811400	-0.18755700
C	2.21415500	-1.18314500	-0.77403900
C	2.96062700	0.69989700	-0.08373800
C	2.94370700	0.70591700	1.32619900
C	1.76965800	0.69904800	2.08514200
C	0.50771900	0.75448600	1.50852200
C	-0.72096400	0.43609700	2.12341600
C	-1.86050700	0.37605000	1.36575000
C	-3.11662700	-0.18215300	1.86281900
C	-3.46036000	-3.26932200	-0.01113200
C	3.39903200	-2.10937900	-0.45135800

C	4.70806300	-1.67961300	-1.13511600
C	5.26933500	-0.32900600	-0.69411700
C	4.26771000	0.82745500	-0.84836100
C	-0.41262800	-0.77734200	-2.18679900
H	-5.21465100	1.30450200	-1.64045800
H	-5.99164100	0.80146800	-0.12699600
H	-6.75911600	0.43980500	-1.68660800
H	-5.66558900	-1.61338000	-0.75856500
H	-4.85492600	-1.12066900	-2.24705100
H	-3.15867100	0.10369700	-0.86099800
H	-2.76800200	-2.18187500	-1.73669200
H	-1.23357200	-2.27189400	0.86405800
H	1.02292300	-1.98624500	0.77775200
H	2.18888300	-0.87158700	-1.81957400
H	2.13321600	1.19287800	-0.58425000
H	3.88004100	0.56194400	1.85573500
H	1.84796400	0.53311300	3.15893600
H	0.44805000	1.04196700	0.46577900
H	-0.75163700	0.09492700	3.15670300
H	-1.82321300	0.74656900	0.35151400
H	-4.43484100	-3.49581400	-0.45472600
H	-3.60552300	-3.11572200	1.06292400
H	-2.81512000	-4.14298800	-0.15033600
H	3.13851900	-3.09017800	-0.87510600
H	4.54708000	-1.64585000	-2.22045800
H	5.46567800	-2.44617800	-0.93874300
H	6.14443800	-0.10046700	-1.32307600
H	4.05037100	0.93172700	-1.91863900
H	4.76848500	1.76371900	-0.55413100
H	-1.44727600	-0.71044500	-2.53420600
H	0.14142700	-1.37725600	-2.92025900
H	0.01319900	0.23208500	-2.20382000
H	4.13383800	-1.64155600	1.27985600
H	6.06028600	0.35343900	0.97924400
C	0.79145400	4.23374900	0.51317300
C	-0.61513500	3.77199700	0.13522200
C	-0.62624700	2.88749800	-1.10600800
N	-1.86502600	2.58182700	-1.59530400
O	0.39467800	2.44164200	-1.62459900
H	1.42967400	3.37952400	0.75230500
H	0.75065800	4.88799900	1.38990000
H	1.25814000	4.78265500	-0.31049200
H	-1.28091400	4.62950200	-0.03263200

H	-1.05943000	3.19033700	0.95446000
H	-2.71445600	2.94366100	-1.18775800
H	-1.93255200	1.98685600	-2.40890000

TS-16

$G_{diethylether}$ = -1367.217317 Hartree

Imaginary frequency: -231.4 cm^{-1}

C	-5.89026000	0.26161900	-1.20462800
O	-3.28777500	-0.40729000	2.98119300
C	-5.05593800	-1.02293100	-1.19719300
O	-4.03625900	-0.58097900	0.89132500
C	-3.71489400	-0.83654400	-0.48731300
O	3.45550100	-2.35037900	0.90320700
C	-2.74184100	-2.04479300	-0.61766000
O	5.70382800	-0.69505700	0.59559200
C	-1.37250200	-1.75378100	-0.03259200
C	-0.26360900	-1.25274900	-0.70743100
C	0.95932600	-1.15800100	-0.01067700
C	2.27788700	-0.82748100	-0.65599700
C	2.95348500	0.53941300	-0.20045800
C	2.99255000	0.69467300	1.29361400
C	1.85067700	0.74242000	2.00888500
C	0.56154900	0.70225400	1.34731200
C	-0.64370200	0.36565600	1.97196600
C	-1.81368000	0.28737300	1.23889900
C	-3.05981700	-0.23875500	1.80050500
C	-3.32341100	-3.31658200	0.02378900
C	3.25320000	-2.02773400	-0.46483600
C	4.58328200	-1.78801000	-1.19797400
C	5.26672500	-0.49056400	-0.76552600
C	4.31886700	0.70807500	-0.89672300
C	-0.39151100	-0.64132400	-2.09012900
H	-5.36369600	1.07094400	-1.72837200
H	-6.08697100	0.59337600	-0.18077400
H	-6.85061600	0.10857800	-1.70846800
H	-5.61546100	-1.82274700	-0.70064800
H	-4.85903000	-1.35477800	-2.22545900
H	-3.22594700	0.04405300	-0.92625300
H	-2.63719100	-2.21661700	-1.69754500
H	-1.17875300	-2.23191200	0.92397200
H	1.05558000	-1.73737100	0.90144800

H	2.13167100	-0.71391600	-1.73808600
H	2.30037000	1.31983300	-0.61000900
H	3.95166400	0.68005800	1.80136500
H	1.87734800	0.72931600	3.09759600
H	0.49591900	1.21202800	0.39371500
H	-0.63962900	-0.04342300	2.98115200
H	-1.85544600	0.77153200	0.27212500
H	-4.25473500	-3.62523300	-0.46079900
H	-3.53214100	-3.14910100	1.08535500
H	-2.61057500	-4.14361300	-0.06027900
H	2.77191200	-2.91095300	-0.90301200
H	4.40108000	-1.73808400	-2.27912300
H	5.25368300	-2.63352700	-1.00976100
H	6.15177700	-0.31851800	-1.39776400
H	4.15125700	0.87550500	-1.96932200
H	4.81454900	1.61507200	-0.52186900
H	-1.43031900	-0.59915100	-2.42800500
H	0.16595000	-1.22377300	-2.83544800
H	0.00597500	0.37933900	-2.11081800
H	4.14427200	-1.74842000	1.23673400
H	6.08498500	0.13630800	0.91829800
C	0.68779100	4.30823000	0.67955200
C	-0.70084900	3.82487000	0.26363200
C	-0.67919900	3.02811400	-1.03579800
N	-1.90810800	2.71206200	-1.54865200
O	0.35568000	2.63911500	-1.57094000
H	1.35394900	3.46291000	0.87020400
H	0.62060200	4.90470800	1.59502100
H	1.14078900	4.92256800	-0.10460500
H	-1.39574100	4.66882900	0.15465100
H	-1.12534100	3.17177000	1.03839900
H	-2.76122200	3.10519000	-1.17903700
H	-1.94633400	2.23081400	-2.43634000

TS-17

$G_{diethylether}$ = -1429.502478 Hartree

Imaginary frequency: -375.6 cm^{-1}

C	-8.09512300	-1.04108400	-0.98699000
O	-5.27211600	-0.29977800	2.93744300
C	-6.83671800	-1.91224400	-1.04401500
O	-5.88723800	-1.01983600	0.92805000

C	-5.60904500	-1.19954300	-0.47252300
O	1.72002700	0.34483900	0.67148800
C	-4.27143700	-1.96668300	-0.69416800
O	3.02046300	2.80025900	0.09996800
C	-3.04820000	-1.20008400	-0.23761000
C	-2.22157000	-0.40420300	-0.99232000
C	-0.97688600	0.02863700	-0.43439900
C	0.05864800	0.69107200	-1.10841100
C	0.04364700	2.79055000	-0.69210700
C	0.00036700	2.97999300	0.70287700
C	-1.08718100	2.62133900	1.50304800
C	-2.28191100	2.12760900	0.99481000
C	-3.25427600	1.40588100	1.71894700
C	-4.29536000	0.80150300	1.06970500
C	-5.16431300	-0.17499400	1.73431400
C	-4.28751100	-3.34714500	-0.01183100
C	1.50137900	0.32054800	-0.73303100
C	2.55593800	1.11648800	-1.51950600
C	2.57150200	2.62141400	-1.26042500
C	1.21517200	3.30094800	-1.51607300
C	-2.58741700	0.04281500	-2.39187100
H	-7.96787200	-0.12190400	-1.57289400
H	-8.31424400	-0.75584400	0.04624700
H	-8.96268300	-1.57636100	-1.38765500
H	-7.00392300	-2.83746600	-0.48246100
H	-6.61765200	-2.19781800	-2.08162500
H	-5.53082400	-0.22421900	-0.97007900
H	-4.20964300	-2.11811200	-1.78027300
H	-2.69999700	-1.45372200	0.76133500
H	-0.74454300	-0.31380700	0.57085200
H	-0.07522100	0.83258400	-2.18246300
H	-0.90527800	2.85629500	-1.21657100
H	0.91608900	3.26817200	1.20893500
H	-0.94776000	2.61555600	2.58336700
H	-2.48907800	2.26979200	-0.06273300
H	-3.09599800	1.18688700	2.77335300
H	-4.47877100	1.05195900	0.03332800
H	-5.06824800	-3.99352200	-0.42368900
H	-4.46753300	-3.23830600	1.06265300
H	-3.32544300	-3.85122300	-0.15078200
H	1.63434800	-0.73266500	-1.01693600
H	2.38657900	0.95820400	-2.59251500
H	3.54515100	0.71321300	-1.28261400

H	3.31012800	3.07776200	-1.93711600
H	0.99076400	3.18080900	-2.58342600
H	1.33106400	4.38437800	-1.35427300
H	-3.58679200	-0.28351300	-2.68796800
H	-1.87663900	-0.33762100	-3.13635100
H	-2.56729700	1.13811700	-2.46498700
H	1.97690500	1.25339200	0.90469400
H	3.06447200	3.75126100	0.28705700
C	7.77397000	-3.79107700	-0.75554300
C	7.79295100	-3.12261700	0.63079200
C	7.07192000	-1.79169200	0.63421800
C	7.75763600	-0.60271300	0.35140900
C	5.69219600	-1.72386600	0.86958800
C	7.08461700	0.61926900	0.30517400
C	5.01139800	-0.50590200	0.82545400
C	5.70911200	0.67226400	0.54187600
H	8.29876100	-4.75354400	-0.73915600
H	8.25649300	-3.14989100	-1.50212400
H	6.74418800	-3.96769300	-1.08661700
H	8.83356300	-2.98202200	0.95022300
H	7.32964000	-3.79648600	1.36284300
H	8.83015100	-0.63732000	0.16884800
H	5.14580100	-2.63818800	1.09414100
H	7.63693900	1.53097500	0.08919800
H	3.94038000	-0.46791500	1.00689400
H	5.17389600	1.61703500	0.50740600

TS-18

$G_{diethylether}$ = -1429.520666 Hartree

Imaginary frequency: -266.2 cm^{-1}

C	7.97050500	1.43252200	-1.06631000
O	5.14167800	0.39676500	2.87716200
C	6.63029700	2.17406000	-1.07075300
O	5.80940800	1.12095100	0.88323100
C	5.49269900	1.32348900	-0.50547100
O	-1.64586000	-0.45807400	0.67770600
C	4.07857000	1.95204700	-0.66676800
O	-3.03615000	-2.82847500	0.16660300
C	2.97072900	1.04751300	-0.15636900
C	2.17509000	0.18731000	-0.91008700
C	1.09833800	-0.45720900	-0.27165200

C	0.01621300	-1.22024200	-0.99028000
C	-0.04916200	-2.77920000	-0.69222200
C	0.01402000	-3.09267200	0.77631400
C	1.08897700	-2.74681000	1.50960900
C	2.24496000	-2.10678500	0.90519600
C	3.17181000	-1.33999400	1.61696500
C	4.20339400	-0.68380800	0.97055400
C	5.06519000	0.27800000	1.67189100
C	3.97932200	3.32277100	0.02631700
C	-1.35602700	-0.53648900	-0.71410200
C	-2.48818800	-1.21502000	-1.50075000
C	-2.58981700	-2.71073800	-1.20370400
C	-1.24603200	-3.41467900	-1.43038700
C	2.58262500	-0.24068800	-2.30628900
H	7.92395700	0.52605200	-1.68308700
H	8.23746700	1.13501300	-0.04788500
H	8.77195600	2.06596400	-1.46139000
H	6.71513300	3.09171100	-0.47907200
H	6.36205800	2.47127000	-2.09354200
H	5.50144900	0.36049300	-1.03413300
H	3.94949700	2.09914300	-1.74742600
H	2.57164000	1.33598300	0.81271700
H	0.77573300	-0.05064400	0.68100000
H	0.18133300	-1.14934600	-2.07390500
H	0.85484800	-3.18854900	-1.16402600
H	-0.85504200	-3.53029200	1.25692200
H	1.07822500	-2.86739200	2.59192900
H	2.55173800	-2.46847200	-0.07475300
H	2.96049200	-1.06298200	2.64860400
H	4.50446500	-1.02234100	-0.01375400
H	4.66480900	4.05101100	-0.41717500
H	4.22100100	3.23070400	1.09031700
H	2.96308500	3.72101700	-0.06223300
H	-1.27630500	0.50437700	-1.04881700
H	-2.30526300	-1.09185700	-2.57591900
H	-3.43861300	-0.72627800	-1.26701400
H	-3.34868000	-3.16138100	-1.86072300
H	-1.05069300	-3.39608200	-2.51125200
H	-1.32952100	-4.47511800	-1.15324300
H	3.54402400	0.18086200	-2.60867900
H	1.84226700	0.06002400	-3.05839100
H	2.67416300	-1.33306800	-2.36899500
H	-2.01385800	-1.32022900	0.94225500

H	-3.08718500	-3.77012100	0.39354100
C	-7.54924200	3.92947700	-0.86407200
C	-7.57930700	3.30238100	0.54128100
C	-6.90257900	1.94889200	0.57852700
C	-7.62981400	0.77533900	0.33826800
C	-5.52352400	1.84242000	0.80309300
C	-6.99778200	-0.46909000	0.32299100
C	-4.88359900	0.60178500	0.78945600
C	-5.62237500	-0.56069400	0.54833300
H	-8.04168900	4.90900300	-0.87212300
H	-8.05864800	3.28339300	-1.58822900
H	-6.51682400	4.06192200	-1.20733100
H	-8.62141900	3.20575000	0.87195200
H	-7.08820800	3.98142000	1.25009600
H	-8.70242000	0.83997200	0.16457800
H	-4.94514000	2.74448200	0.99474700
H	-7.58189200	-1.36808500	0.13990200
H	-3.81257100	0.53380800	0.96168000
H	-5.11970200	-1.52378900	0.53776300

TS-19

$G_{diethylether}$ = -1316.381614 Hartree

Imaginary frequency: -371.1 cm^{-1}

C	-5.72642800	-0.16181900	-0.99963900
O	-3.06007600	-1.16566200	2.99233000
C	-4.78622200	-1.34674200	-1.24099700
O	-3.74519300	-1.20726500	0.87701700
C	-3.44158900	-1.17703600	-0.53059800
O	3.97083300	-2.08711000	0.81312500
C	-2.38413800	-2.25732400	-0.90987600
O	5.92485100	-0.00655300	0.71358500
C	-1.01268200	-1.99484500	-0.32465400
C	0.04552800	-1.35213200	-0.91989300
C	1.33330100	-1.40905700	-0.29817700
C	2.54154500	-0.94812500	-0.83776300
C	3.08092200	0.94870700	-0.00346800
C	3.04552400	0.86042400	1.40161400
C	1.87487800	0.66927100	2.14063800
C	0.60979800	0.63810900	1.56943800
C	-0.55497900	0.09247200	2.15008900
C	-1.69163000	-0.04601700	1.40104300

C	-2.84800300	-0.82598000	1.84593500
C	-2.84659200	-3.67040500	-0.50964200
C	3.80973400	-1.77011600	-0.55842000
C	5.07490500	-1.15866700	-1.18468700
C	5.48689800	0.20897800	-0.64173700
C	4.37333800	1.26691800	-0.73701700
C	-0.10007800	-0.59426600	-2.22376800
H	-5.29117900	0.77246200	-1.37481500
H	-5.91651000	-0.03839200	0.07074200
H	-6.68683000	-0.31283100	-1.50432400
H	-5.25957500	-2.27054900	-0.89170800
H	-4.59427100	-1.46695500	-2.31565000
H	-3.04286100	-0.19351200	-0.81000400
H	-2.31569800	-2.21219100	-2.00503900
H	-0.80337300	-2.52315300	0.60273700
H	1.41290200	-1.98516400	0.62022600
H	2.50057400	-0.56849500	-1.86048900
H	2.20530300	1.37504300	-0.48250900
H	3.98769300	0.77558300	1.93300800
H	1.97064300	0.41426000	3.19532000
H	0.49360400	1.03267300	0.56485100
H	-0.51386000	-0.36167300	3.13840500
H	-1.73715200	0.44330100	0.44040500
H	-3.76799300	-3.95623400	-1.02575400
H	-3.03189500	-3.71942900	0.56827700
H	-2.07703400	-4.40772100	-0.76099400
H	3.65774100	-2.73900200	-1.05587800
H	4.92547900	-1.06667700	-2.26834800
H	5.90666800	-1.85352000	-1.02588600
H	6.34094500	0.56951900	-1.23671300
H	4.15942000	1.41861000	-1.80263800
H	4.77323900	2.22663200	-0.37287400
H	-1.14381100	-0.46448300	-2.51785500
H	0.41895900	-1.10778200	-3.04362200
H	0.33951000	0.40656400	-2.14610700
H	4.46784500	-1.35606900	1.21753500
H	6.19869900	0.84689100	1.08488600
C	-3.51606600	3.10672200	-0.39331600
C	-2.10359700	3.71092500	-0.38332400
C	-1.16842700	2.89360100	-1.29548300
C	-1.58033500	3.82071700	1.05708300
C	0.26702000	3.42082300	-1.39219200
H	-3.90683900	3.01262900	-1.41429300

H	-4.21758400	3.72371400	0.17982400
H	-3.51672200	2.10728700	0.06002800
H	-2.16477600	4.72764500	-0.80230800
H	-1.60617200	2.85641400	-2.30303800
H	-1.14813800	1.85443400	-0.94138400
H	-0.61509000	4.33579800	1.10620300
H	-2.28508700	4.38169200	1.68194700
H	-1.45556400	2.82856700	1.50655100
H	0.78712300	3.36159100	-0.43007200
H	0.85078400	2.84468500	-2.12098600
H	0.28183800	4.46974600	-1.71325000

TS-20

$G_{diethylether}$ = -1316.391448 Hartree

Imaginary frequency: -256.5 cm^{-1}

C	-5.74118900	-0.38638600	-1.08128900
O	-2.93442400	-1.21047100	2.93686200
C	-4.73645800	-1.52849100	-1.26338400
O	-3.71438000	-1.23240600	0.85103400
C	-3.41017800	-1.25064600	-0.55602600
O	3.89131100	-2.05191300	0.78962100
C	-2.28297800	-2.27842700	-0.86892800
O	5.95125400	-0.14383600	0.63809200
C	-0.95865500	-1.90939700	-0.22470700
C	0.09347000	-1.21721600	-0.81636000
C	1.29279900	-1.06357900	-0.09088200
C	2.58017300	-0.55607100	-0.68729100
C	3.09849700	0.83732100	-0.13018500
C	3.10369600	0.89418300	1.37151100
C	1.96148700	0.75670300	2.07094800
C	0.68149700	0.60634900	1.40188100
C	-0.45296900	0.04209900	1.99738000
C	-1.61774800	-0.12182700	1.27358800
C	-2.76948900	-0.87317300	1.78301600
C	-2.67943600	-3.70532900	-0.45064600
C	3.67502300	-1.65703800	-0.55622400
C	4.98095500	-1.22743800	-1.24500500
C	5.51482400	0.10363600	-0.71472500
C	4.44505200	1.20145300	-0.78798900
C	-0.09538300	-0.47097400	-2.12430500
H	-5.35100300	0.55359500	-1.49097900

H	-5.94959200	-0.22863300	-0.01888500
H	-6.68628800	-0.60871100	-1.58838200
H	-5.16062500	-2.45993800	-0.87354900
H	-4.52958000	-1.68743600	-2.33030000
H	-3.06742000	-0.25773600	-0.87601600
H	-2.16649100	-2.25481100	-1.96074100
H	-0.71523600	-2.48398300	0.66510700
H	1.44167800	-1.71676200	0.76241800
H	2.43901700	-0.39225900	-1.76430000
H	2.36768900	1.57194000	-0.49539700
H	4.05434200	0.96207800	1.89011300
H	1.98679700	0.67417400	3.15654500
H	0.51823900	1.21686200	0.51761200
H	-0.36374400	-0.47102000	2.95355900
H	-1.75449200	0.46361500	0.37616000
H	-3.55632400	-4.05912000	-1.00103600
H	-2.91220600	-3.73877000	0.61873900
H	-1.85675400	-4.40138300	-0.64542300
H	3.29512900	-2.55246300	-1.06352300
H	4.80910000	-1.12549400	-2.32420700
H	5.73633000	-2.00671600	-1.09839400
H	6.38388000	0.41167300	-1.31653200
H	4.27562500	1.41948800	-1.85140500
H	4.83464800	2.12887600	-0.34465600
H	0.38554200	-0.99723200	-2.95982100
H	0.34603000	0.53029300	-2.07966600
H	-1.14920300	-0.34294000	-2.38293800
H	4.50418500	-1.40499200	1.18158100
H	6.25674400	0.69474700	1.01769600
C	-3.65371500	3.01354300	-0.31651300
C	-2.27349900	3.68830600	-0.28097600
C	-1.30632500	2.97239700	-1.24350000
C	-1.74249200	3.74511100	1.15977100
C	0.10364600	3.56829500	-1.31573800
H	-4.04688900	2.95385600	-1.33919100
H	-4.38113700	3.56307700	0.29187300
H	-3.60230400	1.99251600	0.08301300
H	-2.39111300	4.72233100	-0.64139300
H	-1.74843800	2.97393800	-2.24985500
H	-1.23574700	1.91671400	-0.95152100
H	-0.80904400	4.31359200	1.23112200
H	-2.47213400	4.22615600	1.82157700
H	-1.55338700	2.73827800	1.55080100

H	0.63244500	3.48003200	-0.36029000
H	0.70781700	3.05828800	-2.07631000
H	0.07020100	4.63287100	-1.57861600

References

1. Case, D.A.; Belfon, K.; Ben-Shalom, I.; Brozell, S.R.; Cerutti, D.S.; Cheatham, T.E.; Cruzeiro, V.; Darden, T.; Duke, R.; Giambasu, G. *Amber 2020 Reference Manual*; University of California: San Francisco, CA, USA, **2020**.
2. Bayly, C. I.; Cieplak, P.; Cornell, W.; Kollman, P. A., A well-behaved electrostatic potential based method using charge restraints for deriving atomic charges: the RESP model. *J. Phys. Chem.* **1993,** 97, 40, 10269–10280.
3. Besler, B. H.; Merz Jr, K. M.; Kollman, P. A., Atomic charges derived from semiempirical methods. *J. Comput. Chem.* **1990,** 11, 431-439.
4. Singh, U. C.; Kollman, P. A., An approach to computing electrostatic charges for molecules. *J. Comput. Chem.* **1984,** 5, 129-145.
5. Frisch, M. J.; Trucks, G. W.; Schlegel, H. B.; Scuseria, G. E.; Robb, M. A.; Cheeseman, J. R.; Scalmani, G.; Barone, V.; Petersson, G. A.; Nakatsuji, H.; Li, X.; Caricato, M.; Marenich, A. V.; Bloino, J.; Janesko, B. G.; Gomperts, R.; Mennucci, B.; Hratchian, H. P.; Ortiz, J. V.; Izmaylov, A. F.; Sonnenberg, J. L.; Williams; Ding, F.; Lipparini, F.; Egidi, F.; Goings, J.; Peng, B.; Petrone, A.; Henderson, T.; Ranasinghe, D.; Zakrzewski, V. G.; Gao, J.; Rega, N.; Zheng, G.; Liang, W.; Hada, M.; Ehara, M.; Toyota, K.; Fukuda, R.; Hasegawa, J.; Ishida, M.; Nakajima, T.; Honda, Y.; Kitao, O.; Nakai, H.; Vreven, T.; Throssell, K.; Montgomery Jr., J. A.; Peralta, J. E.; Ogliaro, F.; Bearpark, M. J.; Heyd, J. J.; Brothers, E. N.; Kudin, K. N.; Staroverov, V. N.; Keith, T. A.; Kobayashi, R.; Normand, J.; Raghavachari, K.; Rendell, A. P.; Burant, J. C.; Iyengar, S. S.; Tomasi, J.; Cossi, M.; Millam, J. M.; Klene, M.; Adamo, C.; Cammi, R.; Ochterski, J. W.; Martin, R. L.; Morokuma, K.; Farkas, O.; Foresman, J. B.; Fox, D. J., Gaussian 16, Revision A. 03, Gaussian. *Inc., Wallingford CT* **2016**, 3.
6. Wang, J.; Wolf, R. M.; Caldwell, J. W.; Kollman, P. A.; Case, D. A., Development and testing of a general amber force field. *J. Comput. Chem.* **2004,** 26, 114-114.
7. Maier, J. A.; Martinez, C.; Kasavajhala, K.; Wickstrom, L.; Hauser, K. E.; Simmerling, C., ff14SB: Improving the Accuracy of Protein Side Chain and Backbone Parameters from ff99SB. *J. Chem. Theory Comput.* **2015,** 11, 3696-3713.
8. Jorgensen, W. L.; Chandrasekhar, J.; Madura, J. D.; Impey, R. W.; Klein, M. L., Comparison of simple potential functions for simulating liquid water. *J .Chem. Phys.* **1983,** 79, 926-935.
9. Grimme, S.; Antony, J.; Ehrlich, S.; Krieg, H., A consistent and accurate ab initio parametrization of density functional dispersion correction (DFT-D) for the 94 elements H-Pu. *J. Chem. Phys.* **2010,** 132, 154104.
10. Marenich, A. V. C., C. J.; Truhlar, D. G, Universal Solvation Model Based on Solute Electron Density and on a Continuum Model of the Solvent Defined by the Bulk Dielectric Constant and Atomic Surface Tensions. *J. Phys. Chem. B* **2009,** 113, 6378-6396.
11. Yu Takano; Houk, K. N., Benchmarking the Conductor-like Polarizable Continuum Model (CPCM) for Aqueous Solvation Free Energies of Neutral and Ionic Organic Molecules. *J. Chem. Theory Comput.* **2015,** 1, 70-77.
12. Barone, V.; Cossi, M., Quantum calculation of molecular energies and energy gradients in solution by a conductor solvent model. *J. Phys. Chem. A* **1998,** 102, 1995-2001.
13. Maurizio Cossi, N. R., Giovanni Scalmani, Vincenzo Barone, Energies, Structures, and Electronic Properties of Molecules in Solution with the C-PCM Solvation Model. *J. Comput. Chem.* **2003,** 24, 669–681.
14. Biswas, B.; Collins, S. C.; Singleton, D. A., Dynamics and a unified understanding of competitive [2,3]- and [1,2]-sigmatropic rearrangements based on a study of ammonium ylides. *J. Am. Chem. Soc.* **2014,** 136, 3740-3743.
15. Roe, D. R.; Cheatham, T. E., 3rd, PTRAJ and CPPTRAJ: Software for Processing and Analysis of Molecular Dynamics Trajectory Data. *J. Chem. Theory Comput.* **2013,** 9, 3084-3095.
